# Supplementary material for: Honey bee success predicted by landscape composition in Ohio, USA
Source: PeerJ. 2015 Mar 19;3:e838. doi: 10.7717/peerj.838 (PMC4369331; doi:10.7717/peerj.838)
Supplement: Table S1 — This spreadsheet contains the data compiled from our beekeeper surveys, including the variables experience, syrup, Food, Wax, AdultPop, and BroodPop. [file peerj-03-838-s001.doc]

| year | id | years_experience | syrup | Wax | Food | BroodPop | AdultPop |
| --- | --- | --- | --- | --- | --- | --- | --- |
| 2012 | 9.12 | 1 | 2.764 | 20 | 3 | 17 | 19 |
| 2012 | 11.12 | 3 | 5.528 | 31.05 | 24.84 | 6.21 | 19.32 |
| 2012 | 12.12 | 1 | 2.764 | 16.56 | 0 | 0 | 2.76 |
| 2012 | 17.12 | 3 | 2.2112 | 9 | 1 | 8 | 8 |
| 2012 | 20.12 | 1 | 22.112 | 22.08 | 11.04 | 11.04 | 16.56 |
| 2012 | 21.12 | 0 | 24.876 | 28.9 | 17.9 | 6 | 24.9 |
| 2012 | 26.12 | 1 | 22.112 | 0 | 0 | 0 | 12 |
| 2012 | 27.12 | 12 | 0 | 30.35 | 19.21 | 7 | 25.52 |
| 2012 | 28.12 | 0 | 35.3792 | 23.15 | 9.66 | 6.9 | 17.94 |
| 2012 | 30.12 | 0 | 2.2112 | 20.7 | 13.8 | 2.76 | 19.32 |
| 2012 | 33.12 | 0 | 5.528 | 29.66 | 15.52 | 4 | 20.14 |
| 2012 | 36.12 | 4 | 0.5528 | 17.42 | 25.8 | 8 | 16.76 |
| 2012 | 40.12 | 6 | 19.0696 | 7 | 8.38 | 10 | 18 |
| 2012 | 41.12 | 0 | 14.3728 | 13.8 | 6.9 | 4.14 | 12.42 |
| 2012 | 42.12 | 0 | 13.2672 | 16.1 | 0 | 11.04 | 9.66 |
| 2012 | 43.12 | 0 | 10.5032 | 7 | 3.69 | 6 | 13.45 |
| 2012 | 44.12 | 3 | 4.4224 | 16 | 17 | 3 | 20 |
| 2012 | 45.12 | 20 | 5.528 | 33.21 | 17.83 | 6.38 | 30.83 |
| 2012 | 47.12 | 1 | 2.2112 | 21.39 | 10.35 | 11.04 | 15.18 |
| 2012 | 50.12 | 1 | 16.0312 | 16 | 13 | 3 | 9 |
| 2012 | 52.12 | 0 | 6.6336 | 15 | 8 | 4 | 7 |
| 2012 | 53.12 | 2 | 3.3168 | 11 | 7 | 1 | 16 |
| 2012 | 54.12 | 10 | 0 | 12.07 | 20.21 | 5 | 8 |
| 2012 | 101.12 | 1 | 3.3168 | 18 | 11 | 4 | 6 |
| 2012 | 102.12 | 5 | 2.2112 | 1 | 8 | 7 | 10 |
| 2012 | 104.12 | 8 | 4.9752 | 31.52 | 8.76 | 5 | 11 |
| 2012 | 107.12 | 0 | 19.9008 | 34.04 | 25.04 | 3 | 23.9 |
| 2012 | 108.12 | 1 | 11.04 | 8.28 | 19.32 | 2.76 | 6.21 |
| 2012 | 111.12 | 0 | 3.3168 | 13 | 2 | 4 | 15 |
| 2012 | 112.12 | 2 | 2.2112 | 7.97 | 2 | 2 | 0 |
| 2012 | 113.12 | 0 | 27.64 | 25.87 | 13.865 | 8 | 21.9 |
| 2012 | 115.12 | 14 | 5.528 | 18.63 | 5.52 | 11.04 | 16.56 |
| 2013 | 3.13 | 3 | 16.584 | 24 | 19 | 8 | 21 |
| 2013 | 7.13 | 4 | 5.3868 | 0 | 13.8 | 1.38 | 13.8 |
| 2013 | 11.13 | 0.2 | 17.6896 | 14 | 2 | 10 | 10 |
| 2013 | 14.13 | 0 | 15.4784 | 29.21 | 6.21 | 6 | 9 |
| 2013 | 16.13 | 1 | 3.3168 | 4.52 | 16.66 | 8 | 19.14 |
| 2013 | 22.13 | 0 | 17.1368 | 18.35 | 10.21 | 5.07 | 10.83 |
| 2013 | 23.13 | 6 | 5.3168 | 4.76 | 5 | 2 | 5 |
| 2013 | 26.13 | 3 | 12.428 | 6.83 | 19.73 | 0 | 13.9 |
| 2013 | 28.13 | 0 | 1.1056 | 30 | 14 | 6 | 16 |
| 2013 | 36.13 | 4 | 2.2112 | 12 | 11 | 0 | 4 |
| 2013 | 37.13 | 3 | 4.4224 | 1 | 12 | 0 | 16 |
| 2013 | 38.13 | 30 | 10.6336 | 15 | 17.9 | 9 | 24.83 |
| 2013 | 39.13 | 0 | 5.528 | 20.7 | 15.87 | 4.83 | 8.97 |
| 2013 | 40.13 | 7 | 11.056 | 14.9 | 17.9 | 6 | 28.87 |
| 2013 | 42.13 | 0 | 13.2672 | 15 | 7 | 4 | 11 |
| 2013 | 43.13 | 6 | 3.3168 | 13.69 | 9.69 | 5 | 9 |
| 2013 | 46.13 | 2 | 2.4876 | 19.9 | 17.9 | 5 | 15.795 |
| 2013 | 47.13 | 9 | 0 | 6 | 5 | 0 | 5 |
