# Supplementary material for: Honey bee success predicted by landscape composition in Ohio, USA
Source: PeerJ. 2015 Mar 19;3:e838. doi: 10.7717/peerj.838 (PMC4369331; doi:10.7717/peerj.838)
Supplement: Table S2 — This spreadsheet contains the land cover data for each study site, derived from the National Land Cover Database 2006 land cover layer. [file peerj-03-838-s002.doc]

| year | id | scale | classID | class | landcover | landprop |
| --- | --- | --- | --- | --- | --- | --- |
| 2012 | 9.12 | 0.5 | 21 | urban_open | 230400 | 0.3018867925 |
| 2012 | 9.12 | 0.5 | 22 | urban_low | 112500 | 0.1474056604 |
| 2012 | 9.12 | 0.5 | 23 | urban_med | 32400 | 0.0424528302 |
| 2012 | 9.12 | 0.5 | 24 | urban_high | 20700 | 0.0271226415 |
| 2012 | 9.12 | 0.5 | 41 | dec_forest | 195300 | 0.2558962264 |
| 2012 | 9.12 | 0.5 | 42 | eg_forest | 13500 | 0.0176886792 |
| 2012 | 9.12 | 0.5 | 43 | mix_forest | 6300 | 0.008254717 |
| 2012 | 9.12 | 0.5 | 71 | grass_herb | 900 | 0.0011792453 |
| 2012 | 9.12 | 0.5 | 81 | pasture_hay | 121500 | 0.1591981132 |
| 2012 | 9.12 | 0.5 | 90 | woody_wetland | 29700 | 0.0389150943 |
| 2012 | 11.12 | 0.5 | 21 | urban_open | 19800 | 0.0259433962 |
| 2012 | 11.12 | 0.5 | 22 | urban_low | 1800 | 0.0023584906 |
| 2012 | 11.12 | 0.5 | 41 | dec_forest | 393300 | 0.5153301887 |
| 2012 | 11.12 | 0.5 | 42 | eg_forest | 24300 | 0.0318396226 |
| 2012 | 11.12 | 0.5 | 52 | scrub | 20700 | 0.0271226415 |
| 2012 | 11.12 | 0.5 | 82 | crop | 303300 | 0.3974056604 |
| 2012 | 12.12 | 0.5 | 21 | urban_open | 263700 | 0.3467455621 |
| 2012 | 12.12 | 0.5 | 22 | urban_low | 466200 | 0.6130177515 |
| 2012 | 12.12 | 0.5 | 23 | urban_med | 25200 | 0.0331360947 |
| 2012 | 12.12 | 0.5 | 24 | urban_high | 5400 | 0.0071005917 |
| 2012 | 17.12 | 0.5 | 21 | urban_open | 402300 | 0.5327771156 |
| 2012 | 17.12 | 0.5 | 22 | urban_low | 339300 | 0.4493444577 |
| 2012 | 17.12 | 0.5 | 23 | urban_med | 12600 | 0.0166865316 |
| 2012 | 17.12 | 0.5 | 24 | urban_high | 900 | 0.0011918951 |
| 2012 | 20.12 | 0.5 | 21 | urban_open | 75600 | 0.0989399293 |
| 2012 | 20.12 | 0.5 | 41 | dec_forest | 42300 | 0.0553592462 |
| 2012 | 20.12 | 0.5 | 81 | pasture_hay | 153000 | 0.2002355713 |
| 2012 | 20.12 | 0.5 | 82 | crop | 493200 | 0.6454652532 |
| 2012 | 21.12 | 0.5 | 21 | urban_open | 77400 | 0.1018957346 |
| 2012 | 21.12 | 0.5 | 22 | urban_low | 18900 | 0.0248815166 |
| 2012 | 21.12 | 0.5 | 23 | urban_med | 64800 | 0.0853080569 |
| 2012 | 21.12 | 0.5 | 24 | urban_high | 10800 | 0.0142180095 |
| 2012 | 21.12 | 0.5 | 41 | dec_forest | 468000 | 0.6161137441 |
| 2012 | 21.12 | 0.5 | 81 | pasture_hay | 65700 | 0.086492891 |
| 2012 | 21.12 | 0.5 | 82 | crop | 54000 | 0.0710900474 |
| 2012 | 26.12 | 0.5 | 21 | urban_open | 281700 | 0.3695395514 |
| 2012 | 26.12 | 0.5 | 22 | urban_low | 57600 | 0.0755608028 |
| 2012 | 26.12 | 0.5 | 41 | dec_forest | 176400 | 0.2314049587 |
| 2012 | 26.12 | 0.5 | 81 | pasture_hay | 242100 | 0.3175914994 |
| 2012 | 26.12 | 0.5 | 82 | crop | 4500 | 0.0059031877 |
| 2012 | 27.12 | 0.5 | 21 | urban_open | 96300 | 0.1260306243 |
| 2012 | 27.12 | 0.5 | 22 | urban_low | 22500 | 0.0294464075 |
| 2012 | 27.12 | 0.5 | 41 | dec_forest | 2700 | 0.0035335689 |
| 2012 | 27.12 | 0.5 | 71 | grass_herb | 12600 | 0.0164899882 |
| 2012 | 27.12 | 0.5 | 82 | crop | 630000 | 0.8244994111 |
| 2012 | 28.12 | 0.5 | 11 | open_water | 4500 | 0.0058139535 |
| 2012 | 28.12 | 0.5 | 21 | urban_open | 77400 | 0.1 |
| 2012 | 28.12 | 0.5 | 22 | urban_low | 25200 | 0.0325581395 |
| 2012 | 28.12 | 0.5 | 41 | dec_forest | 265500 | 0.3430232558 |
| 2012 | 28.12 | 0.5 | 71 | grass_herb | 46800 | 0.0604651163 |
| 2012 | 28.12 | 0.5 | 81 | pasture_hay | 189900 | 0.2453488372 |
| 2012 | 28.12 | 0.5 | 82 | crop | 156600 | 0.2023255814 |
| 2012 | 28.12 | 0.5 | 90 | woody_wetland | 8100 | 0.0104651163 |
| 2012 | 30.12 | 0.5 | 11 | open_water | 8100 | 0.0107015458 |
| 2012 | 30.12 | 0.5 | 21 | urban_open | 411300 | 0.5434007134 |
| 2012 | 30.12 | 0.5 | 22 | urban_low | 206100 | 0.272294887 |
| 2012 | 30.12 | 0.5 | 23 | urban_med | 20700 | 0.0273483948 |
| 2012 | 30.12 | 0.5 | 41 | dec_forest | 53100 | 0.0701545779 |
| 2012 | 30.12 | 0.5 | 71 | grass_herb | 32400 | 0.0428061831 |
| 2012 | 30.12 | 0.5 | 81 | pasture_hay | 25200 | 0.033293698 |
| 2012 | 33.12 | 0.5 | 21 | urban_open | 329400 | 0.4357142857 |
| 2012 | 33.12 | 0.5 | 22 | urban_low | 378900 | 0.5011904762 |
| 2012 | 33.12 | 0.5 | 23 | urban_med | 29700 | 0.0392857143 |
| 2012 | 33.12 | 0.5 | 24 | urban_high | 2700 | 0.0035714286 |
| 2012 | 33.12 | 0.5 | 41 | dec_forest | 15300 | 0.0202380952 |
| 2012 | 36.12 | 0.5 | 21 | urban_open | 24300 | 0.0317647059 |
| 2012 | 36.12 | 0.5 | 22 | urban_low | 32400 | 0.0423529412 |
| 2012 | 36.12 | 0.5 | 23 | urban_med | 5400 | 0.0070588235 |
| 2012 | 36.12 | 0.5 | 41 | dec_forest | 80100 | 0.1047058824 |
| 2012 | 36.12 | 0.5 | 81 | pasture_hay | 337500 | 0.4411764706 |
| 2012 | 36.12 | 0.5 | 82 | crop | 285300 | 0.3729411765 |
| 2012 | 40.12 | 0.5 | 21 | urban_open | 16200 | 0.0213017751 |
| 2012 | 40.12 | 0.5 | 41 | dec_forest | 463500 | 0.6094674556 |
| 2012 | 40.12 | 0.5 | 42 | eg_forest | 3600 | 0.0047337278 |
| 2012 | 40.12 | 0.5 | 81 | pasture_hay | 189000 | 0.2485207101 |
| 2012 | 40.12 | 0.5 | 82 | crop | 88200 | 0.1159763314 |
| 2012 | 41.12 | 0.5 | 21 | urban_open | 225000 | 0.2944640754 |
| 2012 | 41.12 | 0.5 | 22 | urban_low | 346500 | 0.4534746761 |
| 2012 | 41.12 | 0.5 | 23 | urban_med | 189000 | 0.2473498233 |
| 2012 | 41.12 | 0.5 | 24 | urban_high | 3600 | 0.0047114252 |
| 2012 | 42.12 | 0.5 | 21 | urban_open | 80100 | 0.105450237 |
| 2012 | 42.12 | 0.5 | 41 | dec_forest | 640800 | 0.8436018957 |
| 2012 | 42.12 | 0.5 | 42 | eg_forest | 5400 | 0.0071090047 |
| 2012 | 42.12 | 0.5 | 52 | scrub | 14400 | 0.018957346 |
| 2012 | 42.12 | 0.5 | 81 | pasture_hay | 18900 | 0.0248815166 |
| 2012 | 43.12 | 0.5 | 21 | urban_open | 87300 | 0.1145218418 |
| 2012 | 43.12 | 0.5 | 22 | urban_low | 42300 | 0.0554899646 |
| 2012 | 43.12 | 0.5 | 41 | dec_forest | 280800 | 0.3683589138 |
| 2012 | 43.12 | 0.5 | 81 | pasture_hay | 32400 | 0.0425029516 |
| 2012 | 43.12 | 0.5 | 82 | crop | 319500 | 0.4191263282 |
| 2012 | 44.12 | 0.5 | 21 | urban_open | 37800 | 0.0502994012 |
| 2012 | 44.12 | 0.5 | 41 | dec_forest | 630900 | 0.8395209581 |
| 2012 | 44.12 | 0.5 | 52 | scrub | 12600 | 0.0167664671 |
| 2012 | 44.12 | 0.5 | 71 | grass_herb | 9900 | 0.0131736527 |
| 2012 | 44.12 | 0.5 | 82 | crop | 41400 | 0.0550898204 |
| 2012 | 44.12 | 0.5 | 90 | woody_wetland | 18900 | 0.0251497006 |
| 2012 | 45.12 | 0.5 | 21 | urban_open | 127800 | 0.1682464455 |
| 2012 | 45.12 | 0.5 | 22 | urban_low | 424800 | 0.5592417062 |
| 2012 | 45.12 | 0.5 | 23 | urban_med | 171000 | 0.2251184834 |
| 2012 | 45.12 | 0.5 | 24 | urban_high | 36000 | 0.0473933649 |
| 2012 | 47.12 | 0.5 | 21 | urban_open | 19800 | 0.0260047281 |
| 2012 | 47.12 | 0.5 | 22 | urban_low | 655200 | 0.8605200946 |
| 2012 | 47.12 | 0.5 | 23 | urban_med | 72900 | 0.0957446809 |
| 2012 | 47.12 | 0.5 | 24 | urban_high | 13500 | 0.0177304965 |
| 2012 | 50.12 | 0.5 | 11 | open_water | 54900 | 0.0727056019 |
| 2012 | 50.12 | 0.5 | 21 | urban_open | 151200 | 0.200238379 |
| 2012 | 50.12 | 0.5 | 41 | dec_forest | 459900 | 0.6090584029 |
| 2012 | 50.12 | 0.5 | 71 | grass_herb | 6300 | 0.0083432658 |
| 2012 | 50.12 | 0.5 | 81 | pasture_hay | 82800 | 0.1096543504 |
| 2012 | 52.12 | 0.5 | 21 | urban_open | 73800 | 0.0960187354 |
| 2012 | 52.12 | 0.5 | 22 | urban_low | 5400 | 0.0070257611 |
| 2012 | 52.12 | 0.5 | 41 | dec_forest | 240300 | 0.31264637 |
| 2012 | 52.12 | 0.5 | 71 | grass_herb | 4500 | 0.0058548009 |
| 2012 | 52.12 | 0.5 | 81 | pasture_hay | 150300 | 0.1955503513 |
| 2012 | 52.12 | 0.5 | 82 | crop | 288000 | 0.37470726 |
| 2012 | 52.12 | 0.5 | 90 | woody_wetland | 6300 | 0.0081967213 |
| 2012 | 53.12 | 0.5 | 21 | urban_open | 30600 | 0.0400943396 |
| 2012 | 53.12 | 0.5 | 22 | urban_low | 1800 | 0.0023584906 |
| 2012 | 53.12 | 0.5 | 41 | dec_forest | 389700 | 0.5106132075 |
| 2012 | 53.12 | 0.5 | 52 | scrub | 12600 | 0.016509434 |
| 2012 | 53.12 | 0.5 | 71 | grass_herb | 18900 | 0.0247641509 |
| 2012 | 53.12 | 0.5 | 81 | pasture_hay | 309600 | 0.4056603774 |
| 2012 | 54.12 | 0.5 | 21 | urban_open | 38700 | 0.0509478673 |
| 2012 | 54.12 | 0.5 | 22 | urban_low | 18000 | 0.0236966825 |
| 2012 | 54.12 | 0.5 | 41 | dec_forest | 26100 | 0.0343601896 |
| 2012 | 54.12 | 0.5 | 81 | pasture_hay | 246600 | 0.3246445498 |
| 2012 | 54.12 | 0.5 | 82 | crop | 430200 | 0.5663507109 |
| 2012 | 101.12 | 0.5 | 21 | urban_open | 243900 | 0.3195754717 |
| 2012 | 101.12 | 0.5 | 22 | urban_low | 104400 | 0.1367924528 |
| 2012 | 101.12 | 0.5 | 23 | urban_med | 33300 | 0.0436320755 |
| 2012 | 101.12 | 0.5 | 41 | dec_forest | 63900 | 0.0837264151 |
| 2012 | 101.12 | 0.5 | 42 | eg_forest | 21600 | 0.0283018868 |
| 2012 | 101.12 | 0.5 | 81 | pasture_hay | 16200 | 0.0212264151 |
| 2012 | 101.12 | 0.5 | 82 | crop | 279900 | 0.366745283 |
| 2012 | 102.12 | 0.5 | 21 | urban_open | 370800 | 0.4904761905 |
| 2012 | 102.12 | 0.5 | 22 | urban_low | 361800 | 0.4785714286 |
| 2012 | 102.12 | 0.5 | 23 | urban_med | 9900 | 0.0130952381 |
| 2012 | 102.12 | 0.5 | 24 | urban_high | 13500 | 0.0178571429 |
| 2012 | 104.12 | 0.5 | 21 | urban_open | 29700 | 0.038961039 |
| 2012 | 104.12 | 0.5 | 82 | crop | 732600 | 0.961038961 |
| 2012 | 107.12 | 0.5 | 21 | urban_open | 68400 | 0.0896226415 |
| 2012 | 107.12 | 0.5 | 22 | urban_low | 6300 | 0.008254717 |
| 2012 | 107.12 | 0.5 | 41 | dec_forest | 126000 | 0.1650943396 |
| 2012 | 107.12 | 0.5 | 43 | mix_forest | 27900 | 0.0365566038 |
| 2012 | 107.12 | 0.5 | 81 | pasture_hay | 156600 | 0.2051886792 |
| 2012 | 107.12 | 0.5 | 82 | crop | 378000 | 0.4952830189 |
| 2012 | 108.12 | 0.5 | 21 | urban_open | 129600 | 0.1696113074 |
| 2012 | 108.12 | 0.5 | 22 | urban_low | 421200 | 0.5512367491 |
| 2012 | 108.12 | 0.5 | 23 | urban_med | 154800 | 0.2025912839 |
| 2012 | 108.12 | 0.5 | 24 | urban_high | 58500 | 0.0765606596 |
| 2012 | 111.12 | 0.5 | 11 | open_water | 9000 | 0.0118764846 |
| 2012 | 111.12 | 0.5 | 21 | urban_open | 414000 | 0.5463182898 |
| 2012 | 111.12 | 0.5 | 22 | urban_low | 153900 | 0.203087886 |
| 2012 | 111.12 | 0.5 | 23 | urban_med | 32400 | 0.0427553444 |
| 2012 | 111.12 | 0.5 | 41 | dec_forest | 93600 | 0.1235154394 |
| 2012 | 111.12 | 0.5 | 71 | grass_herb | 32400 | 0.0427553444 |
| 2012 | 111.12 | 0.5 | 81 | pasture_hay | 16200 | 0.0213776722 |
| 2012 | 111.12 | 0.5 | 90 | woody_wetland | 6300 | 0.0083135392 |
| 2012 | 112.12 | 0.5 | 11 | open_water | 18000 | 0.0236966825 |
| 2012 | 112.12 | 0.5 | 21 | urban_open | 338400 | 0.4454976303 |
| 2012 | 112.12 | 0.5 | 22 | urban_low | 393300 | 0.5177725118 |
| 2012 | 112.12 | 0.5 | 23 | urban_med | 9900 | 0.0130331754 |
| 2012 | 113.12 | 0.5 | 21 | urban_open | 37800 | 0.0495283019 |
| 2012 | 113.12 | 0.5 | 22 | urban_low | 438300 | 0.5742924528 |
| 2012 | 113.12 | 0.5 | 23 | urban_med | 205200 | 0.2688679245 |
| 2012 | 113.12 | 0.5 | 24 | urban_high | 79200 | 0.1037735849 |
| 2012 | 113.12 | 0.5 | 41 | dec_forest | 2700 | 0.0035377358 |
| 2012 | 115.12 | 0.5 | 21 | urban_open | 184500 | 0.2434679335 |
| 2012 | 115.12 | 0.5 | 22 | urban_low | 244800 | 0.32304038 |
| 2012 | 115.12 | 0.5 | 23 | urban_med | 47700 | 0.0629453682 |
| 2012 | 115.12 | 0.5 | 24 | urban_high | 20700 | 0.0273159145 |
| 2012 | 115.12 | 0.5 | 41 | dec_forest | 240300 | 0.3171021378 |
| 2012 | 115.12 | 0.5 | 71 | grass_herb | 19800 | 0.026128266 |
| 2012 | 9.12 | 1 | 21 | urban_open | 1016100 | 0.328962704 |
| 2012 | 9.12 | 1 | 22 | urban_low | 829800 | 0.2686480186 |
| 2012 | 9.12 | 1 | 23 | urban_med | 121500 | 0.0393356643 |
| 2012 | 9.12 | 1 | 24 | urban_high | 28800 | 0.0093240093 |
| 2012 | 9.12 | 1 | 31 | barren | 58500 | 0.0189393939 |
| 2012 | 9.12 | 1 | 41 | dec_forest | 512100 | 0.1657925408 |
| 2012 | 9.12 | 1 | 42 | eg_forest | 20700 | 0.0067016317 |
| 2012 | 9.12 | 1 | 43 | mix_forest | 6300 | 0.002039627 |
| 2012 | 9.12 | 1 | 71 | grass_herb | 46800 | 0.0151515152 |
| 2012 | 9.12 | 1 | 81 | pasture_hay | 373500 | 0.1209207459 |
| 2012 | 9.12 | 1 | 90 | woody_wetland | 74700 | 0.0241841492 |
| 2012 | 11.12 | 1 | 11 | open_water | 11700 | 0.0037956204 |
| 2012 | 11.12 | 1 | 21 | urban_open | 68400 | 0.022189781 |
| 2012 | 11.12 | 1 | 22 | urban_low | 87300 | 0.0283211679 |
| 2012 | 11.12 | 1 | 23 | urban_med | 52200 | 0.0169343066 |
| 2012 | 11.12 | 1 | 41 | dec_forest | 864900 | 0.2805839416 |
| 2012 | 11.12 | 1 | 42 | eg_forest | 29700 | 0.0096350365 |
| 2012 | 11.12 | 1 | 52 | scrub | 70200 | 0.0227737226 |
| 2012 | 11.12 | 1 | 81 | pasture_hay | 178200 | 0.057810219 |
| 2012 | 11.12 | 1 | 82 | crop | 1719900 | 0.5579562044 |
| 2012 | 12.12 | 1 | 21 | urban_open | 1188000 | 0.384951881 |
| 2012 | 12.12 | 1 | 22 | urban_low | 1314000 | 0.4257801108 |
| 2012 | 12.12 | 1 | 23 | urban_med | 376200 | 0.121901429 |
| 2012 | 12.12 | 1 | 24 | urban_high | 187200 | 0.0606590843 |
| 2012 | 12.12 | 1 | 41 | dec_forest | 20700 | 0.0067074949 |
| 2012 | 17.12 | 1 | 21 | urban_open | 1286100 | 0.4172262774 |
| 2012 | 17.12 | 1 | 22 | urban_low | 940500 | 0.3051094891 |
| 2012 | 17.12 | 1 | 23 | urban_med | 172800 | 0.0560583942 |
| 2012 | 17.12 | 1 | 24 | urban_high | 152100 | 0.0493430657 |
| 2012 | 17.12 | 1 | 31 | barren | 38700 | 0.0125547445 |
| 2012 | 17.12 | 1 | 41 | dec_forest | 427500 | 0.1386861314 |
| 2012 | 17.12 | 1 | 71 | grass_herb | 9000 | 0.002919708 |
| 2012 | 17.12 | 1 | 81 | pasture_hay | 43200 | 0.0140145985 |
| 2012 | 17.12 | 1 | 90 | woody_wetland | 12600 | 0.0040875912 |
| 2012 | 20.12 | 1 | 21 | urban_open | 174600 | 0.0566423358 |
| 2012 | 20.12 | 1 | 41 | dec_forest | 207900 | 0.0674452555 |
| 2012 | 20.12 | 1 | 81 | pasture_hay | 442800 | 0.143649635 |
| 2012 | 20.12 | 1 | 82 | crop | 2257200 | 0.7322627737 |
| 2012 | 21.12 | 1 | 11 | open_water | 13500 | 0.0043872477 |
| 2012 | 21.12 | 1 | 21 | urban_open | 465300 | 0.1512138052 |
| 2012 | 21.12 | 1 | 22 | urban_low | 235800 | 0.0766305937 |
| 2012 | 21.12 | 1 | 23 | urban_med | 303300 | 0.0985668324 |
| 2012 | 21.12 | 1 | 24 | urban_high | 72900 | 0.0236911378 |
| 2012 | 21.12 | 1 | 41 | dec_forest | 1698300 | 0.5519157648 |
| 2012 | 21.12 | 1 | 42 | eg_forest | 15300 | 0.0049722141 |
| 2012 | 21.12 | 1 | 52 | scrub | 11700 | 0.0038022814 |
| 2012 | 21.12 | 1 | 71 | grass_herb | 7200 | 0.0023398655 |
| 2012 | 21.12 | 1 | 81 | pasture_hay | 165600 | 0.0538169055 |
| 2012 | 21.12 | 1 | 82 | crop | 88200 | 0.0286633519 |
| 2012 | 26.12 | 1 | 21 | urban_open | 655200 | 0.2131147541 |
| 2012 | 26.12 | 1 | 22 | urban_low | 198000 | 0.0644028103 |
| 2012 | 26.12 | 1 | 23 | urban_med | 4500 | 0.0014637002 |
| 2012 | 26.12 | 1 | 41 | dec_forest | 1179900 | 0.3837822014 |
| 2012 | 26.12 | 1 | 42 | eg_forest | 16200 | 0.0052693208 |
| 2012 | 26.12 | 1 | 71 | grass_herb | 76500 | 0.024882904 |
| 2012 | 26.12 | 1 | 81 | pasture_hay | 900000 | 0.2927400468 |
| 2012 | 26.12 | 1 | 82 | crop | 44100 | 0.0143442623 |
| 2012 | 27.12 | 1 | 21 | urban_open | 187200 | 0.060729927 |
| 2012 | 27.12 | 1 | 22 | urban_low | 37800 | 0.0122627737 |
| 2012 | 27.12 | 1 | 41 | dec_forest | 247500 | 0.0802919708 |
| 2012 | 27.12 | 1 | 42 | eg_forest | 14400 | 0.0046715328 |
| 2012 | 27.12 | 1 | 71 | grass_herb | 73800 | 0.0239416058 |
| 2012 | 27.12 | 1 | 82 | crop | 2519100 | 0.8172262774 |
| 2012 | 27.12 | 1 | 90 | woody_wetland | 2700 | 0.0008759124 |
| 2012 | 28.12 | 1 | 11 | open_water | 12600 | 0.0040947646 |
| 2012 | 28.12 | 1 | 21 | urban_open | 279000 | 0.0906697865 |
| 2012 | 28.12 | 1 | 22 | urban_low | 138600 | 0.0450424101 |
| 2012 | 28.12 | 1 | 23 | urban_med | 2700 | 0.0008774495 |
| 2012 | 28.12 | 1 | 41 | dec_forest | 1379700 | 0.4483767183 |
| 2012 | 28.12 | 1 | 42 | eg_forest | 12600 | 0.0040947646 |
| 2012 | 28.12 | 1 | 71 | grass_herb | 225900 | 0.0734132787 |
| 2012 | 28.12 | 1 | 81 | pasture_hay | 643500 | 0.2091254753 |
| 2012 | 28.12 | 1 | 82 | crop | 369900 | 0.1202105879 |
| 2012 | 28.12 | 1 | 90 | woody_wetland | 12600 | 0.0040947646 |
| 2012 | 30.12 | 1 | 11 | open_water | 12600 | 0.0040923707 |
| 2012 | 30.12 | 1 | 21 | urban_open | 1098900 | 0.3569131833 |
| 2012 | 30.12 | 1 | 22 | urban_low | 918900 | 0.2984507454 |
| 2012 | 30.12 | 1 | 23 | urban_med | 193500 | 0.0628471207 |
| 2012 | 30.12 | 1 | 24 | urban_high | 15300 | 0.0049693072 |
| 2012 | 30.12 | 1 | 41 | dec_forest | 511200 | 0.1660333236 |
| 2012 | 30.12 | 1 | 52 | scrub | 9900 | 0.0032154341 |
| 2012 | 30.12 | 1 | 71 | grass_herb | 83700 | 0.0271850336 |
| 2012 | 30.12 | 1 | 81 | pasture_hay | 200700 | 0.0651856182 |
| 2012 | 30.12 | 1 | 82 | crop | 34200 | 0.0111078632 |
| 2012 | 33.12 | 1 | 11 | open_water | 10800 | 0.0035200939 |
| 2012 | 33.12 | 1 | 21 | urban_open | 1206000 | 0.3930771487 |
| 2012 | 33.12 | 1 | 22 | urban_low | 1300500 | 0.4238779701 |
| 2012 | 33.12 | 1 | 23 | urban_med | 70200 | 0.0228806101 |
| 2012 | 33.12 | 1 | 24 | urban_high | 5400 | 0.0017600469 |
| 2012 | 33.12 | 1 | 41 | dec_forest | 186300 | 0.0607216192 |
| 2012 | 33.12 | 1 | 71 | grass_herb | 22500 | 0.0073335289 |
| 2012 | 33.12 | 1 | 81 | pasture_hay | 47700 | 0.0155470813 |
| 2012 | 33.12 | 1 | 82 | crop | 218700 | 0.0712819009 |
| 2012 | 36.12 | 1 | 21 | urban_open | 170100 | 0.0553440703 |
| 2012 | 36.12 | 1 | 22 | urban_low | 81000 | 0.0263543192 |
| 2012 | 36.12 | 1 | 23 | urban_med | 5400 | 0.0017569546 |
| 2012 | 36.12 | 1 | 41 | dec_forest | 442800 | 0.1440702782 |
| 2012 | 36.12 | 1 | 43 | mix_forest | 12600 | 0.0040995608 |
| 2012 | 36.12 | 1 | 81 | pasture_hay | 974700 | 0.3171303075 |
| 2012 | 36.12 | 1 | 82 | crop | 1386900 | 0.4512445095 |
| 2012 | 40.12 | 1 | 21 | urban_open | 147600 | 0.0479672419 |
| 2012 | 40.12 | 1 | 41 | dec_forest | 1691100 | 0.5495758994 |
| 2012 | 40.12 | 1 | 42 | eg_forest | 26100 | 0.0084820123 |
| 2012 | 40.12 | 1 | 71 | grass_herb | 26100 | 0.0084820123 |
| 2012 | 40.12 | 1 | 81 | pasture_hay | 645300 | 0.2097104416 |
| 2012 | 40.12 | 1 | 82 | crop | 540900 | 0.1757823925 |
| 2012 | 41.12 | 1 | 21 | urban_open | 320400 | 0.1039112668 |
| 2012 | 41.12 | 1 | 22 | urban_low | 1331100 | 0.4316987741 |
| 2012 | 41.12 | 1 | 23 | urban_med | 1211400 | 0.3928779918 |
| 2012 | 41.12 | 1 | 24 | urban_high | 220500 | 0.0715119673 |
| 2012 | 42.12 | 1 | 21 | urban_open | 215100 | 0.0698830409 |
| 2012 | 42.12 | 1 | 41 | dec_forest | 2714400 | 0.881871345 |
| 2012 | 42.12 | 1 | 42 | eg_forest | 61200 | 0.0198830409 |
| 2012 | 42.12 | 1 | 52 | scrub | 25200 | 0.0081871345 |
| 2012 | 42.12 | 1 | 81 | pasture_hay | 62100 | 0.0201754386 |
| 2012 | 43.12 | 1 | 21 | urban_open | 237600 | 0.0769230769 |
| 2012 | 43.12 | 1 | 22 | urban_low | 94500 | 0.0305944056 |
| 2012 | 43.12 | 1 | 41 | dec_forest | 794700 | 0.2572843823 |
| 2012 | 43.12 | 1 | 42 | eg_forest | 8100 | 0.0026223776 |
| 2012 | 43.12 | 1 | 81 | pasture_hay | 198900 | 0.0643939394 |
| 2012 | 43.12 | 1 | 82 | crop | 1733400 | 0.5611888112 |
| 2012 | 43.12 | 1 | 90 | woody_wetland | 21600 | 0.006993007 |
| 2012 | 44.12 | 1 | 21 | urban_open | 101700 | 0.0330023364 |
| 2012 | 44.12 | 1 | 22 | urban_low | 17100 | 0.0055490654 |
| 2012 | 44.12 | 1 | 41 | dec_forest | 1911600 | 0.6203271028 |
| 2012 | 44.12 | 1 | 42 | eg_forest | 9000 | 0.0029205607 |
| 2012 | 44.12 | 1 | 52 | scrub | 138600 | 0.0449766355 |
| 2012 | 44.12 | 1 | 71 | grass_herb | 195300 | 0.0633761682 |
| 2012 | 44.12 | 1 | 81 | pasture_hay | 90000 | 0.0292056075 |
| 2012 | 44.12 | 1 | 82 | crop | 560700 | 0.1819509346 |
| 2012 | 44.12 | 1 | 90 | woody_wetland | 57600 | 0.0186915888 |
| 2012 | 45.12 | 1 | 21 | urban_open | 654300 | 0.2129466901 |
| 2012 | 45.12 | 1 | 22 | urban_low | 1451700 | 0.4724663152 |
| 2012 | 45.12 | 1 | 23 | urban_med | 630900 | 0.20533099 |
| 2012 | 45.12 | 1 | 24 | urban_high | 171000 | 0.0556531927 |
| 2012 | 45.12 | 1 | 71 | grass_herb | 33300 | 0.010837727 |
| 2012 | 45.12 | 1 | 82 | crop | 131400 | 0.0427650849 |
| 2012 | 47.12 | 1 | 21 | urban_open | 486900 | 0.1585115734 |
| 2012 | 47.12 | 1 | 22 | urban_low | 1693800 | 0.5514210372 |
| 2012 | 47.12 | 1 | 23 | urban_med | 618300 | 0.2012891884 |
| 2012 | 47.12 | 1 | 24 | urban_high | 272700 | 0.088778201 |
| 2012 | 50.12 | 1 | 11 | open_water | 734400 | 0.2381091334 |
| 2012 | 50.12 | 1 | 21 | urban_open | 385200 | 0.1248905748 |
| 2012 | 50.12 | 1 | 22 | urban_low | 66600 | 0.0215932302 |
| 2012 | 50.12 | 1 | 23 | urban_med | 52200 | 0.0169244237 |
| 2012 | 50.12 | 1 | 24 | urban_high | 27000 | 0.0087540123 |
| 2012 | 50.12 | 1 | 41 | dec_forest | 1549800 | 0.5024803035 |
| 2012 | 50.12 | 1 | 71 | grass_herb | 40500 | 0.0131310184 |
| 2012 | 50.12 | 1 | 81 | pasture_hay | 228600 | 0.0741173038 |
| 2012 | 52.12 | 1 | 21 | urban_open | 186300 | 0.0607572645 |
| 2012 | 52.12 | 1 | 22 | urban_low | 5400 | 0.0017610801 |
| 2012 | 52.12 | 1 | 41 | dec_forest | 1329300 | 0.4335192251 |
| 2012 | 52.12 | 1 | 71 | grass_herb | 5400 | 0.0017610801 |
| 2012 | 52.12 | 1 | 81 | pasture_hay | 577800 | 0.1884355738 |
| 2012 | 52.12 | 1 | 82 | crop | 954000 | 0.3111241561 |
| 2012 | 52.12 | 1 | 90 | woody_wetland | 8100 | 0.0026416202 |
| 2012 | 53.12 | 1 | 21 | urban_open | 162000 | 0.052801408 |
| 2012 | 53.12 | 1 | 22 | urban_low | 36900 | 0.0120269874 |
| 2012 | 53.12 | 1 | 23 | urban_med | 5400 | 0.0017600469 |
| 2012 | 53.12 | 1 | 41 | dec_forest | 1295100 | 0.4221179231 |
| 2012 | 53.12 | 1 | 42 | eg_forest | 42300 | 0.0137870343 |
| 2012 | 53.12 | 1 | 52 | scrub | 31500 | 0.0102669405 |
| 2012 | 53.12 | 1 | 71 | grass_herb | 82800 | 0.0269873863 |
| 2012 | 53.12 | 1 | 81 | pasture_hay | 1281600 | 0.4177178058 |
| 2012 | 53.12 | 1 | 82 | crop | 130500 | 0.0425344676 |
| 2012 | 54.12 | 1 | 21 | urban_open | 254700 | 0.082748538 |
| 2012 | 54.12 | 1 | 22 | urban_low | 180000 | 0.0584795322 |
| 2012 | 54.12 | 1 | 23 | urban_med | 2700 | 0.000877193 |
| 2012 | 54.12 | 1 | 41 | dec_forest | 225000 | 0.0730994152 |
| 2012 | 54.12 | 1 | 71 | grass_herb | 30600 | 0.0099415205 |
| 2012 | 54.12 | 1 | 81 | pasture_hay | 590400 | 0.1918128655 |
| 2012 | 54.12 | 1 | 82 | crop | 1794600 | 0.5830409357 |
| 2012 | 101.12 | 1 | 21 | urban_open | 978300 | 0.319236417 |
| 2012 | 101.12 | 1 | 22 | urban_low | 408600 | 0.1333333333 |
| 2012 | 101.12 | 1 | 23 | urban_med | 110700 | 0.036123348 |
| 2012 | 101.12 | 1 | 24 | urban_high | 15300 | 0.0049926579 |
| 2012 | 101.12 | 1 | 41 | dec_forest | 138600 | 0.0452276065 |
| 2012 | 101.12 | 1 | 42 | eg_forest | 64800 | 0.0211453744 |
| 2012 | 101.12 | 1 | 71 | grass_herb | 19800 | 0.0064610866 |
| 2012 | 101.12 | 1 | 81 | pasture_hay | 304200 | 0.0992657856 |
| 2012 | 101.12 | 1 | 82 | crop | 1024200 | 0.3342143906 |
| 2012 | 102.12 | 1 | 11 | open_water | 31500 | 0.0102369114 |
| 2012 | 102.12 | 1 | 21 | urban_open | 1169100 | 0.3799356537 |
| 2012 | 102.12 | 1 | 22 | urban_low | 1574100 | 0.5115530857 |
| 2012 | 102.12 | 1 | 23 | urban_med | 176400 | 0.0573267037 |
| 2012 | 102.12 | 1 | 24 | urban_high | 54000 | 0.0175489909 |
| 2012 | 102.12 | 1 | 41 | dec_forest | 67500 | 0.0219362387 |
| 2012 | 102.12 | 1 | 71 | grass_herb | 4500 | 0.0014624159 |
| 2012 | 104.12 | 1 | 21 | urban_open | 108000 | 0.0350979819 |
| 2012 | 104.12 | 1 | 22 | urban_low | 9900 | 0.003217315 |
| 2012 | 104.12 | 1 | 71 | grass_herb | 22500 | 0.0073120796 |
| 2012 | 104.12 | 1 | 82 | crop | 2936700 | 0.9543726236 |
| 2012 | 107.12 | 1 | 21 | urban_open | 175500 | 0.0568016312 |
| 2012 | 107.12 | 1 | 22 | urban_low | 27900 | 0.0090300029 |
| 2012 | 107.12 | 1 | 41 | dec_forest | 422100 | 0.1366152054 |
| 2012 | 107.12 | 1 | 43 | mix_forest | 115200 | 0.0372851733 |
| 2012 | 107.12 | 1 | 81 | pasture_hay | 247500 | 0.0801048645 |
| 2012 | 107.12 | 1 | 82 | crop | 2101500 | 0.6801631226 |
| 2012 | 108.12 | 1 | 21 | urban_open | 752400 | 0.245017585 |
| 2012 | 108.12 | 1 | 22 | urban_low | 1539900 | 0.5014654162 |
| 2012 | 108.12 | 1 | 23 | urban_med | 529200 | 0.1723329426 |
| 2012 | 108.12 | 1 | 24 | urban_high | 206100 | 0.067116061 |
| 2012 | 108.12 | 1 | 41 | dec_forest | 43200 | 0.0140679953 |
| 2012 | 111.12 | 1 | 11 | open_water | 10800 | 0.0035190616 |
| 2012 | 111.12 | 1 | 21 | urban_open | 1137600 | 0.3706744868 |
| 2012 | 111.12 | 1 | 22 | urban_low | 1207800 | 0.3935483871 |
| 2012 | 111.12 | 1 | 23 | urban_med | 247500 | 0.0806451613 |
| 2012 | 111.12 | 1 | 24 | urban_high | 15300 | 0.0049853372 |
| 2012 | 111.12 | 1 | 41 | dec_forest | 284400 | 0.0926686217 |
| 2012 | 111.12 | 1 | 71 | grass_herb | 78300 | 0.0255131965 |
| 2012 | 111.12 | 1 | 81 | pasture_hay | 64800 | 0.0211143695 |
| 2012 | 111.12 | 1 | 90 | woody_wetland | 22500 | 0.0073313783 |
| 2012 | 112.12 | 1 | 11 | open_water | 183600 | 0.0597714621 |
| 2012 | 112.12 | 1 | 21 | urban_open | 1174500 | 0.3823615587 |
| 2012 | 112.12 | 1 | 22 | urban_low | 1433700 | 0.4667447993 |
| 2012 | 112.12 | 1 | 23 | urban_med | 54000 | 0.0175798418 |
| 2012 | 112.12 | 1 | 41 | dec_forest | 153900 | 0.0501025491 |
| 2012 | 112.12 | 1 | 42 | eg_forest | 12600 | 0.0041019631 |
| 2012 | 112.12 | 1 | 43 | mix_forest | 11700 | 0.0038089657 |
| 2012 | 112.12 | 1 | 71 | grass_herb | 9000 | 0.0029299736 |
| 2012 | 112.12 | 1 | 81 | pasture_hay | 32400 | 0.0105479051 |
| 2012 | 112.12 | 1 | 95 | em_herb_wetland | 6300 | 0.0020509815 |
| 2012 | 113.12 | 1 | 21 | urban_open | 100800 | 0.0326911851 |
| 2012 | 113.12 | 1 | 22 | urban_low | 1800900 | 0.5840630473 |
| 2012 | 113.12 | 1 | 23 | urban_med | 783900 | 0.2542323409 |
| 2012 | 113.12 | 1 | 24 | urban_high | 360000 | 0.1167542323 |
| 2012 | 113.12 | 1 | 41 | dec_forest | 37800 | 0.0122591944 |
| 2012 | 115.12 | 1 | 11 | open_water | 4500 | 0.0014658458 |
| 2012 | 115.12 | 1 | 21 | urban_open | 686700 | 0.223688068 |
| 2012 | 115.12 | 1 | 22 | urban_low | 1083600 | 0.352975667 |
| 2012 | 115.12 | 1 | 23 | urban_med | 245700 | 0.0800351803 |
| 2012 | 115.12 | 1 | 24 | urban_high | 100800 | 0.0328349458 |
| 2012 | 115.12 | 1 | 41 | dec_forest | 787500 | 0.2565230138 |
| 2012 | 115.12 | 1 | 71 | grass_herb | 56700 | 0.018469657 |
| 2012 | 115.12 | 1 | 81 | pasture_hay | 99900 | 0.0325417766 |
| 2012 | 115.12 | 1 | 90 | woody_wetland | 4500 | 0.0014658458 |
| 2012 | 9.12 | 2 | 11 | open_water | 4500 | 0.0003647771 |
| 2012 | 9.12 | 2 | 21 | urban_open | 3773700 | 0.3059020938 |
| 2012 | 9.12 | 2 | 22 | urban_low | 3406500 | 0.2761362807 |
| 2012 | 9.12 | 2 | 23 | urban_med | 1125000 | 0.0911942803 |
| 2012 | 9.12 | 2 | 24 | urban_high | 330300 | 0.0267746407 |
| 2012 | 9.12 | 2 | 31 | barren | 99000 | 0.0080250967 |
| 2012 | 9.12 | 2 | 41 | dec_forest | 2096100 | 0.169913183 |
| 2012 | 9.12 | 2 | 42 | eg_forest | 28800 | 0.0023345736 |
| 2012 | 9.12 | 2 | 43 | mix_forest | 6300 | 0.000510688 |
| 2012 | 9.12 | 2 | 52 | scrub | 7200 | 0.0005836434 |
| 2012 | 9.12 | 2 | 71 | grass_herb | 240300 | 0.0194790983 |
| 2012 | 9.12 | 2 | 81 | pasture_hay | 819000 | 0.0663894361 |
| 2012 | 9.12 | 2 | 90 | woody_wetland | 399600 | 0.0323922084 |
| 2012 | 11.12 | 2 | 11 | open_water | 130500 | 0.0105662027 |
| 2012 | 11.12 | 2 | 21 | urban_open | 276300 | 0.0223712016 |
| 2012 | 11.12 | 2 | 22 | urban_low | 279900 | 0.0226626831 |
| 2012 | 11.12 | 2 | 23 | urban_med | 109800 | 0.0088901844 |
| 2012 | 11.12 | 2 | 41 | dec_forest | 2396700 | 0.1940537783 |
| 2012 | 11.12 | 2 | 42 | eg_forest | 36900 | 0.0029876849 |
| 2012 | 11.12 | 2 | 52 | scrub | 82800 | 0.0067040735 |
| 2012 | 11.12 | 2 | 71 | grass_herb | 19800 | 0.001603148 |
| 2012 | 11.12 | 2 | 81 | pasture_hay | 1563300 | 0.1265758216 |
| 2012 | 11.12 | 2 | 82 | crop | 7454700 | 0.6035852219 |
| 2012 | 12.12 | 2 | 11 | open_water | 3600 | 0.0002918856 |
| 2012 | 12.12 | 2 | 21 | urban_open | 3594600 | 0.2914477525 |
| 2012 | 12.12 | 2 | 22 | urban_low | 4871700 | 0.3949941623 |
| 2012 | 12.12 | 2 | 23 | urban_med | 2181600 | 0.176882662 |
| 2012 | 12.12 | 2 | 24 | urban_high | 1443600 | 0.1170461179 |
| 2012 | 12.12 | 2 | 41 | dec_forest | 179100 | 0.0145213076 |
| 2012 | 12.12 | 2 | 42 | eg_forest | 5400 | 0.0004378284 |
| 2012 | 12.12 | 2 | 71 | grass_herb | 50400 | 0.0040863981 |
| 2012 | 12.12 | 2 | 81 | pasture_hay | 3600 | 0.0002918856 |
| 2012 | 17.12 | 2 | 11 | open_water | 177300 | 0.0143816616 |
| 2012 | 17.12 | 2 | 21 | urban_open | 4775400 | 0.3873558184 |
| 2012 | 17.12 | 2 | 22 | urban_low | 3183300 | 0.2582128778 |
| 2012 | 17.12 | 2 | 23 | urban_med | 329400 | 0.0267192291 |
| 2012 | 17.12 | 2 | 24 | urban_high | 203400 | 0.0164987589 |
| 2012 | 17.12 | 2 | 31 | barren | 65700 | 0.0053292451 |
| 2012 | 17.12 | 2 | 41 | dec_forest | 1938600 | 0.1572492335 |
| 2012 | 17.12 | 2 | 42 | eg_forest | 43200 | 0.0035041612 |
| 2012 | 17.12 | 2 | 43 | mix_forest | 4500 | 0.0003650168 |
| 2012 | 17.12 | 2 | 52 | scrub | 4500 | 0.0003650168 |
| 2012 | 17.12 | 2 | 71 | grass_herb | 161100 | 0.0130676011 |
| 2012 | 17.12 | 2 | 81 | pasture_hay | 734400 | 0.0595707403 |
| 2012 | 17.12 | 2 | 82 | crop | 569700 | 0.0462111257 |
| 2012 | 17.12 | 2 | 90 | woody_wetland | 117900 | 0.0095634399 |
| 2012 | 17.12 | 2 | 95 | em_herb_wetland | 19800 | 0.0016060739 |
| 2012 | 20.12 | 2 | 21 | urban_open | 612000 | 0.0495446266 |
| 2012 | 20.12 | 2 | 22 | urban_low | 125100 | 0.0101275046 |
| 2012 | 20.12 | 2 | 23 | urban_med | 5400 | 0.0004371585 |
| 2012 | 20.12 | 2 | 41 | dec_forest | 957600 | 0.0775227687 |
| 2012 | 20.12 | 2 | 81 | pasture_hay | 1811700 | 0.1466666667 |
| 2012 | 20.12 | 2 | 82 | crop | 8834400 | 0.7151912568 |
| 2012 | 20.12 | 2 | 90 | woody_wetland | 6300 | 0.0005100182 |
| 2012 | 21.12 | 2 | 11 | open_water | 13500 | 0.0010928166 |
| 2012 | 21.12 | 2 | 21 | urban_open | 3865500 | 0.3129098062 |
| 2012 | 21.12 | 2 | 22 | urban_low | 1405800 | 0.1137986303 |
| 2012 | 21.12 | 2 | 23 | urban_med | 700200 | 0.0566807519 |
| 2012 | 21.12 | 2 | 24 | urban_high | 153000 | 0.0123852543 |
| 2012 | 21.12 | 2 | 41 | dec_forest | 5229900 | 0.4233571324 |
| 2012 | 21.12 | 2 | 42 | eg_forest | 183600 | 0.0148623051 |
| 2012 | 21.12 | 2 | 43 | mix_forest | 6300 | 0.0005099811 |
| 2012 | 21.12 | 2 | 52 | scrub | 31500 | 0.0025499053 |
| 2012 | 21.12 | 2 | 71 | grass_herb | 88200 | 0.0071397348 |
| 2012 | 21.12 | 2 | 81 | pasture_hay | 480600 | 0.0389042693 |
| 2012 | 21.12 | 2 | 82 | crop | 195300 | 0.0158094128 |
| 2012 | 26.12 | 2 | 11 | open_water | 5400 | 0.0004370311 |
| 2012 | 26.12 | 2 | 21 | urban_open | 1562400 | 0.1264476655 |
| 2012 | 26.12 | 2 | 22 | urban_low | 497700 | 0.0402796999 |
| 2012 | 26.12 | 2 | 23 | urban_med | 61200 | 0.0049530192 |
| 2012 | 26.12 | 2 | 24 | urban_high | 10800 | 0.0008740622 |
| 2012 | 26.12 | 2 | 31 | barren | 70200 | 0.0056814043 |
| 2012 | 26.12 | 2 | 41 | dec_forest | 5799600 | 0.4693714036 |
| 2012 | 26.12 | 2 | 42 | eg_forest | 114300 | 0.0092504917 |
| 2012 | 26.12 | 2 | 52 | scrub | 19800 | 0.0016024474 |
| 2012 | 26.12 | 2 | 71 | grass_herb | 122400 | 0.0099060383 |
| 2012 | 26.12 | 2 | 81 | pasture_hay | 3780000 | 0.3059217714 |
| 2012 | 26.12 | 2 | 82 | crop | 307800 | 0.0249107728 |
| 2012 | 26.12 | 2 | 90 | woody_wetland | 4500 | 0.0003641926 |
| 2012 | 27.12 | 2 | 21 | urban_open | 657900 | 0.0532410779 |
| 2012 | 27.12 | 2 | 22 | urban_low | 115200 | 0.0093226511 |
| 2012 | 27.12 | 2 | 23 | urban_med | 10800 | 0.0008739985 |
| 2012 | 27.12 | 2 | 41 | dec_forest | 1295100 | 0.104806992 |
| 2012 | 27.12 | 2 | 42 | eg_forest | 61200 | 0.0049526584 |
| 2012 | 27.12 | 2 | 71 | grass_herb | 381600 | 0.0308812819 |
| 2012 | 27.12 | 2 | 81 | pasture_hay | 516600 | 0.0418062637 |
| 2012 | 27.12 | 2 | 82 | crop | 9303300 | 0.7528769119 |
| 2012 | 27.12 | 2 | 90 | woody_wetland | 15300 | 0.0012381646 |
| 2012 | 28.12 | 2 | 11 | open_water | 65700 | 0.0053137283 |
| 2012 | 28.12 | 2 | 21 | urban_open | 1625400 | 0.1314601834 |
| 2012 | 28.12 | 2 | 22 | urban_low | 911700 | 0.0737370796 |
| 2012 | 28.12 | 2 | 23 | urban_med | 212400 | 0.0171786286 |
| 2012 | 28.12 | 2 | 24 | urban_high | 63900 | 0.0051681467 |
| 2012 | 28.12 | 2 | 31 | barren | 18900 | 0.0015286068 |
| 2012 | 28.12 | 2 | 41 | dec_forest | 4509900 | 0.364754695 |
| 2012 | 28.12 | 2 | 42 | eg_forest | 67500 | 0.0054593099 |
| 2012 | 28.12 | 2 | 71 | grass_herb | 657900 | 0.0532100742 |
| 2012 | 28.12 | 2 | 81 | pasture_hay | 1429200 | 0.1155917892 |
| 2012 | 28.12 | 2 | 82 | crop | 2613600 | 0.211384481 |
| 2012 | 28.12 | 2 | 90 | woody_wetland | 182700 | 0.0147765322 |
| 2012 | 28.12 | 2 | 95 | em_herb_wetland | 5400 | 0.0004367448 |
| 2012 | 30.12 | 2 | 11 | open_water | 22500 | 0.0018175209 |
| 2012 | 30.12 | 2 | 21 | urban_open | 2852100 | 0.2303889495 |
| 2012 | 30.12 | 2 | 22 | urban_low | 3800700 | 0.3070156307 |
| 2012 | 30.12 | 2 | 23 | urban_med | 1334700 | 0.1078153399 |
| 2012 | 30.12 | 2 | 24 | urban_high | 259200 | 0.0209378408 |
| 2012 | 30.12 | 2 | 41 | dec_forest | 2289600 | 0.1849509269 |
| 2012 | 30.12 | 2 | 42 | eg_forest | 73800 | 0.0059614686 |
| 2012 | 30.12 | 2 | 52 | scrub | 69300 | 0.0055979644 |
| 2012 | 30.12 | 2 | 71 | grass_herb | 123300 | 0.0099600145 |
| 2012 | 30.12 | 2 | 81 | pasture_hay | 658800 | 0.053217012 |
| 2012 | 30.12 | 2 | 82 | crop | 859500 | 0.0694292984 |
| 2012 | 30.12 | 2 | 90 | woody_wetland | 36000 | 0.0029080334 |
| 2012 | 33.12 | 2 | 11 | open_water | 99000 | 0.0080116533 |
| 2012 | 33.12 | 2 | 21 | urban_open | 3870000 | 0.3131828114 |
| 2012 | 33.12 | 2 | 22 | urban_low | 3651300 | 0.2954843409 |
| 2012 | 33.12 | 2 | 23 | urban_med | 397800 | 0.0321922797 |
| 2012 | 33.12 | 2 | 24 | urban_high | 180900 | 0.0146394756 |
| 2012 | 33.12 | 2 | 41 | dec_forest | 937800 | 0.0758922068 |
| 2012 | 33.12 | 2 | 42 | eg_forest | 8100 | 0.0006554989 |
| 2012 | 33.12 | 2 | 52 | scrub | 32400 | 0.0026219956 |
| 2012 | 33.12 | 2 | 71 | grass_herb | 62100 | 0.0050254916 |
| 2012 | 33.12 | 2 | 81 | pasture_hay | 815400 | 0.06598689 |
| 2012 | 33.12 | 2 | 82 | crop | 2299500 | 0.1860888565 |
| 2012 | 33.12 | 2 | 90 | woody_wetland | 2700 | 0.0002184996 |
| 2012 | 36.12 | 2 | 11 | open_water | 21600 | 0.0017492711 |
| 2012 | 36.12 | 2 | 21 | urban_open | 1590300 | 0.1287900875 |
| 2012 | 36.12 | 2 | 22 | urban_low | 869400 | 0.0704081633 |
| 2012 | 36.12 | 2 | 23 | urban_med | 156600 | 0.0126822157 |
| 2012 | 36.12 | 2 | 24 | urban_high | 81000 | 0.0065597668 |
| 2012 | 36.12 | 2 | 41 | dec_forest | 1769400 | 0.1432944606 |
| 2012 | 36.12 | 2 | 42 | eg_forest | 34200 | 0.0027696793 |
| 2012 | 36.12 | 2 | 43 | mix_forest | 39600 | 0.0032069971 |
| 2012 | 36.12 | 2 | 71 | grass_herb | 36000 | 0.0029154519 |
| 2012 | 36.12 | 2 | 81 | pasture_hay | 4015800 | 0.3252186589 |
| 2012 | 36.12 | 2 | 82 | crop | 3734100 | 0.3024052478 |
| 2012 | 40.12 | 2 | 11 | open_water | 42300 | 0.0034211676 |
| 2012 | 40.12 | 2 | 21 | urban_open | 656100 | 0.0530644926 |
| 2012 | 40.12 | 2 | 22 | urban_low | 9000 | 0.000727908 |
| 2012 | 40.12 | 2 | 41 | dec_forest | 6845400 | 0.553646819 |
| 2012 | 40.12 | 2 | 42 | eg_forest | 180000 | 0.0145581598 |
| 2012 | 40.12 | 2 | 52 | scrub | 7200 | 0.0005823264 |
| 2012 | 40.12 | 2 | 71 | grass_herb | 74700 | 0.0060416363 |
| 2012 | 40.12 | 2 | 81 | pasture_hay | 1512900 | 0.1223613335 |
| 2012 | 40.12 | 2 | 82 | crop | 3018600 | 0.2441403407 |
| 2012 | 40.12 | 2 | 90 | woody_wetland | 13500 | 0.001091862 |
| 2012 | 40.12 | 2 | 95 | em_herb_wetland | 4500 | 0.000363954 |
| 2012 | 41.12 | 2 | 11 | open_water | 183600 | 0.0148807353 |
| 2012 | 41.12 | 2 | 21 | urban_open | 1543500 | 0.1251002991 |
| 2012 | 41.12 | 2 | 22 | urban_low | 4635900 | 0.3757385659 |
| 2012 | 41.12 | 2 | 23 | urban_med | 4311000 | 0.3494055 |
| 2012 | 41.12 | 2 | 24 | urban_high | 1267200 | 0.1027062514 |
| 2012 | 41.12 | 2 | 41 | dec_forest | 374400 | 0.0303450288 |
| 2012 | 41.12 | 2 | 71 | grass_herb | 22500 | 0.0018236195 |
| 2012 | 42.12 | 2 | 21 | urban_open | 677700 | 0.0548513986 |
| 2012 | 42.12 | 2 | 41 | dec_forest | 10467900 | 0.8472465035 |
| 2012 | 42.12 | 2 | 42 | eg_forest | 429300 | 0.0347465035 |
| 2012 | 42.12 | 2 | 52 | scrub | 48600 | 0.0039335664 |
| 2012 | 42.12 | 2 | 71 | grass_herb | 9900 | 0.0008012821 |
| 2012 | 42.12 | 2 | 81 | pasture_hay | 721800 | 0.0584207459 |
| 2012 | 43.12 | 2 | 11 | open_water | 66600 | 0.0054002773 |
| 2012 | 43.12 | 2 | 21 | urban_open | 659700 | 0.0534919361 |
| 2012 | 43.12 | 2 | 22 | urban_low | 327600 | 0.0265635262 |
| 2012 | 43.12 | 2 | 41 | dec_forest | 1539900 | 0.1248631686 |
| 2012 | 43.12 | 2 | 42 | eg_forest | 12600 | 0.0010216741 |
| 2012 | 43.12 | 2 | 43 | mix_forest | 9900 | 0.0008027439 |
| 2012 | 43.12 | 2 | 71 | grass_herb | 9000 | 0.0007297672 |
| 2012 | 43.12 | 2 | 81 | pasture_hay | 630900 | 0.051156681 |
| 2012 | 43.12 | 2 | 82 | crop | 9042300 | 0.7331971101 |
| 2012 | 43.12 | 2 | 90 | woody_wetland | 34200 | 0.0027731154 |
| 2012 | 44.12 | 2 | 21 | urban_open | 453600 | 0.0367668515 |
| 2012 | 44.12 | 2 | 22 | urban_low | 261000 | 0.0211555296 |
| 2012 | 44.12 | 2 | 41 | dec_forest | 6448500 | 0.5226874818 |
| 2012 | 44.12 | 2 | 42 | eg_forest | 25200 | 0.0020426029 |
| 2012 | 44.12 | 2 | 52 | scrub | 617400 | 0.0500437701 |
| 2012 | 44.12 | 2 | 71 | grass_herb | 371700 | 0.0301283922 |
| 2012 | 44.12 | 2 | 81 | pasture_hay | 524700 | 0.0425299095 |
| 2012 | 44.12 | 2 | 82 | crop | 3129300 | 0.2536475051 |
| 2012 | 44.12 | 2 | 90 | woody_wetland | 505800 | 0.0409979574 |
| 2012 | 45.12 | 2 | 11 | open_water | 26100 | 0.0021160161 |
| 2012 | 45.12 | 2 | 21 | urban_open | 2315700 | 0.1877417001 |
| 2012 | 45.12 | 2 | 22 | urban_low | 3519900 | 0.2853703028 |
| 2012 | 45.12 | 2 | 23 | urban_med | 1361700 | 0.1103976651 |
| 2012 | 45.12 | 2 | 24 | urban_high | 315900 | 0.0256110908 |
| 2012 | 45.12 | 2 | 31 | barren | 5400 | 0.0004377964 |
| 2012 | 45.12 | 2 | 41 | dec_forest | 161100 | 0.0130609267 |
| 2012 | 45.12 | 2 | 71 | grass_herb | 75600 | 0.0061291499 |
| 2012 | 45.12 | 2 | 81 | pasture_hay | 296100 | 0.0240058373 |
| 2012 | 45.12 | 2 | 82 | crop | 4219200 | 0.3420649398 |
| 2012 | 45.12 | 2 | 95 | em_herb_wetland | 37800 | 0.003064575 |
| 2012 | 47.12 | 2 | 11 | open_water | 7200 | 0.0005835582 |
| 2012 | 47.12 | 2 | 21 | urban_open | 2982600 | 0.2417390036 |
| 2012 | 47.12 | 2 | 22 | urban_low | 5120100 | 0.414982858 |
| 2012 | 47.12 | 2 | 23 | urban_med | 2558700 | 0.2073820118 |
| 2012 | 47.12 | 2 | 24 | urban_high | 1349100 | 0.1093442264 |
| 2012 | 47.12 | 2 | 41 | dec_forest | 21600 | 0.0017506747 |
| 2012 | 47.12 | 2 | 82 | crop | 294300 | 0.0238529433 |
| 2012 | 47.12 | 2 | 95 | em_herb_wetland | 4500 | 0.0003647239 |
| 2012 | 50.12 | 2 | 11 | open_water | 2049300 | 0.1662043796 |
| 2012 | 50.12 | 2 | 21 | urban_open | 1170000 | 0.0948905109 |
| 2012 | 50.12 | 2 | 22 | urban_low | 475200 | 0.038540146 |
| 2012 | 50.12 | 2 | 23 | urban_med | 402300 | 0.0326277372 |
| 2012 | 50.12 | 2 | 24 | urban_high | 255600 | 0.020729927 |
| 2012 | 50.12 | 2 | 31 | barren | 641700 | 0.0520437956 |
| 2012 | 50.12 | 2 | 41 | dec_forest | 6451200 | 0.5232116788 |
| 2012 | 50.12 | 2 | 42 | eg_forest | 105300 | 0.008540146 |
| 2012 | 50.12 | 2 | 71 | grass_herb | 89100 | 0.0072262774 |
| 2012 | 50.12 | 2 | 81 | pasture_hay | 671400 | 0.0544525547 |
| 2012 | 50.12 | 2 | 82 | crop | 14400 | 0.0011678832 |
| 2012 | 50.12 | 2 | 95 | em_herb_wetland | 4500 | 0.0003649635 |
| 2012 | 52.12 | 2 | 11 | open_water | 3600 | 0.000291142 |
| 2012 | 52.12 | 2 | 21 | urban_open | 624600 | 0.0505131378 |
| 2012 | 52.12 | 2 | 22 | urban_low | 32400 | 0.002620278 |
| 2012 | 52.12 | 2 | 23 | urban_med | 5400 | 0.000436713 |
| 2012 | 52.12 | 2 | 41 | dec_forest | 6134400 | 0.4961059757 |
| 2012 | 52.12 | 2 | 42 | eg_forest | 62100 | 0.0050221996 |
| 2012 | 52.12 | 2 | 52 | scrub | 6300 | 0.0005094985 |
| 2012 | 52.12 | 2 | 71 | grass_herb | 16200 | 0.001310139 |
| 2012 | 52.12 | 2 | 81 | pasture_hay | 2089800 | 0.1690079336 |
| 2012 | 52.12 | 2 | 82 | crop | 3366900 | 0.2722905597 |
| 2012 | 52.12 | 2 | 90 | woody_wetland | 23400 | 0.001892423 |
| 2012 | 53.12 | 2 | 11 | open_water | 15300 | 0.0012369934 |
| 2012 | 53.12 | 2 | 21 | urban_open | 760500 | 0.0614858473 |
| 2012 | 53.12 | 2 | 22 | urban_low | 218700 | 0.0176817289 |
| 2012 | 53.12 | 2 | 23 | urban_med | 68400 | 0.005530088 |
| 2012 | 53.12 | 2 | 41 | dec_forest | 3732300 | 0.30175362 |
| 2012 | 53.12 | 2 | 42 | eg_forest | 243900 | 0.0197191297 |
| 2012 | 53.12 | 2 | 52 | scrub | 157500 | 0.0127337554 |
| 2012 | 53.12 | 2 | 71 | grass_herb | 874800 | 0.0707269155 |
| 2012 | 53.12 | 2 | 81 | pasture_hay | 4698900 | 0.3799024958 |
| 2012 | 53.12 | 2 | 82 | crop | 1590300 | 0.128574547 |
| 2012 | 53.12 | 2 | 90 | woody_wetland | 8100 | 0.0006548788 |
| 2012 | 54.12 | 2 | 11 | open_water | 15300 | 0.0012402422 |
| 2012 | 54.12 | 2 | 21 | urban_open | 1363500 | 0.1105274677 |
| 2012 | 54.12 | 2 | 22 | urban_low | 800100 | 0.0648573721 |
| 2012 | 54.12 | 2 | 23 | urban_med | 118800 | 0.009630116 |
| 2012 | 54.12 | 2 | 24 | urban_high | 10800 | 0.0008754651 |
| 2012 | 54.12 | 2 | 41 | dec_forest | 1478700 | 0.119865762 |
| 2012 | 54.12 | 2 | 42 | eg_forest | 5400 | 0.0004377325 |
| 2012 | 54.12 | 2 | 71 | grass_herb | 80100 | 0.0064930328 |
| 2012 | 54.12 | 2 | 81 | pasture_hay | 1368900 | 0.1109652003 |
| 2012 | 54.12 | 2 | 82 | crop | 7090200 | 0.5747428321 |
| 2012 | 54.12 | 2 | 95 | em_herb_wetland | 4500 | 0.0003647771 |
| 2012 | 101.12 | 2 | 11 | open_water | 18900 | 0.0015297203 |
| 2012 | 101.12 | 2 | 21 | urban_open | 3427200 | 0.2773892774 |
| 2012 | 101.12 | 2 | 22 | urban_low | 1448100 | 0.117205711 |
| 2012 | 101.12 | 2 | 23 | urban_med | 477900 | 0.0386800699 |
| 2012 | 101.12 | 2 | 24 | urban_high | 72900 | 0.0059003497 |
| 2012 | 101.12 | 2 | 31 | barren | 1800 | 0.0001456876 |
| 2012 | 101.12 | 2 | 41 | dec_forest | 1758600 | 0.1423368298 |
| 2012 | 101.12 | 2 | 42 | eg_forest | 128700 | 0.0104166667 |
| 2012 | 101.12 | 2 | 71 | grass_herb | 126900 | 0.010270979 |
| 2012 | 101.12 | 2 | 81 | pasture_hay | 1227600 | 0.0993589744 |
| 2012 | 101.12 | 2 | 82 | crop | 3666600 | 0.2967657343 |
| 2012 | 102.12 | 2 | 11 | open_water | 612000 | 0.049580751 |
| 2012 | 102.12 | 2 | 21 | urban_open | 4209300 | 0.3410134889 |
| 2012 | 102.12 | 2 | 22 | urban_low | 5166000 | 0.4185198688 |
| 2012 | 102.12 | 2 | 23 | urban_med | 506700 | 0.0410499453 |
| 2012 | 102.12 | 2 | 24 | urban_high | 135000 | 0.0109369304 |
| 2012 | 102.12 | 2 | 41 | dec_forest | 1263600 | 0.1023696682 |
| 2012 | 102.12 | 2 | 42 | eg_forest | 24300 | 0.0019686475 |
| 2012 | 102.12 | 2 | 71 | grass_herb | 189900 | 0.0153846154 |
| 2012 | 102.12 | 2 | 81 | pasture_hay | 60300 | 0.0048851622 |
| 2012 | 102.12 | 2 | 90 | woody_wetland | 176400 | 0.0142909223 |
| 2012 | 104.12 | 2 | 21 | urban_open | 365400 | 0.0295724379 |
| 2012 | 104.12 | 2 | 22 | urban_low | 21600 | 0.0017481244 |
| 2012 | 104.12 | 2 | 41 | dec_forest | 23400 | 0.0018938014 |
| 2012 | 104.12 | 2 | 71 | grass_herb | 38700 | 0.0031320562 |
| 2012 | 104.12 | 2 | 81 | pasture_hay | 122400 | 0.0099060383 |
| 2012 | 104.12 | 2 | 82 | crop | 11784600 | 0.9537475417 |
| 2012 | 107.12 | 2 | 21 | urban_open | 785700 | 0.0636111921 |
| 2012 | 107.12 | 2 | 22 | urban_low | 60300 | 0.0048819586 |
| 2012 | 107.12 | 2 | 23 | urban_med | 4500 | 0.0003643253 |
| 2012 | 107.12 | 2 | 41 | dec_forest | 1325700 | 0.1073302244 |
| 2012 | 107.12 | 2 | 43 | mix_forest | 287100 | 0.0232439522 |
| 2012 | 107.12 | 2 | 81 | pasture_hay | 450900 | 0.036505392 |
| 2012 | 107.12 | 2 | 82 | crop | 9437400 | 0.7640629554 |
| 2012 | 108.12 | 2 | 21 | urban_open | 2342700 | 0.1896953797 |
| 2012 | 108.12 | 2 | 22 | urban_low | 5457600 | 0.4419180877 |
| 2012 | 108.12 | 2 | 23 | urban_med | 2965500 | 0.2401253462 |
| 2012 | 108.12 | 2 | 24 | urban_high | 1089900 | 0.0882524413 |
| 2012 | 108.12 | 2 | 41 | dec_forest | 434700 | 0.0351989506 |
| 2012 | 108.12 | 2 | 71 | grass_herb | 59400 | 0.0048097945 |
| 2012 | 111.12 | 2 | 11 | open_water | 117900 | 0.009548105 |
| 2012 | 111.12 | 2 | 21 | urban_open | 3914100 | 0.3169825073 |
| 2012 | 111.12 | 2 | 22 | urban_low | 5541300 | 0.4487609329 |
| 2012 | 111.12 | 2 | 23 | urban_med | 1328400 | 0.1075801749 |
| 2012 | 111.12 | 2 | 24 | urban_high | 461700 | 0.0373906706 |
| 2012 | 111.12 | 2 | 41 | dec_forest | 605700 | 0.0490524781 |
| 2012 | 111.12 | 2 | 42 | eg_forest | 18000 | 0.0014577259 |
| 2012 | 111.12 | 2 | 71 | grass_herb | 107100 | 0.0086734694 |
| 2012 | 111.12 | 2 | 81 | pasture_hay | 132300 | 0.0107142857 |
| 2012 | 111.12 | 2 | 82 | crop | 75600 | 0.006122449 |
| 2012 | 111.12 | 2 | 90 | woody_wetland | 45900 | 0.0037172012 |
| 2012 | 112.12 | 2 | 11 | open_water | 715500 | 0.0579277179 |
| 2012 | 112.12 | 2 | 21 | urban_open | 4397400 | 0.3560186535 |
| 2012 | 112.12 | 2 | 22 | urban_low | 4620600 | 0.3740891868 |
| 2012 | 112.12 | 2 | 23 | urban_med | 493200 | 0.0399300495 |
| 2012 | 112.12 | 2 | 24 | urban_high | 153900 | 0.0124599242 |
| 2012 | 112.12 | 2 | 41 | dec_forest | 956700 | 0.0774555523 |
| 2012 | 112.12 | 2 | 42 | eg_forest | 64800 | 0.0052462839 |
| 2012 | 112.12 | 2 | 43 | mix_forest | 29700 | 0.0024045468 |
| 2012 | 112.12 | 2 | 52 | scrub | 12600 | 0.0010201108 |
| 2012 | 112.12 | 2 | 71 | grass_herb | 142200 | 0.0115126785 |
| 2012 | 112.12 | 2 | 81 | pasture_hay | 266400 | 0.021568056 |
| 2012 | 112.12 | 2 | 82 | crop | 457200 | 0.0370154474 |
| 2012 | 112.12 | 2 | 90 | woody_wetland | 31500 | 0.0025502769 |
| 2012 | 112.12 | 2 | 95 | em_herb_wetland | 9900 | 0.0008015156 |
| 2012 | 113.12 | 2 | 21 | urban_open | 1012500 | 0.0820389412 |
| 2012 | 113.12 | 2 | 22 | urban_low | 7555500 | 0.6121928097 |
| 2012 | 113.12 | 2 | 23 | urban_med | 2965500 | 0.2402829432 |
| 2012 | 113.12 | 2 | 24 | urban_high | 747000 | 0.0605265077 |
| 2012 | 113.12 | 2 | 41 | dec_forest | 55800 | 0.0045212572 |
| 2012 | 113.12 | 2 | 71 | grass_herb | 5400 | 0.000437541 |
| 2012 | 115.12 | 2 | 11 | open_water | 71100 | 0.0057580175 |
| 2012 | 115.12 | 2 | 21 | urban_open | 3042900 | 0.2464285714 |
| 2012 | 115.12 | 2 | 22 | urban_low | 4008600 | 0.3246355685 |
| 2012 | 115.12 | 2 | 23 | urban_med | 1199700 | 0.0971574344 |
| 2012 | 115.12 | 2 | 24 | urban_high | 358200 | 0.0290087464 |
| 2012 | 115.12 | 2 | 41 | dec_forest | 3164400 | 0.2562682216 |
| 2012 | 115.12 | 2 | 42 | eg_forest | 45900 | 0.0037172012 |
| 2012 | 115.12 | 2 | 71 | grass_herb | 123300 | 0.0099854227 |
| 2012 | 115.12 | 2 | 81 | pasture_hay | 327600 | 0.0265306122 |
| 2012 | 115.12 | 2 | 90 | woody_wetland | 6300 | 0.0005102041 |
| 2012 | 9.12 | 3 | 11 | open_water | 34200 | 0.0012299725 |
| 2012 | 9.12 | 3 | 21 | urban_open | 8887500 | 0.3196310083 |
| 2012 | 9.12 | 3 | 22 | urban_low | 7163100 | 0.2576145007 |
| 2012 | 9.12 | 3 | 23 | urban_med | 2304000 | 0.0828613044 |
| 2012 | 9.12 | 3 | 24 | urban_high | 1139400 | 0.0409775045 |
| 2012 | 9.12 | 3 | 31 | barren | 99000 | 0.0035604467 |
| 2012 | 9.12 | 3 | 41 | dec_forest | 4636800 | 0.1667583751 |
| 2012 | 9.12 | 3 | 42 | eg_forest | 72000 | 0.0025894158 |
| 2012 | 9.12 | 3 | 43 | mix_forest | 6300 | 0.0002265739 |
| 2012 | 9.12 | 3 | 52 | scrub | 7200 | 0.0002589416 |
| 2012 | 9.12 | 3 | 71 | grass_herb | 528300 | 0.0189998382 |
| 2012 | 9.12 | 3 | 81 | pasture_hay | 1845900 | 0.0663861466 |
| 2012 | 9.12 | 3 | 82 | crop | 432900 | 0.0155688623 |
| 2012 | 9.12 | 3 | 90 | woody_wetland | 648900 | 0.0233371096 |
| 2012 | 11.12 | 3 | 11 | open_water | 405900 | 0.0146063413 |
| 2012 | 11.12 | 3 | 21 | urban_open | 1425600 | 0.0513003206 |
| 2012 | 11.12 | 3 | 22 | urban_low | 1162800 | 0.0418434433 |
| 2012 | 11.12 | 3 | 23 | urban_med | 486900 | 0.0175211322 |
| 2012 | 11.12 | 3 | 24 | urban_high | 83700 | 0.0030119506 |
| 2012 | 11.12 | 3 | 41 | dec_forest | 5204700 | 0.1872915115 |
| 2012 | 11.12 | 3 | 42 | eg_forest | 50400 | 0.0018136477 |
| 2012 | 11.12 | 3 | 43 | mix_forest | 4500 | 0.0001619328 |
| 2012 | 11.12 | 3 | 52 | scrub | 129600 | 0.0046636655 |
| 2012 | 11.12 | 3 | 71 | grass_herb | 26100 | 0.0009392104 |
| 2012 | 11.12 | 3 | 81 | pasture_hay | 3453300 | 0.1242672539 |
| 2012 | 11.12 | 3 | 82 | crop | 15332400 | 0.5517375393 |
| 2012 | 11.12 | 3 | 90 | woody_wetland | 22500 | 0.0008096642 |
| 2012 | 11.12 | 3 | 95 | em_herb_wetland | 900 | 3.24E-05 |
| 2012 | 12.12 | 3 | 11 | open_water | 10800 | 0.0003884375 |
| 2012 | 12.12 | 3 | 21 | urban_open | 8750700 | 0.3147314926 |
| 2012 | 12.12 | 3 | 22 | urban_low | 9235800 | 0.3321788107 |
| 2012 | 12.12 | 3 | 23 | urban_med | 4331700 | 0.1557958113 |
| 2012 | 12.12 | 3 | 24 | urban_high | 3442500 | 0.1238144563 |
| 2012 | 12.12 | 3 | 41 | dec_forest | 1653300 | 0.0594633088 |
| 2012 | 12.12 | 3 | 42 | eg_forest | 53100 | 0.0019098178 |
| 2012 | 12.12 | 3 | 71 | grass_herb | 147600 | 0.005308646 |
| 2012 | 12.12 | 3 | 81 | pasture_hay | 93600 | 0.0033664584 |
| 2012 | 12.12 | 3 | 90 | woody_wetland | 84600 | 0.0030427605 |
| 2012 | 17.12 | 3 | 11 | open_water | 400500 | 0.0143980328 |
| 2012 | 17.12 | 3 | 21 | urban_open | 9654300 | 0.3470734785 |
| 2012 | 17.12 | 3 | 22 | urban_low | 6458400 | 0.2321804122 |
| 2012 | 17.12 | 3 | 23 | urban_med | 558000 | 0.0200601805 |
| 2012 | 17.12 | 3 | 24 | urban_high | 234000 | 0.0084123338 |
| 2012 | 17.12 | 3 | 31 | barren | 65700 | 0.0023619245 |
| 2012 | 17.12 | 3 | 41 | dec_forest | 4191300 | 0.15067784 |
| 2012 | 17.12 | 3 | 42 | eg_forest | 97200 | 0.003494354 |
| 2012 | 17.12 | 3 | 43 | mix_forest | 17100 | 0.0006147475 |
| 2012 | 17.12 | 3 | 52 | scrub | 12600 | 0.0004529718 |
| 2012 | 17.12 | 3 | 71 | grass_herb | 352800 | 0.0126832109 |
| 2012 | 17.12 | 3 | 81 | pasture_hay | 2451600 | 0.0881353739 |
| 2012 | 17.12 | 3 | 82 | crop | 3176100 | 0.1141812534 |
| 2012 | 17.12 | 3 | 90 | woody_wetland | 122400 | 0.0044002977 |
| 2012 | 17.12 | 3 | 95 | em_herb_wetland | 24300 | 0.0008735885 |
| 2012 | 20.12 | 3 | 11 | open_water | 461700 | 0.0166094671 |
| 2012 | 20.12 | 3 | 21 | urban_open | 1177200 | 0.0423492845 |
| 2012 | 20.12 | 3 | 22 | urban_low | 407700 | 0.0146668393 |
| 2012 | 20.12 | 3 | 23 | urban_med | 47700 | 0.0017159878 |
| 2012 | 20.12 | 3 | 24 | urban_high | 19800 | 0.0007122968 |
| 2012 | 20.12 | 3 | 41 | dec_forest | 2412900 | 0.0868030823 |
| 2012 | 20.12 | 3 | 42 | eg_forest | 18000 | 0.0006475426 |
| 2012 | 20.12 | 3 | 43 | mix_forest | 45000 | 0.0016188564 |
| 2012 | 20.12 | 3 | 52 | scrub | 7200 | 0.000259017 |
| 2012 | 20.12 | 3 | 81 | pasture_hay | 2626200 | 0.0944764618 |
| 2012 | 20.12 | 3 | 82 | crop | 20469600 | 0.7363854173 |
| 2012 | 20.12 | 3 | 90 | woody_wetland | 96300 | 0.0034643528 |
| 2012 | 20.12 | 3 | 95 | em_herb_wetland | 8100 | 0.0002913942 |
| 2012 | 21.12 | 3 | 11 | open_water | 290700 | 0.0104595058 |
| 2012 | 21.12 | 3 | 21 | urban_open | 8924400 | 0.3211035912 |
| 2012 | 21.12 | 3 | 22 | urban_low | 4231800 | 0.1522619086 |
| 2012 | 21.12 | 3 | 23 | urban_med | 1890900 | 0.0680353615 |
| 2012 | 21.12 | 3 | 24 | urban_high | 398700 | 0.0143453904 |
| 2012 | 21.12 | 3 | 41 | dec_forest | 9977400 | 0.3589909653 |
| 2012 | 21.12 | 3 | 42 | eg_forest | 446400 | 0.016061656 |
| 2012 | 21.12 | 3 | 43 | mix_forest | 16200 | 0.0005828827 |
| 2012 | 21.12 | 3 | 52 | scrub | 78300 | 0.0028172663 |
| 2012 | 21.12 | 3 | 71 | grass_herb | 229500 | 0.0082575046 |
| 2012 | 21.12 | 3 | 81 | pasture_hay | 961200 | 0.0345843723 |
| 2012 | 21.12 | 3 | 82 | crop | 309600 | 0.0111395356 |
| 2012 | 21.12 | 3 | 90 | woody_wetland | 20700 | 0.0007447945 |
| 2012 | 21.12 | 3 | 95 | em_herb_wetland | 17100 | 0.000615265 |
| 2012 | 26.12 | 3 | 11 | open_water | 9900 | 0.0003564254 |
| 2012 | 26.12 | 3 | 21 | urban_open | 2675700 | 0.0963320588 |
| 2012 | 26.12 | 3 | 22 | urban_low | 756000 | 0.0272179379 |
| 2012 | 26.12 | 3 | 23 | urban_med | 119700 | 0.0043095068 |
| 2012 | 26.12 | 3 | 24 | urban_high | 18900 | 0.0006804484 |
| 2012 | 26.12 | 3 | 31 | barren | 135000 | 0.0048603461 |
| 2012 | 26.12 | 3 | 41 | dec_forest | 13098600 | 0.4715831767 |
| 2012 | 26.12 | 3 | 42 | eg_forest | 214200 | 0.0077117491 |
| 2012 | 26.12 | 3 | 52 | scrub | 19800 | 0.0007128508 |
| 2012 | 26.12 | 3 | 71 | grass_herb | 171900 | 0.0061888406 |
| 2012 | 26.12 | 3 | 81 | pasture_hay | 9486900 | 0.3415527186 |
| 2012 | 26.12 | 3 | 82 | crop | 1064700 | 0.0383319292 |
| 2012 | 26.12 | 3 | 90 | woody_wetland | 4500 | 0.0001620115 |
| 2012 | 27.12 | 3 | 11 | open_water | 91800 | 0.0033051424 |
| 2012 | 27.12 | 3 | 21 | urban_open | 1719900 | 0.0619228152 |
| 2012 | 27.12 | 3 | 22 | urban_low | 624600 | 0.0224879297 |
| 2012 | 27.12 | 3 | 23 | urban_med | 56700 | 0.0020414115 |
| 2012 | 27.12 | 3 | 24 | urban_high | 14400 | 0.0005184537 |
| 2012 | 27.12 | 3 | 31 | barren | 42300 | 0.0015229578 |
| 2012 | 27.12 | 3 | 41 | dec_forest | 3751200 | 0.1350571919 |
| 2012 | 27.12 | 3 | 42 | eg_forest | 80100 | 0.0028838988 |
| 2012 | 27.12 | 3 | 43 | mix_forest | 4500 | 0.0001620168 |
| 2012 | 27.12 | 3 | 71 | grass_herb | 738000 | 0.0265707527 |
| 2012 | 27.12 | 3 | 81 | pasture_hay | 1594800 | 0.0574187486 |
| 2012 | 27.12 | 3 | 82 | crop | 19007100 | 0.6843264962 |
| 2012 | 27.12 | 3 | 90 | woody_wetland | 49500 | 0.0017821846 |
| 2012 | 28.12 | 3 | 11 | open_water | 94500 | 0.0033992683 |
| 2012 | 28.12 | 3 | 21 | urban_open | 4512600 | 0.1623231571 |
| 2012 | 28.12 | 3 | 22 | urban_low | 2373300 | 0.0853701965 |
| 2012 | 28.12 | 3 | 23 | urban_med | 343800 | 0.012366862 |
| 2012 | 28.12 | 3 | 24 | urban_high | 121500 | 0.0043704879 |
| 2012 | 28.12 | 3 | 31 | barren | 82800 | 0.0029784066 |
| 2012 | 28.12 | 3 | 41 | dec_forest | 11096100 | 0.399138852 |
| 2012 | 28.12 | 3 | 42 | eg_forest | 269100 | 0.0096798213 |
| 2012 | 28.12 | 3 | 71 | grass_herb | 1062900 | 0.0382336754 |
| 2012 | 28.12 | 3 | 81 | pasture_hay | 2942100 | 0.1058305546 |
| 2012 | 28.12 | 3 | 82 | crop | 4552200 | 0.1637476124 |
| 2012 | 28.12 | 3 | 90 | woody_wetland | 330300 | 0.0118812522 |
| 2012 | 28.12 | 3 | 95 | em_herb_wetland | 18900 | 0.0006798537 |
| 2012 | 30.12 | 3 | 11 | open_water | 74700 | 0.0026898273 |
| 2012 | 30.12 | 3 | 21 | urban_open | 6875100 | 0.2475613313 |
| 2012 | 30.12 | 3 | 22 | urban_low | 7907400 | 0.2847327997 |
| 2012 | 30.12 | 3 | 23 | urban_med | 2485800 | 0.0895096737 |
| 2012 | 30.12 | 3 | 24 | urban_high | 552600 | 0.0198982403 |
| 2012 | 30.12 | 3 | 41 | dec_forest | 4815900 | 0.1734128399 |
| 2012 | 30.12 | 3 | 42 | eg_forest | 128700 | 0.0046342807 |
| 2012 | 30.12 | 3 | 52 | scrub | 125100 | 0.0045046505 |
| 2012 | 30.12 | 3 | 71 | grass_herb | 241200 | 0.0086852254 |
| 2012 | 30.12 | 3 | 81 | pasture_hay | 1836000 | 0.0661114172 |
| 2012 | 30.12 | 3 | 82 | crop | 2589300 | 0.0932365428 |
| 2012 | 30.12 | 3 | 90 | woody_wetland | 135000 | 0.0048611336 |
| 2012 | 30.12 | 3 | 95 | em_herb_wetland | 4500 | 0.0001620378 |
| 2012 | 33.12 | 3 | 11 | open_water | 132300 | 0.0047585135 |
| 2012 | 33.12 | 3 | 21 | urban_open | 8389800 | 0.3017609737 |
| 2012 | 33.12 | 3 | 22 | urban_low | 7417800 | 0.2668004661 |
| 2012 | 33.12 | 3 | 23 | urban_med | 1107000 | 0.0398161336 |
| 2012 | 33.12 | 3 | 24 | urban_high | 438300 | 0.0157645992 |
| 2012 | 33.12 | 3 | 41 | dec_forest | 2475900 | 0.0890521818 |
| 2012 | 33.12 | 3 | 42 | eg_forest | 8100 | 0.0002913376 |
| 2012 | 33.12 | 3 | 52 | scrub | 96300 | 0.0034636799 |
| 2012 | 33.12 | 3 | 71 | grass_herb | 236700 | 0.008513531 |
| 2012 | 33.12 | 3 | 81 | pasture_hay | 1630800 | 0.0586559627 |
| 2012 | 33.12 | 3 | 82 | crop | 5818500 | 0.2092774828 |
| 2012 | 33.12 | 3 | 90 | woody_wetland | 47700 | 0.0017156545 |
| 2012 | 33.12 | 3 | 95 | em_herb_wetland | 3600 | 0.0001294834 |
| 2012 | 36.12 | 3 | 11 | open_water | 324000 | 0.0116568986 |
| 2012 | 36.12 | 3 | 21 | urban_open | 3705300 | 0.1333095878 |
| 2012 | 36.12 | 3 | 22 | urban_low | 2728800 | 0.0981769906 |
| 2012 | 36.12 | 3 | 23 | urban_med | 386100 | 0.0138911375 |
| 2012 | 36.12 | 3 | 24 | urban_high | 117000 | 0.0042094356 |
| 2012 | 36.12 | 3 | 31 | barren | 8100 | 0.0002914225 |
| 2012 | 36.12 | 3 | 41 | dec_forest | 3577500 | 0.1287115889 |
| 2012 | 36.12 | 3 | 42 | eg_forest | 94500 | 0.0033999288 |
| 2012 | 36.12 | 3 | 43 | mix_forest | 57600 | 0.0020723375 |
| 2012 | 36.12 | 3 | 71 | grass_herb | 76500 | 0.0027523233 |
| 2012 | 36.12 | 3 | 81 | pasture_hay | 8123400 | 0.2922643526 |
| 2012 | 36.12 | 3 | 82 | crop | 8568900 | 0.3082925882 |
| 2012 | 36.12 | 3 | 95 | em_herb_wetland | 27000 | 0.0009714082 |
| 2012 | 40.12 | 3 | 11 | open_water | 83700 | 0.0030111705 |
| 2012 | 40.12 | 3 | 21 | urban_open | 1272600 | 0.0457827424 |
| 2012 | 40.12 | 3 | 22 | urban_low | 13500 | 0.0004856727 |
| 2012 | 40.12 | 3 | 41 | dec_forest | 16816500 | 0.6049862393 |
| 2012 | 40.12 | 3 | 42 | eg_forest | 646200 | 0.0232475312 |
| 2012 | 40.12 | 3 | 52 | scrub | 7200 | 0.0002590254 |
| 2012 | 40.12 | 3 | 71 | grass_herb | 233100 | 0.0083859479 |
| 2012 | 40.12 | 3 | 81 | pasture_hay | 3931200 | 0.1414278776 |
| 2012 | 40.12 | 3 | 82 | crop | 4770900 | 0.1716367169 |
| 2012 | 40.12 | 3 | 90 | woody_wetland | 17100 | 0.0006151854 |
| 2012 | 40.12 | 3 | 95 | em_herb_wetland | 4500 | 0.0001618909 |
| 2012 | 41.12 | 3 | 11 | open_water | 231300 | 0.0083225389 |
| 2012 | 41.12 | 3 | 21 | urban_open | 4327200 | 0.1556994819 |
| 2012 | 41.12 | 3 | 22 | urban_low | 10904400 | 0.392357513 |
| 2012 | 41.12 | 3 | 23 | urban_med | 8877600 | 0.3194300518 |
| 2012 | 41.12 | 3 | 24 | urban_high | 2664900 | 0.0958873057 |
| 2012 | 41.12 | 3 | 41 | dec_forest | 736200 | 0.0264896373 |
| 2012 | 41.12 | 3 | 71 | grass_herb | 50400 | 0.0018134715 |
| 2012 | 42.12 | 3 | 21 | urban_open | 1407600 | 0.0506493086 |
| 2012 | 42.12 | 3 | 41 | dec_forest | 23101200 | 0.8312445351 |
| 2012 | 42.12 | 3 | 42 | eg_forest | 1401300 | 0.0504226173 |
| 2012 | 42.12 | 3 | 52 | scrub | 50400 | 0.0018135302 |
| 2012 | 42.12 | 3 | 71 | grass_herb | 61200 | 0.0022021439 |
| 2012 | 42.12 | 3 | 81 | pasture_hay | 1420200 | 0.0511026911 |
| 2012 | 42.12 | 3 | 82 | crop | 349200 | 0.0125651737 |
| 2012 | 43.12 | 3 | 11 | open_water | 492300 | 0.0176994014 |
| 2012 | 43.12 | 3 | 21 | urban_open | 1242900 | 0.044685326 |
| 2012 | 43.12 | 3 | 22 | urban_low | 552600 | 0.0198673354 |
| 2012 | 43.12 | 3 | 23 | urban_med | 56700 | 0.0020385051 |
| 2012 | 43.12 | 3 | 24 | urban_high | 13500 | 0.0004853584 |
| 2012 | 43.12 | 3 | 31 | barren | 6300 | 0.0002265006 |
| 2012 | 43.12 | 3 | 41 | dec_forest | 3534300 | 0.1270668177 |
| 2012 | 43.12 | 3 | 42 | eg_forest | 24300 | 0.000873645 |
| 2012 | 43.12 | 3 | 43 | mix_forest | 9900 | 0.0003559295 |
| 2012 | 43.12 | 3 | 52 | scrub | 7200 | 0.0002588578 |
| 2012 | 43.12 | 3 | 71 | grass_herb | 23400 | 0.0008412878 |
| 2012 | 43.12 | 3 | 81 | pasture_hay | 1823400 | 0.0655557353 |
| 2012 | 43.12 | 3 | 82 | crop | 19988100 | 0.7186215823 |
| 2012 | 43.12 | 3 | 90 | woody_wetland | 39600 | 0.0014237178 |
| 2012 | 44.12 | 3 | 11 | open_water | 135000 | 0.0048546832 |
| 2012 | 44.12 | 3 | 21 | urban_open | 1107000 | 0.0398084018 |
| 2012 | 44.12 | 3 | 22 | urban_low | 530100 | 0.0190627225 |
| 2012 | 44.12 | 3 | 23 | urban_med | 4500 | 0.0001618228 |
| 2012 | 44.12 | 3 | 41 | dec_forest | 15309900 | 0.5505534339 |
| 2012 | 44.12 | 3 | 42 | eg_forest | 63900 | 0.0022978834 |
| 2012 | 44.12 | 3 | 52 | scrub | 1080900 | 0.0388698298 |
| 2012 | 44.12 | 3 | 71 | grass_herb | 1036800 | 0.0372839666 |
| 2012 | 44.12 | 3 | 81 | pasture_hay | 1551600 | 0.0557964917 |
| 2012 | 44.12 | 3 | 82 | crop | 5900400 | 0.2121820183 |
| 2012 | 44.12 | 3 | 90 | woody_wetland | 1082700 | 0.0389345589 |
| 2012 | 44.12 | 3 | 95 | em_herb_wetland | 5400 | 0.0001941873 |
| 2012 | 45.12 | 3 | 11 | open_water | 26100 | 0.0009386632 |
| 2012 | 45.12 | 3 | 21 | urban_open | 3115800 | 0.1120569671 |
| 2012 | 45.12 | 3 | 22 | urban_low | 4410000 | 0.1586017155 |
| 2012 | 45.12 | 3 | 23 | urban_med | 1752300 | 0.0630199061 |
| 2012 | 45.12 | 3 | 24 | urban_high | 564300 | 0.020294546 |
| 2012 | 45.12 | 3 | 31 | barren | 5400 | 0.0001942062 |
| 2012 | 45.12 | 3 | 41 | dec_forest | 398700 | 0.0143388898 |
| 2012 | 45.12 | 3 | 71 | grass_herb | 163800 | 0.0058909209 |
| 2012 | 45.12 | 3 | 81 | pasture_hay | 1361700 | 0.0489723256 |
| 2012 | 45.12 | 3 | 82 | crop | 15964200 | 0.5741382101 |
| 2012 | 45.12 | 3 | 95 | em_herb_wetland | 43200 | 0.0015536495 |
| 2012 | 47.12 | 3 | 11 | open_water | 32400 | 0.0011644079 |
| 2012 | 47.12 | 3 | 21 | urban_open | 5577300 | 0.2004398874 |
| 2012 | 47.12 | 3 | 22 | urban_low | 9765900 | 0.3509719572 |
| 2012 | 47.12 | 3 | 23 | urban_med | 6001200 | 0.2156742245 |
| 2012 | 47.12 | 3 | 24 | urban_high | 3702600 | 0.1330659508 |
| 2012 | 47.12 | 3 | 41 | dec_forest | 404100 | 0.0145227545 |
| 2012 | 47.12 | 3 | 43 | mix_forest | 7200 | 0.0002587573 |
| 2012 | 47.12 | 3 | 71 | grass_herb | 33300 | 0.0011967526 |
| 2012 | 47.12 | 3 | 81 | pasture_hay | 180900 | 0.0065012776 |
| 2012 | 47.12 | 3 | 82 | crop | 2115900 | 0.0760423068 |
| 2012 | 47.12 | 3 | 95 | em_herb_wetland | 4500 | 0.0001617233 |
| 2012 | 50.12 | 3 | 11 | open_water | 3540600 | 0.1272891995 |
| 2012 | 50.12 | 3 | 21 | urban_open | 2428200 | 0.087296965 |
| 2012 | 50.12 | 3 | 22 | urban_low | 711000 | 0.0255613797 |
| 2012 | 50.12 | 3 | 23 | urban_med | 657900 | 0.0236523652 |
| 2012 | 50.12 | 3 | 24 | urban_high | 471600 | 0.0169546366 |
| 2012 | 50.12 | 3 | 31 | barren | 899100 | 0.0323238206 |
| 2012 | 50.12 | 3 | 41 | dec_forest | 16659900 | 0.5989451886 |
| 2012 | 50.12 | 3 | 42 | eg_forest | 177300 | 0.0063741668 |
| 2012 | 50.12 | 3 | 43 | mix_forest | 5400 | 0.0001941371 |
| 2012 | 50.12 | 3 | 71 | grass_herb | 289800 | 0.0104186889 |
| 2012 | 50.12 | 3 | 81 | pasture_hay | 1855800 | 0.0667184365 |
| 2012 | 50.12 | 3 | 82 | crop | 95400 | 0.0034297547 |
| 2012 | 50.12 | 3 | 90 | woody_wetland | 14400 | 0.0005176988 |
| 2012 | 50.12 | 3 | 95 | em_herb_wetland | 9000 | 0.0003235618 |
| 2012 | 52.12 | 3 | 11 | open_water | 14400 | 0.0005182858 |
| 2012 | 52.12 | 3 | 21 | urban_open | 1893600 | 0.0681545787 |
| 2012 | 52.12 | 3 | 22 | urban_low | 69300 | 0.0024942503 |
| 2012 | 52.12 | 3 | 23 | urban_med | 5400 | 0.0001943572 |
| 2012 | 52.12 | 3 | 24 | urban_high | 4500 | 0.0001619643 |
| 2012 | 52.12 | 3 | 31 | barren | 12600 | 0.0004535 |
| 2012 | 52.12 | 3 | 41 | dec_forest | 14553000 | 0.5237925561 |
| 2012 | 52.12 | 3 | 42 | eg_forest | 219600 | 0.007903858 |
| 2012 | 52.12 | 3 | 52 | scrub | 10800 | 0.0003887143 |
| 2012 | 52.12 | 3 | 71 | grass_herb | 88200 | 0.0031745003 |
| 2012 | 52.12 | 3 | 81 | pasture_hay | 4800600 | 0.1727835185 |
| 2012 | 52.12 | 3 | 82 | crop | 6032700 | 0.2171293447 |
| 2012 | 52.12 | 3 | 90 | woody_wetland | 79200 | 0.0028505717 |
| 2012 | 53.12 | 3 | 11 | open_water | 31500 | 0.0011329794 |
| 2012 | 53.12 | 3 | 21 | urban_open | 2622600 | 0.0943286288 |
| 2012 | 53.12 | 3 | 22 | urban_low | 1398600 | 0.0503042859 |
| 2012 | 53.12 | 3 | 23 | urban_med | 242100 | 0.0087077561 |
| 2012 | 53.12 | 3 | 24 | urban_high | 8100 | 0.0002913376 |
| 2012 | 53.12 | 3 | 41 | dec_forest | 7174800 | 0.2580603392 |
| 2012 | 53.12 | 3 | 42 | eg_forest | 303300 | 0.0109089732 |
| 2012 | 53.12 | 3 | 43 | mix_forest | 4500 | 0.0001618542 |
| 2012 | 53.12 | 3 | 52 | scrub | 288000 | 0.0103586689 |
| 2012 | 53.12 | 3 | 71 | grass_herb | 1271700 | 0.0457399974 |
| 2012 | 53.12 | 3 | 81 | pasture_hay | 11064600 | 0.3979671112 |
| 2012 | 53.12 | 3 | 82 | crop | 3384900 | 0.1217467305 |
| 2012 | 53.12 | 3 | 90 | woody_wetland | 8100 | 0.0002913376 |
| 2012 | 54.12 | 3 | 11 | open_water | 40500 | 0.0014578204 |
| 2012 | 54.12 | 3 | 21 | urban_open | 3373200 | 0.121420241 |
| 2012 | 54.12 | 3 | 22 | urban_low | 2102400 | 0.0756770766 |
| 2012 | 54.12 | 3 | 23 | urban_med | 271800 | 0.0097835947 |
| 2012 | 54.12 | 3 | 24 | urban_high | 11700 | 0.0004211481 |
| 2012 | 54.12 | 3 | 41 | dec_forest | 4310100 | 0.1551444862 |
| 2012 | 54.12 | 3 | 42 | eg_forest | 19800 | 0.0007127122 |
| 2012 | 54.12 | 3 | 43 | mix_forest | 47700 | 0.0017169885 |
| 2012 | 54.12 | 3 | 71 | grass_herb | 297900 | 0.0107230789 |
| 2012 | 54.12 | 3 | 81 | pasture_hay | 2391300 | 0.0860761954 |
| 2012 | 54.12 | 3 | 82 | crop | 14895900 | 0.5361863418 |
| 2012 | 54.12 | 3 | 95 | em_herb_wetland | 18900 | 0.0006803162 |
| 2012 | 101.12 | 3 | 11 | open_water | 101700 | 0.0036611048 |
| 2012 | 101.12 | 3 | 21 | urban_open | 5319900 | 0.1915114207 |
| 2012 | 101.12 | 3 | 22 | urban_low | 3438900 | 0.1237971813 |
| 2012 | 101.12 | 3 | 23 | urban_med | 1620000 | 0.0583184837 |
| 2012 | 101.12 | 3 | 24 | urban_high | 697500 | 0.0251093472 |
| 2012 | 101.12 | 3 | 31 | barren | 51300 | 0.001846752 |
| 2012 | 101.12 | 3 | 41 | dec_forest | 3530700 | 0.1271018954 |
| 2012 | 101.12 | 3 | 42 | eg_forest | 179100 | 0.0064474324 |
| 2012 | 101.12 | 3 | 71 | grass_herb | 242100 | 0.0087153734 |
| 2012 | 101.12 | 3 | 81 | pasture_hay | 2692800 | 0.0969382796 |
| 2012 | 101.12 | 3 | 82 | crop | 9866700 | 0.355191965 |
| 2012 | 101.12 | 3 | 90 | woody_wetland | 33300 | 0.0011987688 |
| 2012 | 101.12 | 3 | 95 | em_herb_wetland | 4500 | 0.0001619958 |
| 2012 | 102.12 | 3 | 11 | open_water | 1008000 | 0.0362518207 |
| 2012 | 102.12 | 3 | 21 | urban_open | 10084500 | 0.3626800453 |
| 2012 | 102.12 | 3 | 22 | urban_low | 11126700 | 0.4001618385 |
| 2012 | 102.12 | 3 | 23 | urban_med | 1674000 | 0.0602039165 |
| 2012 | 102.12 | 3 | 24 | urban_high | 333900 | 0.0120084156 |
| 2012 | 102.12 | 3 | 41 | dec_forest | 2927700 | 0.1052921185 |
| 2012 | 102.12 | 3 | 42 | eg_forest | 45900 | 0.0016507525 |
| 2012 | 102.12 | 3 | 52 | scrub | 4500 | 0.0001618385 |
| 2012 | 102.12 | 3 | 71 | grass_herb | 277200 | 0.0099692507 |
| 2012 | 102.12 | 3 | 81 | pasture_hay | 80100 | 0.002880725 |
| 2012 | 102.12 | 3 | 90 | woody_wetland | 243000 | 0.0087392782 |
| 2012 | 104.12 | 3 | 21 | urban_open | 676800 | 0.0243633772 |
| 2012 | 104.12 | 3 | 22 | urban_low | 112500 | 0.0040497635 |
| 2012 | 104.12 | 3 | 41 | dec_forest | 189000 | 0.0068036027 |
| 2012 | 104.12 | 3 | 71 | grass_herb | 87300 | 0.0031426165 |
| 2012 | 104.12 | 3 | 81 | pasture_hay | 343800 | 0.0123760772 |
| 2012 | 104.12 | 3 | 82 | crop | 26370000 | 0.9492645629 |
| 2012 | 107.12 | 3 | 21 | urban_open | 1618200 | 0.0581971193 |
| 2012 | 107.12 | 3 | 22 | urban_low | 164700 | 0.0059232886 |
| 2012 | 107.12 | 3 | 23 | urban_med | 9900 | 0.0003560447 |
| 2012 | 107.12 | 3 | 41 | dec_forest | 2448900 | 0.0880725036 |
| 2012 | 107.12 | 3 | 43 | mix_forest | 487800 | 0.0175432918 |
| 2012 | 107.12 | 3 | 81 | pasture_hay | 905400 | 0.0325619032 |
| 2012 | 107.12 | 3 | 82 | crop | 22170600 | 0.7973458488 |
| 2012 | 108.12 | 3 | 11 | open_water | 100800 | 0.0036267081 |
| 2012 | 108.12 | 3 | 21 | urban_open | 5212800 | 0.1875526196 |
| 2012 | 108.12 | 3 | 22 | urban_low | 11830500 | 0.4256524836 |
| 2012 | 108.12 | 3 | 23 | urban_med | 6840900 | 0.246130432 |
| 2012 | 108.12 | 3 | 24 | urban_high | 2601000 | 0.0935820219 |
| 2012 | 108.12 | 3 | 41 | dec_forest | 1110600 | 0.0399585519 |
| 2012 | 108.12 | 3 | 71 | grass_herb | 97200 | 0.0034971828 |
| 2012 | 111.12 | 3 | 11 | open_water | 687600 | 0.0247121232 |
| 2012 | 111.12 | 3 | 21 | urban_open | 7997400 | 0.2874239876 |
| 2012 | 111.12 | 3 | 22 | urban_low | 12132000 | 0.4360201837 |
| 2012 | 111.12 | 3 | 23 | urban_med | 3317400 | 0.1192262906 |
| 2012 | 111.12 | 3 | 24 | urban_high | 1391400 | 0.0500064691 |
| 2012 | 111.12 | 3 | 31 | barren | 47700 | 0.0017143227 |
| 2012 | 111.12 | 3 | 41 | dec_forest | 1026000 | 0.0368741105 |
| 2012 | 111.12 | 3 | 42 | eg_forest | 36900 | 0.0013261741 |
| 2012 | 111.12 | 3 | 71 | grass_herb | 231300 | 0.0083128477 |
| 2012 | 111.12 | 3 | 81 | pasture_hay | 522000 | 0.0187605124 |
| 2012 | 111.12 | 3 | 82 | crop | 384300 | 0.0138116186 |
| 2012 | 111.12 | 3 | 90 | woody_wetland | 50400 | 0.0018113598 |
| 2012 | 112.12 | 3 | 11 | open_water | 887400 | 0.0319155823 |
| 2012 | 112.12 | 3 | 21 | urban_open | 9754200 | 0.3508124555 |
| 2012 | 112.12 | 3 | 22 | urban_low | 8262000 | 0.2971450767 |
| 2012 | 112.12 | 3 | 23 | urban_med | 1513800 | 0.0544442287 |
| 2012 | 112.12 | 3 | 24 | urban_high | 1377000 | 0.0495241795 |
| 2012 | 112.12 | 3 | 31 | barren | 65700 | 0.0023629184 |
| 2012 | 112.12 | 3 | 41 | dec_forest | 2970000 | 0.1068168576 |
| 2012 | 112.12 | 3 | 42 | eg_forest | 132300 | 0.0047582055 |
| 2012 | 112.12 | 3 | 43 | mix_forest | 29700 | 0.0010681686 |
| 2012 | 112.12 | 3 | 52 | scrub | 12600 | 0.0004531624 |
| 2012 | 112.12 | 3 | 71 | grass_herb | 278100 | 0.0100019421 |
| 2012 | 112.12 | 3 | 81 | pasture_hay | 925200 | 0.0332750696 |
| 2012 | 112.12 | 3 | 82 | crop | 1459800 | 0.052502104 |
| 2012 | 112.12 | 3 | 90 | woody_wetland | 126900 | 0.004563993 |
| 2012 | 112.12 | 3 | 95 | em_herb_wetland | 9900 | 0.0003560562 |
| 2012 | 113.12 | 3 | 11 | open_water | 138600 | 0.0049852708 |
| 2012 | 113.12 | 3 | 21 | urban_open | 2330100 | 0.0838108187 |
| 2012 | 113.12 | 3 | 22 | urban_low | 14708700 | 0.5290537697 |
| 2012 | 113.12 | 3 | 23 | urban_med | 7495200 | 0.2695930854 |
| 2012 | 113.12 | 3 | 24 | urban_high | 1603800 | 0.0576867049 |
| 2012 | 113.12 | 3 | 31 | barren | 4500 | 0.0001618594 |
| 2012 | 113.12 | 3 | 41 | dec_forest | 1275300 | 0.0458709657 |
| 2012 | 113.12 | 3 | 42 | eg_forest | 24300 | 0.000874041 |
| 2012 | 113.12 | 3 | 71 | grass_herb | 37800 | 0.0013596193 |
| 2012 | 113.12 | 3 | 90 | woody_wetland | 167400 | 0.0060211712 |
| 2012 | 113.12 | 3 | 95 | em_herb_wetland | 16200 | 0.000582694 |
| 2012 | 115.12 | 3 | 11 | open_water | 179100 | 0.0064417972 |
| 2012 | 115.12 | 3 | 21 | urban_open | 6751800 | 0.2428460443 |
| 2012 | 115.12 | 3 | 22 | urban_low | 8779500 | 0.3157775476 |
| 2012 | 115.12 | 3 | 23 | urban_med | 2982600 | 0.1072769649 |
| 2012 | 115.12 | 3 | 24 | urban_high | 812700 | 0.0292308688 |
| 2012 | 115.12 | 3 | 41 | dec_forest | 6554700 | 0.2357568302 |
| 2012 | 115.12 | 3 | 42 | eg_forest | 116100 | 0.0041758384 |
| 2012 | 115.12 | 3 | 52 | scrub | 36900 | 0.0013272045 |
| 2012 | 115.12 | 3 | 71 | grass_herb | 243900 | 0.0087724977 |
| 2012 | 115.12 | 3 | 81 | pasture_hay | 1314900 | 0.0472937977 |
| 2012 | 115.12 | 3 | 90 | woody_wetland | 30600 | 0.0011006086 |
| 2012 | 9.12 | 4 | 11 | open_water | 348300 | 0.0070448174 |
| 2012 | 9.12 | 4 | 21 | urban_open | 14095800 | 0.2851057633 |
| 2012 | 9.12 | 4 | 22 | urban_low | 10715400 | 0.2167328066 |
| 2012 | 9.12 | 4 | 23 | urban_med | 3622500 | 0.0732697419 |
| 2012 | 9.12 | 4 | 24 | urban_high | 1718100 | 0.0347507919 |
| 2012 | 9.12 | 4 | 31 | barren | 112500 | 0.0022754578 |
| 2012 | 9.12 | 4 | 41 | dec_forest | 10002600 | 0.2023155059 |
| 2012 | 9.12 | 4 | 42 | eg_forest | 291600 | 0.0058979867 |
| 2012 | 9.12 | 4 | 43 | mix_forest | 12600 | 0.0002548513 |
| 2012 | 9.12 | 4 | 52 | scrub | 7200 | 0.0001456293 |
| 2012 | 9.12 | 4 | 71 | grass_herb | 1010700 | 0.0204427131 |
| 2012 | 9.12 | 4 | 81 | pasture_hay | 4036500 | 0.0816434267 |
| 2012 | 9.12 | 4 | 82 | crop | 1899900 | 0.0384279317 |
| 2012 | 9.12 | 4 | 90 | woody_wetland | 1562400 | 0.0316015582 |
| 2012 | 9.12 | 4 | 95 | em_herb_wetland | 4500 | 9.10E-05 |
| 2012 | 11.12 | 4 | 11 | open_water | 1763100 | 0.0356648704 |
| 2012 | 11.12 | 4 | 21 | urban_open | 3159000 | 0.0639018351 |
| 2012 | 11.12 | 4 | 22 | urban_low | 3016800 | 0.0610253423 |
| 2012 | 11.12 | 4 | 23 | urban_med | 1038600 | 0.0210093213 |
| 2012 | 11.12 | 4 | 24 | urban_high | 135900 | 0.0027490533 |
| 2012 | 11.12 | 4 | 31 | barren | 14400 | 0.0002912904 |
| 2012 | 11.12 | 4 | 41 | dec_forest | 10148400 | 0.2052869211 |
| 2012 | 11.12 | 4 | 42 | eg_forest | 69300 | 0.0014018351 |
| 2012 | 11.12 | 4 | 43 | mix_forest | 7200 | 0.0001456452 |
| 2012 | 11.12 | 4 | 52 | scrub | 258300 | 0.0052250218 |
| 2012 | 11.12 | 4 | 71 | grass_herb | 92700 | 0.0018751821 |
| 2012 | 11.12 | 4 | 81 | pasture_hay | 5543100 | 0.1121286047 |
| 2012 | 11.12 | 4 | 82 | crop | 24084000 | 0.4871832217 |
| 2012 | 11.12 | 4 | 90 | woody_wetland | 91800 | 0.0018569764 |
| 2012 | 11.12 | 4 | 95 | em_herb_wetland | 12600 | 0.0002548791 |
| 2012 | 12.12 | 4 | 11 | open_water | 581400 | 0.0117662059 |
| 2012 | 12.12 | 4 | 21 | urban_open | 16516800 | 0.3342622443 |
| 2012 | 12.12 | 4 | 22 | urban_low | 16376400 | 0.3314208695 |
| 2012 | 12.12 | 4 | 23 | urban_med | 6041700 | 0.1222701856 |
| 2012 | 12.12 | 4 | 24 | urban_high | 4381200 | 0.0886654645 |
| 2012 | 12.12 | 4 | 41 | dec_forest | 3693600 | 0.0747500137 |
| 2012 | 12.12 | 4 | 42 | eg_forest | 167400 | 0.003387793 |
| 2012 | 12.12 | 4 | 43 | mix_forest | 12600 | 0.0002549952 |
| 2012 | 12.12 | 4 | 52 | scrub | 6300 | 0.0001274976 |
| 2012 | 12.12 | 4 | 71 | grass_herb | 446400 | 0.0090341147 |
| 2012 | 12.12 | 4 | 81 | pasture_hay | 591300 | 0.0119665592 |
| 2012 | 12.12 | 4 | 82 | crop | 465300 | 0.0094166075 |
| 2012 | 12.12 | 4 | 90 | woody_wetland | 126900 | 0.0025681657 |
| 2012 | 12.12 | 4 | 95 | em_herb_wetland | 5400 | 0.0001092836 |
| 2012 | 17.12 | 4 | 11 | open_water | 1143900 | 0.0231410677 |
| 2012 | 17.12 | 4 | 21 | urban_open | 15832800 | 0.3202971379 |
| 2012 | 17.12 | 4 | 22 | urban_low | 11340900 | 0.2294261161 |
| 2012 | 17.12 | 4 | 23 | urban_med | 1126800 | 0.0227951351 |
| 2012 | 17.12 | 4 | 24 | urban_high | 323100 | 0.0065363047 |
| 2012 | 17.12 | 4 | 31 | barren | 65700 | 0.0013291093 |
| 2012 | 17.12 | 4 | 41 | dec_forest | 7305300 | 0.1477860316 |
| 2012 | 17.12 | 4 | 42 | eg_forest | 186300 | 0.0037688442 |
| 2012 | 17.12 | 4 | 43 | mix_forest | 29700 | 0.0006008302 |
| 2012 | 17.12 | 4 | 52 | scrub | 12600 | 0.0002548977 |
| 2012 | 17.12 | 4 | 71 | grass_herb | 610200 | 0.0123443303 |
| 2012 | 17.12 | 4 | 81 | pasture_hay | 4122000 | 0.0833879543 |
| 2012 | 17.12 | 4 | 82 | crop | 7016400 | 0.141941592 |
| 2012 | 17.12 | 4 | 90 | woody_wetland | 276300 | 0.0055895419 |
| 2012 | 17.12 | 4 | 95 | em_herb_wetland | 39600 | 0.000801107 |
| 2012 | 20.12 | 4 | 11 | open_water | 1160100 | 0.0234653754 |
| 2012 | 20.12 | 4 | 21 | urban_open | 1735200 | 0.0350979393 |
| 2012 | 20.12 | 4 | 22 | urban_low | 580500 | 0.0117417898 |
| 2012 | 20.12 | 4 | 23 | urban_med | 67500 | 0.0013653244 |
| 2012 | 20.12 | 4 | 24 | urban_high | 37800 | 0.0007645817 |
| 2012 | 20.12 | 4 | 41 | dec_forest | 3938400 | 0.0796621277 |
| 2012 | 20.12 | 4 | 42 | eg_forest | 22500 | 0.0004551081 |
| 2012 | 20.12 | 4 | 43 | mix_forest | 57600 | 0.0011650768 |
| 2012 | 20.12 | 4 | 52 | scrub | 7200 | 0.0001456346 |
| 2012 | 20.12 | 4 | 81 | pasture_hay | 2977200 | 0.0602199083 |
| 2012 | 20.12 | 4 | 82 | crop | 38637900 | 0.7815298915 |
| 2012 | 20.12 | 4 | 90 | woody_wetland | 123300 | 0.0024939926 |
| 2012 | 20.12 | 4 | 95 | em_herb_wetland | 93600 | 0.0018932498 |
| 2012 | 21.12 | 4 | 11 | open_water | 580500 | 0.0117366621 |
| 2012 | 21.12 | 4 | 21 | urban_open | 16132500 | 0.3261700269 |
| 2012 | 21.12 | 4 | 22 | urban_low | 8921700 | 0.1803806682 |
| 2012 | 21.12 | 4 | 23 | urban_med | 4409100 | 0.0891440425 |
| 2012 | 21.12 | 4 | 24 | urban_high | 1088100 | 0.0219994177 |
| 2012 | 21.12 | 4 | 41 | dec_forest | 15126300 | 0.3058264794 |
| 2012 | 21.12 | 4 | 42 | eg_forest | 942300 | 0.0190516049 |
| 2012 | 21.12 | 4 | 43 | mix_forest | 18000 | 0.0003639275 |
| 2012 | 21.12 | 4 | 52 | scrub | 136800 | 0.002765849 |
| 2012 | 21.12 | 4 | 71 | grass_herb | 330300 | 0.0066780697 |
| 2012 | 21.12 | 4 | 81 | pasture_hay | 1292400 | 0.0261299949 |
| 2012 | 21.12 | 4 | 82 | crop | 408600 | 0.0082611544 |
| 2012 | 21.12 | 4 | 90 | woody_wetland | 20700 | 0.0004185166 |
| 2012 | 21.12 | 4 | 95 | em_herb_wetland | 53100 | 0.0010735861 |
| 2012 | 26.12 | 4 | 11 | open_water | 17100 | 0.000346109 |
| 2012 | 26.12 | 4 | 21 | urban_open | 3928500 | 0.0795139901 |
| 2012 | 26.12 | 4 | 22 | urban_low | 1121400 | 0.0226974643 |
| 2012 | 26.12 | 4 | 23 | urban_med | 144900 | 0.0029328184 |
| 2012 | 26.12 | 4 | 24 | urban_high | 35100 | 0.0007104343 |
| 2012 | 26.12 | 4 | 31 | barren | 277200 | 0.0056106092 |
| 2012 | 26.12 | 4 | 41 | dec_forest | 24730200 | 0.5005464879 |
| 2012 | 26.12 | 4 | 42 | eg_forest | 327600 | 0.0066307199 |
| 2012 | 26.12 | 4 | 52 | scrub | 19800 | 0.0004007578 |
| 2012 | 26.12 | 4 | 71 | grass_herb | 306000 | 0.0061935296 |
| 2012 | 26.12 | 4 | 81 | pasture_hay | 16634700 | 0.3366911979 |
| 2012 | 26.12 | 4 | 82 | crop | 1859400 | 0.0376348003 |
| 2012 | 26.12 | 4 | 90 | woody_wetland | 4500 | 9.11E-05 |
| 2012 | 27.12 | 4 | 11 | open_water | 151200 | 0.0030566573 |
| 2012 | 27.12 | 4 | 21 | urban_open | 3082500 | 0.0623157818 |
| 2012 | 27.12 | 4 | 22 | urban_low | 1575000 | 0.0318401805 |
| 2012 | 27.12 | 4 | 23 | urban_med | 247500 | 0.0050034569 |
| 2012 | 27.12 | 4 | 24 | urban_high | 58500 | 0.0011826353 |
| 2012 | 27.12 | 4 | 31 | barren | 57600 | 0.0011644409 |
| 2012 | 27.12 | 4 | 41 | dec_forest | 7131600 | 0.1441723373 |
| 2012 | 27.12 | 4 | 42 | eg_forest | 921600 | 0.0186310542 |
| 2012 | 27.12 | 4 | 43 | mix_forest | 4500 | 9.10E-05 |
| 2012 | 27.12 | 4 | 71 | grass_herb | 1081800 | 0.0218696554 |
| 2012 | 27.12 | 4 | 81 | pasture_hay | 2629800 | 0.0531640042 |
| 2012 | 27.12 | 4 | 82 | crop | 32402700 | 0.6550525818 |
| 2012 | 27.12 | 4 | 90 | woody_wetland | 109800 | 0.0022197154 |
| 2012 | 27.12 | 4 | 95 | em_herb_wetland | 11700 | 0.0002365271 |
| 2012 | 28.12 | 4 | 11 | open_water | 108900 | 0.0022023224 |
| 2012 | 28.12 | 4 | 21 | urban_open | 7901100 | 0.1597866841 |
| 2012 | 28.12 | 4 | 22 | urban_low | 4439700 | 0.0897855921 |
| 2012 | 28.12 | 4 | 23 | urban_med | 903600 | 0.018273816 |
| 2012 | 28.12 | 4 | 24 | urban_high | 552600 | 0.0111754214 |
| 2012 | 28.12 | 4 | 31 | barren | 169200 | 0.0034217903 |
| 2012 | 28.12 | 4 | 41 | dec_forest | 20426400 | 0.4130901678 |
| 2012 | 28.12 | 4 | 42 | eg_forest | 367200 | 0.0074260129 |
| 2012 | 28.12 | 4 | 71 | grass_herb | 1823400 | 0.0368752503 |
| 2012 | 28.12 | 4 | 81 | pasture_hay | 5613300 | 0.1135197117 |
| 2012 | 28.12 | 4 | 82 | crop | 6534900 | 0.132157548 |
| 2012 | 28.12 | 4 | 90 | woody_wetland | 583200 | 0.0117942558 |
| 2012 | 28.12 | 4 | 95 | em_herb_wetland | 24300 | 0.0004914273 |
| 2012 | 30.12 | 4 | 11 | open_water | 110700 | 0.002237747 |
| 2012 | 30.12 | 4 | 21 | urban_open | 12071700 | 0.2440235782 |
| 2012 | 30.12 | 4 | 22 | urban_low | 13814100 | 0.2792453517 |
| 2012 | 30.12 | 4 | 23 | urban_med | 4790700 | 0.096841684 |
| 2012 | 30.12 | 4 | 24 | urban_high | 1278000 | 0.025834152 |
| 2012 | 30.12 | 4 | 41 | dec_forest | 8376300 | 0.1693228541 |
| 2012 | 30.12 | 4 | 42 | eg_forest | 276300 | 0.0055852709 |
| 2012 | 30.12 | 4 | 52 | scrub | 189900 | 0.0038387367 |
| 2012 | 30.12 | 4 | 71 | grass_herb | 383400 | 0.0077502456 |
| 2012 | 30.12 | 4 | 81 | pasture_hay | 2703600 | 0.0546519667 |
| 2012 | 30.12 | 4 | 82 | crop | 5283000 | 0.1067932904 |
| 2012 | 30.12 | 4 | 90 | woody_wetland | 180000 | 0.003638613 |
| 2012 | 30.12 | 4 | 95 | em_herb_wetland | 11700 | 0.0002365098 |
| 2012 | 33.12 | 4 | 11 | open_water | 181800 | 0.0036782781 |
| 2012 | 33.12 | 4 | 21 | urban_open | 14230800 | 0.2879254147 |
| 2012 | 33.12 | 4 | 22 | urban_low | 11454300 | 0.2317497314 |
| 2012 | 33.12 | 4 | 23 | urban_med | 2003400 | 0.0405338966 |
| 2012 | 33.12 | 4 | 24 | urban_high | 674100 | 0.013638764 |
| 2012 | 33.12 | 4 | 41 | dec_forest | 6222600 | 0.1258990841 |
| 2012 | 33.12 | 4 | 42 | eg_forest | 72900 | 0.0014749531 |
| 2012 | 33.12 | 4 | 52 | scrub | 189000 | 0.0038239525 |
| 2012 | 33.12 | 4 | 71 | grass_herb | 632700 | 0.0128011363 |
| 2012 | 33.12 | 4 | 81 | pasture_hay | 3351600 | 0.0678114245 |
| 2012 | 33.12 | 4 | 82 | crop | 10162800 | 0.2056193893 |
| 2012 | 33.12 | 4 | 90 | woody_wetland | 228600 | 0.0046251616 |
| 2012 | 33.12 | 4 | 95 | em_herb_wetland | 20700 | 0.0004188138 |
| 2012 | 36.12 | 4 | 11 | open_water | 353700 | 0.0071527373 |
| 2012 | 36.12 | 4 | 21 | urban_open | 7774200 | 0.1572146185 |
| 2012 | 36.12 | 4 | 22 | urban_low | 5058000 | 0.1022859639 |
| 2012 | 36.12 | 4 | 23 | urban_med | 796500 | 0.0161073093 |
| 2012 | 36.12 | 4 | 24 | urban_high | 177300 | 0.0035854688 |
| 2012 | 36.12 | 4 | 31 | barren | 8100 | 0.0001638031 |
| 2012 | 36.12 | 4 | 41 | dec_forest | 6318900 | 0.1277846535 |
| 2012 | 36.12 | 4 | 42 | eg_forest | 136800 | 0.0027664531 |
| 2012 | 36.12 | 4 | 43 | mix_forest | 81000 | 0.0016380315 |
| 2012 | 36.12 | 4 | 71 | grass_herb | 117900 | 0.0023842458 |
| 2012 | 36.12 | 4 | 81 | pasture_hay | 12591900 | 0.2546410891 |
| 2012 | 36.12 | 4 | 82 | crop | 15988500 | 0.3233292079 |
| 2012 | 36.12 | 4 | 95 | em_herb_wetland | 46800 | 0.0009464182 |
| 2012 | 40.12 | 4 | 11 | open_water | 130500 | 0.0026399155 |
| 2012 | 40.12 | 4 | 21 | urban_open | 2420100 | 0.0489567782 |
| 2012 | 40.12 | 4 | 22 | urban_low | 33300 | 0.0006736336 |
| 2012 | 40.12 | 4 | 23 | urban_med | 8100 | 0.0001638568 |
| 2012 | 40.12 | 4 | 41 | dec_forest | 30369600 | 0.6143538579 |
| 2012 | 40.12 | 4 | 42 | eg_forest | 1102500 | 0.0223027346 |
| 2012 | 40.12 | 4 | 43 | mix_forest | 4500 | 9.10E-05 |
| 2012 | 40.12 | 4 | 52 | scrub | 7200 | 0.0001456505 |
| 2012 | 40.12 | 4 | 71 | grass_herb | 464400 | 0.009394458 |
| 2012 | 40.12 | 4 | 81 | pasture_hay | 6761700 | 0.1367840367 |
| 2012 | 40.12 | 4 | 82 | crop | 8061300 | 0.163073954 |
| 2012 | 40.12 | 4 | 90 | woody_wetland | 65700 | 0.0013290609 |
| 2012 | 40.12 | 4 | 95 | em_herb_wetland | 4500 | 9.10E-05 |
| 2012 | 41.12 | 4 | 11 | open_water | 324000 | 0.0065536764 |
| 2012 | 41.12 | 4 | 21 | urban_open | 8084700 | 0.1635324316 |
| 2012 | 41.12 | 4 | 22 | urban_low | 19536300 | 0.3951684841 |
| 2012 | 41.12 | 4 | 23 | urban_med | 15109200 | 0.3056197775 |
| 2012 | 41.12 | 4 | 24 | urban_high | 5126400 | 0.1036937249 |
| 2012 | 41.12 | 4 | 41 | dec_forest | 1178100 | 0.0238298957 |
| 2012 | 41.12 | 4 | 71 | grass_herb | 79200 | 0.0016020098 |
| 2012 | 42.12 | 4 | 11 | open_water | 12600 | 0.0002547446 |
| 2012 | 42.12 | 4 | 21 | urban_open | 2466000 | 0.0498571611 |
| 2012 | 42.12 | 4 | 22 | urban_low | 9900 | 0.0002001565 |
| 2012 | 42.12 | 4 | 41 | dec_forest | 40599900 | 0.820841749 |
| 2012 | 42.12 | 4 | 42 | eg_forest | 2276100 | 0.0460177957 |
| 2012 | 42.12 | 4 | 52 | scrub | 107100 | 0.0021653293 |
| 2012 | 42.12 | 4 | 71 | grass_herb | 92700 | 0.0018741926 |
| 2012 | 42.12 | 4 | 81 | pasture_hay | 2446200 | 0.0494568481 |
| 2012 | 42.12 | 4 | 82 | crop | 1450800 | 0.0293320232 |
| 2012 | 43.12 | 4 | 11 | open_water | 1252800 | 0.0253404209 |
| 2012 | 43.12 | 4 | 21 | urban_open | 1927800 | 0.0389936649 |
| 2012 | 43.12 | 4 | 22 | urban_low | 767700 | 0.0155282895 |
| 2012 | 43.12 | 4 | 23 | urban_med | 63900 | 0.0012925071 |
| 2012 | 43.12 | 4 | 24 | urban_high | 23400 | 0.0004733125 |
| 2012 | 43.12 | 4 | 31 | barren | 18900 | 0.0003822908 |
| 2012 | 43.12 | 4 | 41 | dec_forest | 6614100 | 0.133783587 |
| 2012 | 43.12 | 4 | 42 | eg_forest | 30600 | 0.0006189471 |
| 2012 | 43.12 | 4 | 43 | mix_forest | 27000 | 0.0005461298 |
| 2012 | 43.12 | 4 | 52 | scrub | 12600 | 0.0002548606 |
| 2012 | 43.12 | 4 | 71 | grass_herb | 29700 | 0.0006007427 |
| 2012 | 43.12 | 4 | 81 | pasture_hay | 4176900 | 0.0844862739 |
| 2012 | 43.12 | 4 | 82 | crop | 34398000 | 0.6957693148 |
| 2012 | 43.12 | 4 | 90 | woody_wetland | 90000 | 0.0018204325 |
| 2012 | 43.12 | 4 | 95 | em_herb_wetland | 5400 | 0.000109226 |
| 2012 | 44.12 | 4 | 11 | open_water | 545400 | 0.011033428 |
| 2012 | 44.12 | 4 | 21 | urban_open | 1956600 | 0.0395819678 |
| 2012 | 44.12 | 4 | 22 | urban_low | 689400 | 0.0139465443 |
| 2012 | 44.12 | 4 | 23 | urban_med | 4500 | 9.10E-05 |
| 2012 | 44.12 | 4 | 41 | dec_forest | 27578700 | 0.5579163936 |
| 2012 | 44.12 | 4 | 42 | eg_forest | 296100 | 0.0059900954 |
| 2012 | 44.12 | 4 | 52 | scrub | 1592100 | 0.0322081422 |
| 2012 | 44.12 | 4 | 71 | grass_herb | 1685700 | 0.0341016678 |
| 2012 | 44.12 | 4 | 81 | pasture_hay | 2716200 | 0.0549486563 |
| 2012 | 44.12 | 4 | 82 | crop | 10041300 | 0.2031352414 |
| 2012 | 44.12 | 4 | 90 | woody_wetland | 2300400 | 0.046537033 |
| 2012 | 44.12 | 4 | 95 | em_herb_wetland | 25200 | 0.0005097954 |
| 2012 | 47.12 | 4 | 11 | open_water | 112500 | 0.0022774478 |
| 2012 | 47.12 | 4 | 21 | urban_open | 10161900 | 0.205717305 |
| 2012 | 47.12 | 4 | 22 | urban_low | 15392700 | 0.3116095179 |
| 2012 | 47.12 | 4 | 23 | urban_med | 10000800 | 0.2024559997 |
| 2012 | 47.12 | 4 | 24 | urban_high | 6472800 | 0.1310352367 |
| 2012 | 47.12 | 4 | 41 | dec_forest | 747000 | 0.0151222534 |
| 2012 | 47.12 | 4 | 42 | eg_forest | 11700 | 0.0002368546 |
| 2012 | 47.12 | 4 | 43 | mix_forest | 7200 | 0.0001457567 |
| 2012 | 47.12 | 4 | 71 | grass_herb | 192600 | 0.0038989906 |
| 2012 | 47.12 | 4 | 81 | pasture_hay | 1173600 | 0.0237583355 |
| 2012 | 47.12 | 4 | 82 | crop | 5120100 | 0.1036512043 |
| 2012 | 47.12 | 4 | 95 | em_herb_wetland | 4500 | 9.11E-05 |
| 2012 | 50.12 | 4 | 11 | open_water | 4798800 | 0.0970849038 |
| 2012 | 50.12 | 4 | 21 | urban_open | 4280400 | 0.0865971122 |
| 2012 | 50.12 | 4 | 22 | urban_low | 1025100 | 0.0207388795 |
| 2012 | 50.12 | 4 | 23 | urban_med | 850500 | 0.017206533 |
| 2012 | 50.12 | 4 | 24 | urban_high | 507600 | 0.0102692959 |
| 2012 | 50.12 | 4 | 31 | barren | 1222200 | 0.0247264252 |
| 2012 | 50.12 | 4 | 41 | dec_forest | 31006800 | 0.6273010324 |
| 2012 | 50.12 | 4 | 42 | eg_forest | 213300 | 0.0043152892 |
| 2012 | 50.12 | 4 | 43 | mix_forest | 5400 | 0.0001092478 |
| 2012 | 50.12 | 4 | 52 | scrub | 4500 | 9.10E-05 |
| 2012 | 50.12 | 4 | 71 | grass_herb | 990900 | 0.0200469766 |
| 2012 | 50.12 | 4 | 81 | pasture_hay | 4197600 | 0.0849219788 |
| 2012 | 50.12 | 4 | 82 | crop | 294300 | 0.0059540067 |
| 2012 | 50.12 | 4 | 90 | woody_wetland | 22500 | 0.0004551993 |
| 2012 | 50.12 | 4 | 95 | em_herb_wetland | 9000 | 0.0001820797 |
| 2012 | 52.12 | 4 | 11 | open_water | 58500 | 0.0011830873 |
| 2012 | 52.12 | 4 | 21 | urban_open | 3486600 | 0.0705120038 |
| 2012 | 52.12 | 4 | 22 | urban_low | 191700 | 0.0038768861 |
| 2012 | 52.12 | 4 | 23 | urban_med | 27000 | 0.0005460403 |
| 2012 | 52.12 | 4 | 24 | urban_high | 4500 | 9.10E-05 |
| 2012 | 52.12 | 4 | 31 | barren | 27000 | 0.0005460403 |
| 2012 | 52.12 | 4 | 41 | dec_forest | 27435600 | 0.5548497479 |
| 2012 | 52.12 | 4 | 42 | eg_forest | 455400 | 0.0092098797 |
| 2012 | 52.12 | 4 | 52 | scrub | 62100 | 0.0012558927 |
| 2012 | 52.12 | 4 | 71 | grass_herb | 223200 | 0.0045139331 |
| 2012 | 52.12 | 4 | 81 | pasture_hay | 8118000 | 0.1641761162 |
| 2012 | 52.12 | 4 | 82 | crop | 9246600 | 0.1870006006 |
| 2012 | 52.12 | 4 | 90 | woody_wetland | 106200 | 0.0021477585 |
| 2012 | 52.12 | 4 | 95 | em_herb_wetland | 4500 | 9.10E-05 |
| 2012 | 53.12 | 4 | 11 | open_water | 188100 | 0.0038060897 |
| 2012 | 53.12 | 4 | 21 | urban_open | 5293800 | 0.1071168415 |
| 2012 | 53.12 | 4 | 22 | urban_low | 3250800 | 0.065777972 |
| 2012 | 53.12 | 4 | 23 | urban_med | 676800 | 0.0136946387 |
| 2012 | 53.12 | 4 | 24 | urban_high | 66600 | 0.0013476107 |
| 2012 | 53.12 | 4 | 41 | dec_forest | 10859400 | 0.2197333916 |
| 2012 | 53.12 | 4 | 42 | eg_forest | 423900 | 0.0085773601 |
| 2012 | 53.12 | 4 | 43 | mix_forest | 18900 | 0.0003824301 |
| 2012 | 53.12 | 4 | 52 | scrub | 342900 | 0.0069383741 |
| 2012 | 53.12 | 4 | 71 | grass_herb | 1308600 | 0.0264787296 |
| 2012 | 53.12 | 4 | 81 | pasture_hay | 19221300 | 0.3889313811 |
| 2012 | 53.12 | 4 | 82 | crop | 7752600 | 0.1568691725 |
| 2012 | 53.12 | 4 | 90 | woody_wetland | 8100 | 0.0001638986 |
| 2012 | 53.12 | 4 | 95 | em_herb_wetland | 9000 | 0.0001821096 |
| 2012 | 54.12 | 4 | 11 | open_water | 125100 | 0.0025292501 |
| 2012 | 54.12 | 4 | 21 | urban_open | 5255100 | 0.106246702 |
| 2012 | 54.12 | 4 | 22 | urban_low | 4653900 | 0.0940917445 |
| 2012 | 54.12 | 4 | 23 | urban_med | 1053000 | 0.0212893717 |
| 2012 | 54.12 | 4 | 24 | urban_high | 144900 | 0.0029295631 |
| 2012 | 54.12 | 4 | 41 | dec_forest | 6822900 | 0.1379442109 |
| 2012 | 54.12 | 4 | 42 | eg_forest | 31500 | 0.0006368615 |
| 2012 | 54.12 | 4 | 43 | mix_forest | 54900 | 0.0011099587 |
| 2012 | 54.12 | 4 | 71 | grass_herb | 785700 | 0.0158851466 |
| 2012 | 54.12 | 4 | 81 | pasture_hay | 4419000 | 0.0893425769 |
| 2012 | 54.12 | 4 | 82 | crop | 26091900 | 0.5275215168 |
| 2012 | 54.12 | 4 | 95 | em_herb_wetland | 23400 | 0.0004730971 |
| 2012 | 101.12 | 4 | 11 | open_water | 468900 | 0.0094880807 |
| 2012 | 101.12 | 4 | 21 | urban_open | 7414200 | 0.1500245852 |
| 2012 | 101.12 | 4 | 22 | urban_low | 5385600 | 0.1089763435 |
| 2012 | 101.12 | 4 | 23 | urban_med | 3036600 | 0.0614448835 |
| 2012 | 101.12 | 4 | 24 | urban_high | 1881900 | 0.0380798019 |
| 2012 | 101.12 | 4 | 31 | barren | 62100 | 0.0012565788 |
| 2012 | 101.12 | 4 | 41 | dec_forest | 7492500 | 0.1516089672 |
| 2012 | 101.12 | 4 | 42 | eg_forest | 246600 | 0.0049898927 |
| 2012 | 101.12 | 4 | 43 | mix_forest | 6300 | 0.000127479 |
| 2012 | 101.12 | 4 | 52 | scrub | 9000 | 0.0001821129 |
| 2012 | 101.12 | 4 | 71 | grass_herb | 477900 | 0.0096701936 |
| 2012 | 101.12 | 4 | 81 | pasture_hay | 4572000 | 0.0925133398 |
| 2012 | 101.12 | 4 | 82 | crop | 17844300 | 0.3610751944 |
| 2012 | 101.12 | 4 | 90 | woody_wetland | 510300 | 0.0103257999 |
| 2012 | 101.12 | 4 | 95 | em_herb_wetland | 11700 | 0.0002367467 |
| 2012 | 102.12 | 4 | 11 | open_water | 1215000 | 0.0245941958 |
| 2012 | 102.12 | 4 | 21 | urban_open | 17298900 | 0.350166694 |
| 2012 | 102.12 | 4 | 22 | urban_low | 19006200 | 0.3847260935 |
| 2012 | 102.12 | 4 | 23 | urban_med | 3358800 | 0.0679892879 |
| 2012 | 102.12 | 4 | 24 | urban_high | 904500 | 0.0183090124 |
| 2012 | 102.12 | 4 | 31 | barren | 38700 | 0.0007833707 |
| 2012 | 102.12 | 4 | 41 | dec_forest | 5650200 | 0.1143721193 |
| 2012 | 102.12 | 4 | 42 | eg_forest | 85500 | 0.0017307027 |
| 2012 | 102.12 | 4 | 52 | scrub | 33300 | 0.0006740631 |
| 2012 | 102.12 | 4 | 71 | grass_herb | 499500 | 0.0101109471 |
| 2012 | 102.12 | 4 | 81 | pasture_hay | 740700 | 0.0149933505 |
| 2012 | 102.12 | 4 | 82 | crop | 34200 | 0.0006922811 |
| 2012 | 102.12 | 4 | 90 | woody_wetland | 536400 | 0.010857882 |
| 2012 | 104.12 | 4 | 21 | urban_open | 1537200 | 0.0310963842 |
| 2012 | 104.12 | 4 | 22 | urban_low | 400500 | 0.0081018097 |
| 2012 | 104.12 | 4 | 41 | dec_forest | 360900 | 0.0073007319 |
| 2012 | 104.12 | 4 | 71 | grass_herb | 258300 | 0.0052252121 |
| 2012 | 104.12 | 4 | 81 | pasture_hay | 1251900 | 0.0253249827 |
| 2012 | 104.12 | 4 | 82 | crop | 45624600 | 0.9229508794 |
| 2012 | 107.12 | 4 | 21 | urban_open | 2593800 | 0.0524639106 |
| 2012 | 107.12 | 4 | 22 | urban_low | 228600 | 0.0046238145 |
| 2012 | 107.12 | 4 | 23 | urban_med | 15300 | 0.0003094679 |
| 2012 | 107.12 | 4 | 41 | dec_forest | 5284800 | 0.1068938525 |
| 2012 | 107.12 | 4 | 43 | mix_forest | 998100 | 0.0201882293 |
| 2012 | 107.12 | 4 | 81 | pasture_hay | 1629900 | 0.0329674331 |
| 2012 | 107.12 | 4 | 82 | crop | 38689200 | 0.7825532922 |
| 2012 | 108.12 | 4 | 11 | open_water | 458100 | 0.0092643151 |
| 2012 | 108.12 | 4 | 21 | urban_open | 8217000 | 0.1661752393 |
| 2012 | 108.12 | 4 | 22 | urban_low | 20549700 | 0.4155837065 |
| 2012 | 108.12 | 4 | 23 | urban_med | 13131900 | 0.2655709657 |
| 2012 | 108.12 | 4 | 24 | urban_high | 4664700 | 0.0943358451 |
| 2012 | 108.12 | 4 | 41 | dec_forest | 2319300 | 0.0469040079 |
| 2012 | 108.12 | 4 | 71 | grass_herb | 107100 | 0.0021659204 |
| 2012 | 111.12 | 4 | 11 | open_water | 708300 | 0.0143375052 |
| 2012 | 111.12 | 4 | 21 | urban_open | 14211900 | 0.2876792188 |
| 2012 | 111.12 | 4 | 22 | urban_low | 19720800 | 0.3991911242 |
| 2012 | 111.12 | 4 | 23 | urban_med | 7155900 | 0.1448507041 |
| 2012 | 111.12 | 4 | 24 | urban_high | 2844900 | 0.0575868539 |
| 2012 | 111.12 | 4 | 31 | barren | 66600 | 0.0013481263 |
| 2012 | 111.12 | 4 | 41 | dec_forest | 2047500 | 0.0414457744 |
| 2012 | 111.12 | 4 | 42 | eg_forest | 112500 | 0.0022772403 |
| 2012 | 111.12 | 4 | 71 | grass_herb | 344700 | 0.0069774644 |
| 2012 | 111.12 | 4 | 81 | pasture_hay | 1278900 | 0.0258876683 |
| 2012 | 111.12 | 4 | 82 | crop | 859500 | 0.0173981163 |
| 2012 | 111.12 | 4 | 90 | woody_wetland | 50400 | 0.0010202037 |
| 2012 | 112.12 | 4 | 11 | open_water | 1269900 | 0.0256905122 |
| 2012 | 112.12 | 4 | 21 | urban_open | 15120000 | 0.3058827813 |
| 2012 | 112.12 | 4 | 22 | urban_low | 11344500 | 0.2295031226 |
| 2012 | 112.12 | 4 | 23 | urban_med | 3388500 | 0.0685505162 |
| 2012 | 112.12 | 4 | 24 | urban_high | 3294000 | 0.0666387488 |
| 2012 | 112.12 | 4 | 31 | barren | 65700 | 0.0013291335 |
| 2012 | 112.12 | 4 | 41 | dec_forest | 6786900 | 0.1373013127 |
| 2012 | 112.12 | 4 | 42 | eg_forest | 218700 | 0.0044243759 |
| 2012 | 112.12 | 4 | 43 | mix_forest | 29700 | 0.0006008412 |
| 2012 | 112.12 | 4 | 52 | scrub | 18900 | 0.0003823535 |
| 2012 | 112.12 | 4 | 71 | grass_herb | 607500 | 0.0122899332 |
| 2012 | 112.12 | 4 | 81 | pasture_hay | 2499300 | 0.0505616955 |
| 2012 | 112.12 | 4 | 82 | crop | 4527900 | 0.0916009686 |
| 2012 | 112.12 | 4 | 90 | woody_wetland | 224100 | 0.0045336198 |
| 2012 | 112.12 | 4 | 95 | em_herb_wetland | 35100 | 0.000710085 |
| 2012 | 113.12 | 4 | 11 | open_water | 408600 | 0.0082662685 |
| 2012 | 113.12 | 4 | 21 | urban_open | 5271300 | 0.106642147 |
| 2012 | 113.12 | 4 | 22 | urban_low | 23175900 | 0.4688649357 |
| 2012 | 113.12 | 4 | 23 | urban_med | 13355100 | 0.2701831689 |
| 2012 | 113.12 | 4 | 24 | urban_high | 3298500 | 0.0667310003 |
| 2012 | 113.12 | 4 | 31 | barren | 4500 | 9.10E-05 |
| 2012 | 113.12 | 4 | 41 | dec_forest | 3335400 | 0.0674775136 |
| 2012 | 113.12 | 4 | 42 | eg_forest | 62100 | 0.0012563272 |
| 2012 | 113.12 | 4 | 43 | mix_forest | 4500 | 9.10E-05 |
| 2012 | 113.12 | 4 | 71 | grass_herb | 139500 | 0.0028221842 |
| 2012 | 113.12 | 4 | 90 | woody_wetland | 351000 | 0.0071009796 |
| 2012 | 113.12 | 4 | 95 | em_herb_wetland | 23400 | 0.0004733986 |
| 2012 | 115.12 | 4 | 11 | open_water | 326700 | 0.0066094936 |
| 2012 | 115.12 | 4 | 21 | urban_open | 13887000 | 0.2809489995 |
| 2012 | 115.12 | 4 | 22 | urban_low | 13558500 | 0.2743030899 |
| 2012 | 115.12 | 4 | 23 | urban_med | 5997600 | 0.1213379217 |
| 2012 | 115.12 | 4 | 24 | urban_high | 2700000 | 0.0546239143 |
| 2012 | 115.12 | 4 | 41 | dec_forest | 9704700 | 0.1963365561 |
| 2012 | 115.12 | 4 | 42 | eg_forest | 204300 | 0.0041332095 |
| 2012 | 115.12 | 4 | 52 | scrub | 124200 | 0.0025127001 |
| 2012 | 115.12 | 4 | 71 | grass_herb | 401400 | 0.0081207553 |
| 2012 | 115.12 | 4 | 81 | pasture_hay | 2340000 | 0.0473407258 |
| 2012 | 115.12 | 4 | 82 | crop | 128700 | 0.0026037399 |
| 2012 | 115.12 | 4 | 90 | woody_wetland | 51300 | 0.0010378544 |
| 2012 | 115.12 | 4 | 95 | em_herb_wetland | 4500 | 9.10E-05 |
| 2012 | 9.12 | 5 | 11 | open_water | 756000 | 0.0097886126 |
| 2012 | 9.12 | 5 | 21 | urban_open | 20891700 | 0.2705036474 |
| 2012 | 9.12 | 5 | 22 | urban_low | 14651100 | 0.1897009812 |
| 2012 | 9.12 | 5 | 23 | urban_med | 4299300 | 0.0556669075 |
| 2012 | 9.12 | 5 | 24 | urban_high | 2115000 | 0.027384809 |
| 2012 | 9.12 | 5 | 31 | barren | 135900 | 0.0017596196 |
| 2012 | 9.12 | 5 | 41 | dec_forest | 18252000 | 0.2363250752 |
| 2012 | 9.12 | 5 | 42 | eg_forest | 507600 | 0.0065723542 |
| 2012 | 9.12 | 5 | 43 | mix_forest | 12600 | 0.0001631435 |
| 2012 | 9.12 | 5 | 52 | scrub | 7200 | 9.32E-05 |
| 2012 | 9.12 | 5 | 71 | grass_herb | 1480500 | 0.0191693663 |
| 2012 | 9.12 | 5 | 81 | pasture_hay | 6968700 | 0.0902300324 |
| 2012 | 9.12 | 5 | 82 | crop | 4783500 | 0.0619362808 |
| 2012 | 9.12 | 5 | 90 | woody_wetland | 2359800 | 0.030554455 |
| 2012 | 9.12 | 5 | 95 | em_herb_wetland | 11700 | 0.0001514904 |
| 2012 | 11.12 | 5 | 11 | open_water | 2358000 | 0.0305318603 |
| 2012 | 11.12 | 5 | 21 | urban_open | 4760100 | 0.0616347364 |
| 2012 | 11.12 | 5 | 22 | urban_low | 5819400 | 0.0753507668 |
| 2012 | 11.12 | 5 | 23 | urban_med | 2649600 | 0.0343075561 |
| 2012 | 11.12 | 5 | 24 | urban_high | 568800 | 0.0073649373 |
| 2012 | 11.12 | 5 | 31 | barren | 24300 | 0.0003146413 |
| 2012 | 11.12 | 5 | 41 | dec_forest | 14035500 | 0.1817344893 |
| 2012 | 11.12 | 5 | 42 | eg_forest | 69300 | 0.0008973104 |
| 2012 | 11.12 | 5 | 43 | mix_forest | 7200 | 9.32E-05 |
| 2012 | 11.12 | 5 | 52 | scrub | 333000 | 0.0043117513 |
| 2012 | 11.12 | 5 | 71 | grass_herb | 311400 | 0.0040320701 |
| 2012 | 11.12 | 5 | 81 | pasture_hay | 8089200 | 0.1047405957 |
| 2012 | 11.12 | 5 | 82 | crop | 38043000 | 0.4925884492 |
| 2012 | 11.12 | 5 | 90 | woody_wetland | 149400 | 0.0019344614 |
| 2012 | 11.12 | 5 | 95 | em_herb_wetland | 12600 | 0.0001631473 |
| 2012 | 12.12 | 5 | 11 | open_water | 882000 | 0.0114144614 |
| 2012 | 12.12 | 5 | 21 | urban_open | 27469800 | 0.3555022363 |
| 2012 | 12.12 | 5 | 22 | urban_low | 25299900 | 0.3274203317 |
| 2012 | 12.12 | 5 | 23 | urban_med | 7695000 | 0.0995853522 |
| 2012 | 12.12 | 5 | 24 | urban_high | 5124600 | 0.0663203504 |
| 2012 | 12.12 | 5 | 41 | dec_forest | 6058800 | 0.0784103615 |
| 2012 | 12.12 | 5 | 42 | eg_forest | 274500 | 0.0035524599 |
| 2012 | 12.12 | 5 | 43 | mix_forest | 25200 | 0.0003261275 |
| 2012 | 12.12 | 5 | 52 | scrub | 6300 | 8.15E-05 |
| 2012 | 12.12 | 5 | 71 | grass_herb | 728100 | 0.0094227544 |
| 2012 | 12.12 | 5 | 81 | pasture_hay | 1392300 | 0.0180185427 |
| 2012 | 12.12 | 5 | 82 | crop | 2050200 | 0.0265327991 |
| 2012 | 12.12 | 5 | 90 | woody_wetland | 253800 | 0.0032845695 |
| 2012 | 12.12 | 5 | 95 | em_herb_wetland | 9900 | 0.0001281215 |
| 2012 | 17.12 | 5 | 11 | open_water | 1661400 | 0.0215136471 |
| 2012 | 17.12 | 5 | 21 | urban_open | 23695200 | 0.3068316901 |
| 2012 | 17.12 | 5 | 22 | urban_low | 18114300 | 0.2345640165 |
| 2012 | 17.12 | 5 | 23 | urban_med | 2089800 | 0.0270610447 |
| 2012 | 17.12 | 5 | 24 | urban_high | 1003500 | 0.0129944293 |
| 2012 | 17.12 | 5 | 31 | barren | 138600 | 0.0017947463 |
| 2012 | 17.12 | 5 | 41 | dec_forest | 12822300 | 0.16603734 |
| 2012 | 17.12 | 5 | 42 | eg_forest | 311400 | 0.004032352 |
| 2012 | 17.12 | 5 | 43 | mix_forest | 31500 | 0.0004078969 |
| 2012 | 17.12 | 5 | 52 | scrub | 17100 | 0.0002214297 |
| 2012 | 17.12 | 5 | 71 | grass_herb | 941400 | 0.0121902897 |
| 2012 | 17.12 | 5 | 81 | pasture_hay | 6030000 | 0.0780831177 |
| 2012 | 17.12 | 5 | 82 | crop | 9876600 | 0.1278931543 |
| 2012 | 17.12 | 5 | 90 | woody_wetland | 446400 | 0.0057804816 |
| 2012 | 17.12 | 5 | 95 | em_herb_wetland | 45900 | 0.000594364 |
| 2012 | 20.12 | 5 | 11 | open_water | 1689300 | 0.0218606601 |
| 2012 | 20.12 | 5 | 21 | urban_open | 2646000 | 0.0342409914 |
| 2012 | 20.12 | 5 | 22 | urban_low | 845100 | 0.0109361534 |
| 2012 | 20.12 | 5 | 23 | urban_med | 91800 | 0.0011879528 |
| 2012 | 20.12 | 5 | 24 | urban_high | 45900 | 0.0005939764 |
| 2012 | 20.12 | 5 | 41 | dec_forest | 6025500 | 0.0779739582 |
| 2012 | 20.12 | 5 | 42 | eg_forest | 22500 | 0.0002911649 |
| 2012 | 20.12 | 5 | 43 | mix_forest | 81000 | 0.0010481936 |
| 2012 | 20.12 | 5 | 52 | scrub | 7200 | 9.32E-05 |
| 2012 | 20.12 | 5 | 81 | pasture_hay | 5013000 | 0.064871538 |
| 2012 | 20.12 | 5 | 82 | crop | 60516000 | 0.7831170949 |
| 2012 | 20.12 | 5 | 90 | woody_wetland | 154800 | 0.0020032145 |
| 2012 | 20.12 | 5 | 95 | em_herb_wetland | 137700 | 0.0017819291 |
| 2012 | 21.12 | 5 | 11 | open_water | 756900 | 0.0098046073 |
| 2012 | 21.12 | 5 | 21 | urban_open | 23922000 | 0.3098768886 |
| 2012 | 21.12 | 5 | 22 | urban_low | 14563800 | 0.188654169 |
| 2012 | 21.12 | 5 | 23 | urban_med | 7817400 | 0.1012637568 |
| 2012 | 21.12 | 5 | 24 | urban_high | 2520000 | 0.0326431636 |
| 2012 | 21.12 | 5 | 41 | dec_forest | 23015700 | 0.298137008 |
| 2012 | 21.12 | 5 | 42 | eg_forest | 1245600 | 0.0161350494 |
| 2012 | 21.12 | 5 | 43 | mix_forest | 18000 | 0.0002331655 |
| 2012 | 21.12 | 5 | 52 | scrub | 196200 | 0.0025415035 |
| 2012 | 21.12 | 5 | 71 | grass_herb | 641700 | 0.0083123484 |
| 2012 | 21.12 | 5 | 81 | pasture_hay | 1865700 | 0.0241675993 |
| 2012 | 21.12 | 5 | 82 | crop | 486900 | 0.0063071255 |
| 2012 | 21.12 | 5 | 90 | woody_wetland | 32400 | 0.0004196978 |
| 2012 | 21.12 | 5 | 95 | em_herb_wetland | 116100 | 0.0015039172 |
| 2012 | 26.12 | 5 | 11 | open_water | 1107000 | 0.0143251459 |
| 2012 | 26.12 | 5 | 21 | urban_open | 5816700 | 0.0752710714 |
| 2012 | 26.12 | 5 | 22 | urban_low | 1571400 | 0.0203347193 |
| 2012 | 26.12 | 5 | 23 | urban_med | 214200 | 0.0027718575 |
| 2012 | 26.12 | 5 | 24 | urban_high | 58500 | 0.0007570199 |
| 2012 | 26.12 | 5 | 31 | barren | 296100 | 0.0038316854 |
| 2012 | 26.12 | 5 | 41 | dec_forest | 39986100 | 0.5174405739 |
| 2012 | 26.12 | 5 | 42 | eg_forest | 526500 | 0.0068131791 |
| 2012 | 26.12 | 5 | 52 | scrub | 37800 | 0.0004891513 |
| 2012 | 26.12 | 5 | 71 | grass_herb | 415800 | 0.0053806645 |
| 2012 | 26.12 | 5 | 81 | pasture_hay | 24205500 | 0.3132315433 |
| 2012 | 26.12 | 5 | 82 | crop | 3007800 | 0.0389224695 |
| 2012 | 26.12 | 5 | 90 | woody_wetland | 29700 | 0.0003843332 |
| 2012 | 26.12 | 5 | 95 | em_herb_wetland | 3600 | 4.66E-05 |
| 2012 | 27.12 | 5 | 11 | open_water | 233100 | 0.00301805 |
| 2012 | 27.12 | 5 | 21 | urban_open | 5261400 | 0.0681217008 |
| 2012 | 27.12 | 5 | 22 | urban_low | 2355300 | 0.0304951233 |
| 2012 | 27.12 | 5 | 23 | urban_med | 279900 | 0.0036239906 |
| 2012 | 27.12 | 5 | 24 | urban_high | 63000 | 0.0008156892 |
| 2012 | 27.12 | 5 | 31 | barren | 119700 | 0.0015498095 |
| 2012 | 27.12 | 5 | 41 | dec_forest | 11530800 | 0.1492944288 |
| 2012 | 27.12 | 5 | 42 | eg_forest | 2007900 | 0.02599718 |
| 2012 | 27.12 | 5 | 43 | mix_forest | 11700 | 0.0001514851 |
| 2012 | 27.12 | 5 | 71 | grass_herb | 1783800 | 0.023095657 |
| 2012 | 27.12 | 5 | 81 | pasture_hay | 4377600 | 0.0566787466 |
| 2012 | 27.12 | 5 | 82 | crop | 48967200 | 0.6340002564 |
| 2012 | 27.12 | 5 | 90 | woody_wetland | 219600 | 0.0028432595 |
| 2012 | 27.12 | 5 | 95 | em_herb_wetland | 24300 | 0.000314623 |
| 2012 | 28.12 | 5 | 11 | open_water | 242100 | 0.0031356367 |
| 2012 | 28.12 | 5 | 21 | urban_open | 11792700 | 0.1527369795 |
| 2012 | 28.12 | 5 | 22 | urban_low | 7173000 | 0.0929034364 |
| 2012 | 28.12 | 5 | 23 | urban_med | 2430000 | 0.0314729333 |
| 2012 | 28.12 | 5 | 24 | urban_high | 1531800 | 0.0198396046 |
| 2012 | 28.12 | 5 | 31 | barren | 314100 | 0.004068168 |
| 2012 | 28.12 | 5 | 41 | dec_forest | 30369600 | 0.3933417261 |
| 2012 | 28.12 | 5 | 42 | eg_forest | 477000 | 0.0061780202 |
| 2012 | 28.12 | 5 | 43 | mix_forest | 5400 | 6.99E-05 |
| 2012 | 28.12 | 5 | 52 | scrub | 17100 | 0.0002214762 |
| 2012 | 28.12 | 5 | 71 | grass_herb | 2613600 | 0.0338508882 |
| 2012 | 28.12 | 5 | 81 | pasture_hay | 9941400 | 0.128759267 |
| 2012 | 28.12 | 5 | 82 | crop | 9441000 | 0.1222781741 |
| 2012 | 28.12 | 5 | 90 | woody_wetland | 827100 | 0.010712454 |
| 2012 | 28.12 | 5 | 95 | em_herb_wetland | 33300 | 0.0004312958 |
| 2012 | 30.12 | 5 | 11 | open_water | 209700 | 0.0027162192 |
| 2012 | 30.12 | 5 | 21 | urban_open | 18234900 | 0.2361944953 |
| 2012 | 30.12 | 5 | 22 | urban_low | 20949300 | 0.2713537963 |
| 2012 | 30.12 | 5 | 23 | urban_med | 7707600 | 0.0998356279 |
| 2012 | 30.12 | 5 | 24 | urban_high | 2465100 | 0.0319301477 |
| 2012 | 30.12 | 5 | 31 | barren | 55800 | 0.0007227708 |
| 2012 | 30.12 | 5 | 41 | dec_forest | 11683800 | 0.1513388746 |
| 2012 | 30.12 | 5 | 42 | eg_forest | 296100 | 0.0038353482 |
| 2012 | 30.12 | 5 | 52 | scrub | 297900 | 0.0038586633 |
| 2012 | 30.12 | 5 | 71 | grass_herb | 575100 | 0.007449202 |
| 2012 | 30.12 | 5 | 81 | pasture_hay | 3897000 | 0.0504773784 |
| 2012 | 30.12 | 5 | 82 | crop | 10546200 | 0.1366036768 |
| 2012 | 30.12 | 5 | 90 | woody_wetland | 259200 | 0.0033573868 |
| 2012 | 30.12 | 5 | 95 | em_herb_wetland | 25200 | 0.0003264126 |
| 2012 | 33.12 | 5 | 11 | open_water | 441900 | 0.0057216771 |
| 2012 | 33.12 | 5 | 21 | urban_open | 18553500 | 0.2402288671 |
| 2012 | 33.12 | 5 | 22 | urban_low | 14459400 | 0.1872188687 |
| 2012 | 33.12 | 5 | 23 | urban_med | 3309300 | 0.0428484863 |
| 2012 | 33.12 | 5 | 24 | urban_high | 968400 | 0.0125387466 |
| 2012 | 33.12 | 5 | 41 | dec_forest | 11025000 | 0.1427506001 |
| 2012 | 33.12 | 5 | 42 | eg_forest | 116100 | 0.0015032512 |
| 2012 | 33.12 | 5 | 52 | scrub | 321300 | 0.0041601603 |
| 2012 | 33.12 | 5 | 71 | grass_herb | 782100 | 0.0101265528 |
| 2012 | 33.12 | 5 | 81 | pasture_hay | 7045200 | 0.0912205468 |
| 2012 | 33.12 | 5 | 82 | crop | 19756800 | 0.2558090754 |
| 2012 | 33.12 | 5 | 90 | woody_wetland | 413100 | 0.0053487776 |
| 2012 | 33.12 | 5 | 95 | em_herb_wetland | 40500 | 0.00052439 |
| 2012 | 36.12 | 5 | 11 | open_water | 524700 | 0.0067963768 |
| 2012 | 36.12 | 5 | 21 | urban_open | 11805300 | 0.1529126497 |
| 2012 | 36.12 | 5 | 22 | urban_low | 8311500 | 0.107657873 |
| 2012 | 36.12 | 5 | 23 | urban_med | 1405800 | 0.0182091605 |
| 2012 | 36.12 | 5 | 24 | urban_high | 339300 | 0.0043949126 |
| 2012 | 36.12 | 5 | 31 | barren | 8100 | 0.0001049183 |
| 2012 | 36.12 | 5 | 41 | dec_forest | 9321300 | 0.1207376925 |
| 2012 | 36.12 | 5 | 42 | eg_forest | 147600 | 0.0019118453 |
| 2012 | 36.12 | 5 | 43 | mix_forest | 103500 | 0.0013406232 |
| 2012 | 36.12 | 5 | 71 | grass_herb | 199800 | 0.0025879857 |
| 2012 | 36.12 | 5 | 81 | pasture_hay | 17118000 | 0.2217274222 |
| 2012 | 36.12 | 5 | 82 | crop | 27846000 | 0.3606859328 |
| 2012 | 36.12 | 5 | 95 | em_herb_wetland | 72000 | 0.0009326075 |
| 2012 | 40.12 | 5 | 11 | open_water | 172800 | 0.0022371886 |
| 2012 | 40.12 | 5 | 21 | urban_open | 3839400 | 0.0497075342 |
| 2012 | 40.12 | 5 | 22 | urban_low | 65700 | 0.0008505977 |
| 2012 | 40.12 | 5 | 23 | urban_med | 8100 | 0.0001048682 |
| 2012 | 40.12 | 5 | 41 | dec_forest | 45661500 | 0.5911654354 |
| 2012 | 40.12 | 5 | 42 | eg_forest | 1952100 | 0.02527324 |
| 2012 | 40.12 | 5 | 43 | mix_forest | 4500 | 5.83E-05 |
| 2012 | 40.12 | 5 | 52 | scrub | 11700 | 0.0001514763 |
| 2012 | 40.12 | 5 | 71 | grass_herb | 630000 | 0.0081564168 |
| 2012 | 40.12 | 5 | 81 | pasture_hay | 11305800 | 0.1463727249 |
| 2012 | 40.12 | 5 | 82 | crop | 13486500 | 0.174605579 |
| 2012 | 40.12 | 5 | 90 | woody_wetland | 97200 | 0.0012584186 |
| 2012 | 40.12 | 5 | 95 | em_herb_wetland | 4500 | 5.83E-05 |
| 2012 | 41.12 | 5 | 11 | open_water | 393300 | 0.0050923498 |
| 2012 | 41.12 | 5 | 21 | urban_open | 11716200 | 0.151698421 |
| 2012 | 41.12 | 5 | 22 | urban_low | 28522800 | 0.3693060654 |
| 2012 | 41.12 | 5 | 23 | urban_med | 23844600 | 0.3087339043 |
| 2012 | 41.12 | 5 | 24 | urban_high | 10648800 | 0.1378779934 |
| 2012 | 41.12 | 5 | 41 | dec_forest | 1926000 | 0.0249373653 |
| 2012 | 41.12 | 5 | 71 | grass_herb | 138600 | 0.0017945581 |
| 2012 | 41.12 | 5 | 81 | pasture_hay | 43200 | 0.0005593428 |
| 2012 | 42.12 | 5 | 11 | open_water | 24300 | 0.0003147734 |
| 2012 | 42.12 | 5 | 21 | urban_open | 3787200 | 0.0490580116 |
| 2012 | 42.12 | 5 | 22 | urban_low | 47700 | 0.0006178885 |
| 2012 | 42.12 | 5 | 41 | dec_forest | 61214400 | 0.7929490767 |
| 2012 | 42.12 | 5 | 42 | eg_forest | 4266000 | 0.0552602126 |
| 2012 | 42.12 | 5 | 52 | scrub | 229500 | 0.0029728595 |
| 2012 | 42.12 | 5 | 71 | grass_herb | 108900 | 0.001410651 |
| 2012 | 42.12 | 5 | 81 | pasture_hay | 3607200 | 0.046726357 |
| 2012 | 42.12 | 5 | 82 | crop | 3906900 | 0.0506085618 |
| 2012 | 42.12 | 5 | 95 | em_herb_wetland | 6300 | 8.16E-05 |
| 2012 | 43.12 | 5 | 11 | open_water | 1678500 | 0.021723178 |
| 2012 | 43.12 | 5 | 21 | urban_open | 3111300 | 0.040266502 |
| 2012 | 43.12 | 5 | 22 | urban_low | 1562400 | 0.0202206096 |
| 2012 | 43.12 | 5 | 23 | urban_med | 248400 | 0.0032147974 |
| 2012 | 43.12 | 5 | 24 | urban_high | 57600 | 0.0007454603 |
| 2012 | 43.12 | 5 | 31 | barren | 27000 | 0.0003494345 |
| 2012 | 43.12 | 5 | 41 | dec_forest | 9309600 | 0.1204850151 |
| 2012 | 43.12 | 5 | 42 | eg_forest | 59400 | 0.0007687559 |
| 2012 | 43.12 | 5 | 43 | mix_forest | 65700 | 0.0008502906 |
| 2012 | 43.12 | 5 | 52 | scrub | 18900 | 0.0002446041 |
| 2012 | 43.12 | 5 | 71 | grass_herb | 52200 | 0.0006755734 |
| 2012 | 43.12 | 5 | 81 | pasture_hay | 7713900 | 0.0998334362 |
| 2012 | 43.12 | 5 | 82 | crop | 53209800 | 0.688642214 |
| 2012 | 43.12 | 5 | 90 | woody_wetland | 125100 | 0.0016190465 |
| 2012 | 43.12 | 5 | 95 | em_herb_wetland | 27900 | 0.0003610823 |
| 2012 | 44.12 | 5 | 11 | open_water | 838800 | 0.0108623443 |
| 2012 | 44.12 | 5 | 21 | urban_open | 2831400 | 0.0366662393 |
| 2012 | 44.12 | 5 | 22 | urban_low | 914400 | 0.0118413538 |
| 2012 | 44.12 | 5 | 23 | urban_med | 4500 | 5.83E-05 |
| 2012 | 44.12 | 5 | 41 | dec_forest | 42300000 | 0.5477791634 |
| 2012 | 44.12 | 5 | 42 | eg_forest | 383400 | 0.0049649771 |
| 2012 | 44.12 | 5 | 43 | mix_forest | 8100 | 0.0001048939 |
| 2012 | 44.12 | 5 | 52 | scrub | 2258100 | 0.0292420834 |
| 2012 | 44.12 | 5 | 71 | grass_herb | 2420100 | 0.0313399611 |
| 2012 | 44.12 | 5 | 81 | pasture_hay | 4354200 | 0.0563862892 |
| 2012 | 44.12 | 5 | 82 | crop | 15956100 | 0.2066292934 |
| 2012 | 44.12 | 5 | 90 | woody_wetland | 4869900 | 0.063064533 |
| 2012 | 44.12 | 5 | 95 | em_herb_wetland | 81900 | 0.0010605937 |
| 2012 | 45.12 | 5 | 11 | open_water | 65700 | 0.0008502906 |
| 2012 | 45.12 | 5 | 21 | urban_open | 4759200 | 0.0615936543 |
| 2012 | 45.12 | 5 | 22 | urban_low | 5656500 | 0.0732065274 |
| 2012 | 45.12 | 5 | 23 | urban_med | 2108700 | 0.0272908343 |
| 2012 | 45.12 | 5 | 24 | urban_high | 652500 | 0.008444667 |
| 2012 | 45.12 | 5 | 31 | barren | 9900 | 0.000128126 |
| 2012 | 45.12 | 5 | 41 | dec_forest | 1700100 | 0.0220027256 |
| 2012 | 45.12 | 5 | 71 | grass_herb | 683100 | 0.0088406928 |
| 2012 | 45.12 | 5 | 81 | pasture_hay | 6059700 | 0.0784247493 |
| 2012 | 45.12 | 5 | 82 | crop | 55518300 | 0.7185188636 |
| 2012 | 45.12 | 5 | 90 | woody_wetland | 900 | 1.16E-05 |
| 2012 | 45.12 | 5 | 95 | em_herb_wetland | 53100 | 0.0006872212 |
| 2012 | 47.12 | 5 | 11 | open_water | 224100 | 0.002900205 |
| 2012 | 47.12 | 5 | 21 | urban_open | 14506200 | 0.1877329482 |
| 2012 | 47.12 | 5 | 22 | urban_low | 23015700 | 0.2978592061 |
| 2012 | 47.12 | 5 | 23 | urban_med | 13993200 | 0.1810939247 |
| 2012 | 47.12 | 5 | 24 | urban_high | 8493300 | 0.1099166045 |
| 2012 | 47.12 | 5 | 41 | dec_forest | 2022300 | 0.0261717294 |
| 2012 | 47.12 | 5 | 42 | eg_forest | 17100 | 0.0002213008 |
| 2012 | 47.12 | 5 | 43 | mix_forest | 7200 | 9.32E-05 |
| 2012 | 47.12 | 5 | 71 | grass_herb | 377100 | 0.0048802646 |
| 2012 | 47.12 | 5 | 81 | pasture_hay | 2027700 | 0.0262416139 |
| 2012 | 47.12 | 5 | 82 | crop | 12582000 | 0.1628307864 |
| 2012 | 47.12 | 5 | 95 | em_herb_wetland | 4500 | 0.000058237 |
| 2012 | 50.12 | 5 | 11 | open_water | 5861700 | 0.0759064368 |
| 2012 | 50.12 | 5 | 21 | urban_open | 6723000 | 0.087059893 |
| 2012 | 50.12 | 5 | 22 | urban_low | 1515600 | 0.0196263534 |
| 2012 | 50.12 | 5 | 23 | urban_med | 968400 | 0.0125403541 |
| 2012 | 50.12 | 5 | 24 | urban_high | 529200 | 0.0068529072 |
| 2012 | 50.12 | 5 | 31 | barren | 1329300 | 0.0172138503 |
| 2012 | 50.12 | 5 | 41 | dec_forest | 50363100 | 0.6521799937 |
| 2012 | 50.12 | 5 | 42 | eg_forest | 267300 | 0.0034614174 |
| 2012 | 50.12 | 5 | 43 | mix_forest | 5400 | 6.99E-05 |
| 2012 | 50.12 | 5 | 52 | scrub | 10800 | 0.0001398552 |
| 2012 | 50.12 | 5 | 71 | grass_herb | 1568700 | 0.020313975 |
| 2012 | 50.12 | 5 | 81 | pasture_hay | 7062300 | 0.0914536788 |
| 2012 | 50.12 | 5 | 82 | crop | 981900 | 0.0127151731 |
| 2012 | 50.12 | 5 | 90 | woody_wetland | 27000 | 0.0003496381 |
| 2012 | 50.12 | 5 | 95 | em_herb_wetland | 9000 | 0.000116546 |
| 2012 | 52.12 | 5 | 11 | open_water | 81900 | 0.0010603095 |
| 2012 | 52.12 | 5 | 21 | urban_open | 6383700 | 0.0826458799 |
| 2012 | 52.12 | 5 | 22 | urban_low | 815400 | 0.0105564877 |
| 2012 | 52.12 | 5 | 23 | urban_med | 103500 | 0.0013399515 |
| 2012 | 52.12 | 5 | 24 | urban_high | 15300 | 0.0001980798 |
| 2012 | 52.12 | 5 | 31 | barren | 27900 | 0.0003612043 |
| 2012 | 52.12 | 5 | 41 | dec_forest | 43625700 | 0.5647953952 |
| 2012 | 52.12 | 5 | 42 | eg_forest | 792900 | 0.0102651939 |
| 2012 | 52.12 | 5 | 52 | scrub | 97200 | 0.0012583893 |
| 2012 | 52.12 | 5 | 71 | grass_herb | 424800 | 0.0054996271 |
| 2012 | 52.12 | 5 | 81 | pasture_hay | 11982600 | 0.1551314318 |
| 2012 | 52.12 | 5 | 82 | crop | 12744000 | 0.1649888143 |
| 2012 | 52.12 | 5 | 90 | woody_wetland | 136800 | 0.0017710664 |
| 2012 | 52.12 | 5 | 95 | em_herb_wetland | 9900 | 0.0001281693 |
| 2012 | 53.12 | 5 | 11 | open_water | 1333800 | 0.0172687019 |
| 2012 | 53.12 | 5 | 21 | urban_open | 8764200 | 0.1134700536 |
| 2012 | 53.12 | 5 | 22 | urban_low | 6154200 | 0.0796783966 |
| 2012 | 53.12 | 5 | 23 | urban_med | 1434600 | 0.018573759 |
| 2012 | 53.12 | 5 | 24 | urban_high | 216900 | 0.0028082032 |
| 2012 | 53.12 | 5 | 41 | dec_forest | 15074100 | 0.1951642974 |
| 2012 | 53.12 | 5 | 42 | eg_forest | 577800 | 0.0074807737 |
| 2012 | 53.12 | 5 | 43 | mix_forest | 70200 | 0.000908879 |
| 2012 | 53.12 | 5 | 52 | scrub | 388800 | 0.0050337917 |
| 2012 | 53.12 | 5 | 71 | grass_herb | 1350900 | 0.0174900955 |
| 2012 | 53.12 | 5 | 81 | pasture_hay | 28381500 | 0.3674551387 |
| 2012 | 53.12 | 5 | 82 | crop | 13449600 | 0.174131904 |
| 2012 | 53.12 | 5 | 90 | woody_wetland | 8100 | 0.0001048707 |
| 2012 | 53.12 | 5 | 95 | em_herb_wetland | 33300 | 0.0004311349 |
| 2012 | 54.12 | 5 | 11 | open_water | 154800 | 0.0020051528 |
| 2012 | 54.12 | 5 | 21 | urban_open | 7944300 | 0.1029039742 |
| 2012 | 54.12 | 5 | 22 | urban_low | 7457400 | 0.0965970692 |
| 2012 | 54.12 | 5 | 23 | urban_med | 1938600 | 0.0251110412 |
| 2012 | 54.12 | 5 | 24 | urban_high | 379800 | 0.004919619 |
| 2012 | 54.12 | 5 | 41 | dec_forest | 9362700 | 0.1212767694 |
| 2012 | 54.12 | 5 | 42 | eg_forest | 56700 | 0.0007344455 |
| 2012 | 54.12 | 5 | 43 | mix_forest | 78300 | 0.0010142343 |
| 2012 | 54.12 | 5 | 71 | grass_herb | 1386000 | 0.0179531121 |
| 2012 | 54.12 | 5 | 81 | pasture_hay | 8185500 | 0.106028282 |
| 2012 | 54.12 | 5 | 82 | crop | 40211100 | 0.5208617494 |
| 2012 | 54.12 | 5 | 95 | em_herb_wetland | 45900 | 0.0005945511 |
| 2012 | 101.12 | 5 | 11 | open_water | 914400 | 0.0118379046 |
| 2012 | 101.12 | 5 | 21 | urban_open | 11457000 | 0.1483233519 |
| 2012 | 101.12 | 5 | 22 | urban_low | 8798400 | 0.1139048773 |
| 2012 | 101.12 | 5 | 23 | urban_med | 3961800 | 0.0512898189 |
| 2012 | 101.12 | 5 | 24 | urban_high | 2174400 | 0.0281499779 |
| 2012 | 101.12 | 5 | 31 | barren | 80100 | 0.0010369818 |
| 2012 | 101.12 | 5 | 41 | dec_forest | 12272400 | 0.1588795936 |
| 2012 | 101.12 | 5 | 42 | eg_forest | 378900 | 0.0049052735 |
| 2012 | 101.12 | 5 | 43 | mix_forest | 11700 | 0.0001514693 |
| 2012 | 101.12 | 5 | 52 | scrub | 9000 | 0.0001165148 |
| 2012 | 101.12 | 5 | 71 | grass_herb | 691200 | 0.0089483373 |
| 2012 | 101.12 | 5 | 81 | pasture_hay | 7560000 | 0.0978724396 |
| 2012 | 101.12 | 5 | 82 | crop | 28079100 | 0.3635145527 |
| 2012 | 101.12 | 5 | 90 | woody_wetland | 820800 | 0.0106261506 |
| 2012 | 101.12 | 5 | 95 | em_herb_wetland | 34200 | 0.0004427563 |
| 2012 | 102.12 | 5 | 11 | open_water | 1336500 | 0.0172966047 |
| 2012 | 102.12 | 5 | 21 | urban_open | 25892100 | 0.3350882302 |
| 2012 | 102.12 | 5 | 22 | urban_low | 29086200 | 0.3764253684 |
| 2012 | 102.12 | 5 | 23 | urban_med | 6066900 | 0.0785161027 |
| 2012 | 102.12 | 5 | 24 | urban_high | 1872000 | 0.0242268942 |
| 2012 | 102.12 | 5 | 31 | barren | 106200 | 0.0013744103 |
| 2012 | 102.12 | 5 | 41 | dec_forest | 8234100 | 0.1065633918 |
| 2012 | 102.12 | 5 | 42 | eg_forest | 151200 | 0.0019567876 |
| 2012 | 102.12 | 5 | 52 | scrub | 79200 | 0.001024984 |
| 2012 | 102.12 | 5 | 71 | grass_herb | 1071000 | 0.0138605789 |
| 2012 | 102.12 | 5 | 81 | pasture_hay | 2202300 | 0.0285015433 |
| 2012 | 102.12 | 5 | 82 | crop | 351900 | 0.0045541902 |
| 2012 | 102.12 | 5 | 90 | woody_wetland | 813600 | 0.0105293809 |
| 2012 | 102.12 | 5 | 95 | em_herb_wetland | 6300 | 8.15E-05 |
| 2012 | 104.12 | 5 | 11 | open_water | 10800 | 0.0001398194 |
| 2012 | 104.12 | 5 | 21 | urban_open | 2202300 | 0.028511506 |
| 2012 | 104.12 | 5 | 22 | urban_low | 694800 | 0.0089950481 |
| 2012 | 104.12 | 5 | 31 | barren | 7200 | 9.32E-05 |
| 2012 | 104.12 | 5 | 41 | dec_forest | 973800 | 0.0126070492 |
| 2012 | 104.12 | 5 | 71 | grass_herb | 478800 | 0.0061986601 |
| 2012 | 104.12 | 5 | 81 | pasture_hay | 1676700 | 0.0217069618 |
| 2012 | 104.12 | 5 | 82 | crop | 71198100 | 0.9217477425 |
| 2012 | 107.12 | 5 | 11 | open_water | 48600 | 0.000629246 |
| 2012 | 107.12 | 5 | 21 | urban_open | 3574800 | 0.0462845357 |
| 2012 | 107.12 | 5 | 22 | urban_low | 285300 | 0.0036939068 |
| 2012 | 107.12 | 5 | 23 | urban_med | 15300 | 0.0001980959 |
| 2012 | 107.12 | 5 | 41 | dec_forest | 9424800 | 0.1220271042 |
| 2012 | 107.12 | 5 | 43 | mix_forest | 1944000 | 0.0251698381 |
| 2012 | 107.12 | 5 | 81 | pasture_hay | 2641500 | 0.0342006828 |
| 2012 | 107.12 | 5 | 82 | crop | 59291100 | 0.7676684107 |
| 2012 | 107.12 | 5 | 95 | em_herb_wetland | 9900 | 0.0001281797 |
| 2012 | 108.12 | 5 | 11 | open_water | 658800 | 0.0085337561 |
| 2012 | 108.12 | 5 | 21 | urban_open | 12379500 | 0.1603576716 |
| 2012 | 108.12 | 5 | 22 | urban_low | 31243500 | 0.4047122189 |
| 2012 | 108.12 | 5 | 23 | urban_med | 21644100 | 0.2803665318 |
| 2012 | 108.12 | 5 | 24 | urban_high | 7744500 | 0.1003182671 |
| 2012 | 108.12 | 5 | 41 | dec_forest | 3353400 | 0.0434382177 |
| 2012 | 108.12 | 5 | 71 | grass_herb | 128700 | 0.0016671136 |
| 2012 | 108.12 | 5 | 81 | pasture_hay | 46800 | 0.0006062231 |
| 2012 | 111.12 | 5 | 11 | open_water | 770400 | 0.0099700665 |
| 2012 | 111.12 | 5 | 21 | urban_open | 22170600 | 0.2869189466 |
| 2012 | 111.12 | 5 | 22 | urban_low | 27757800 | 0.3592252233 |
| 2012 | 111.12 | 5 | 23 | urban_med | 10647000 | 0.1377872509 |
| 2012 | 111.12 | 5 | 24 | urban_high | 6072300 | 0.0785841574 |
| 2012 | 111.12 | 5 | 31 | barren | 123300 | 0.0015956765 |
| 2012 | 111.12 | 5 | 41 | dec_forest | 4109400 | 0.0531814529 |
| 2012 | 111.12 | 5 | 42 | eg_forest | 177300 | 0.002294513 |
| 2012 | 111.12 | 5 | 71 | grass_herb | 906300 | 0.0117288049 |
| 2012 | 111.12 | 5 | 81 | pasture_hay | 2277000 | 0.0294676031 |
| 2012 | 111.12 | 5 | 82 | crop | 2028600 | 0.0262529555 |
| 2012 | 111.12 | 5 | 90 | woody_wetland | 222300 | 0.0028768767 |
| 2012 | 111.12 | 5 | 95 | em_herb_wetland | 9000 | 0.0001164727 |
| 2012 | 112.12 | 5 | 11 | open_water | 1441800 | 0.0186593675 |
| 2012 | 112.12 | 5 | 21 | urban_open | 24381900 | 0.3155436492 |
| 2012 | 112.12 | 5 | 22 | urban_low | 17623800 | 0.2280822317 |
| 2012 | 112.12 | 5 | 23 | urban_med | 5037300 | 0.0651913109 |
| 2012 | 112.12 | 5 | 24 | urban_high | 3972600 | 0.0514122649 |
| 2012 | 112.12 | 5 | 31 | barren | 65700 | 0.0008502708 |
| 2012 | 112.12 | 5 | 41 | dec_forest | 11325600 | 0.1465727098 |
| 2012 | 112.12 | 5 | 42 | eg_forest | 405900 | 0.0052530429 |
| 2012 | 112.12 | 5 | 43 | mix_forest | 39600 | 0.000512492 |
| 2012 | 112.12 | 5 | 52 | scrub | 18900 | 0.0002445985 |
| 2012 | 112.12 | 5 | 71 | grass_herb | 1102500 | 0.014268243 |
| 2012 | 112.12 | 5 | 81 | pasture_hay | 4689900 | 0.0606953585 |
| 2012 | 112.12 | 5 | 82 | crop | 6640200 | 0.0859355891 |
| 2012 | 112.12 | 5 | 90 | woody_wetland | 484200 | 0.0062663794 |
| 2012 | 112.12 | 5 | 95 | em_herb_wetland | 39600 | 0.000512492 |
| 2012 | 113.12 | 5 | 11 | open_water | 574200 | 0.007434511 |
| 2012 | 113.12 | 5 | 21 | urban_open | 9429300 | 0.1220867903 |
| 2012 | 113.12 | 5 | 22 | urban_low | 35446500 | 0.4589470495 |
| 2012 | 113.12 | 5 | 23 | urban_med | 20666700 | 0.2675841335 |
| 2012 | 113.12 | 5 | 24 | urban_high | 5544000 | 0.071781486 |
| 2012 | 113.12 | 5 | 31 | barren | 4500 | 5.83E-05 |
| 2012 | 113.12 | 5 | 41 | dec_forest | 4829400 | 0.0625291321 |
| 2012 | 113.12 | 5 | 42 | eg_forest | 73800 | 0.0009555328 |
| 2012 | 113.12 | 5 | 43 | mix_forest | 4500 | 5.83E-05 |
| 2012 | 113.12 | 5 | 71 | grass_herb | 183600 | 0.0023771791 |
| 2012 | 113.12 | 5 | 90 | woody_wetland | 450000 | 0.0058264193 |
| 2012 | 113.12 | 5 | 95 | em_herb_wetland | 27900 | 0.000361238 |
| 2012 | 115.12 | 5 | 11 | open_water | 428400 | 0.0055443607 |
| 2012 | 115.12 | 5 | 21 | urban_open | 22279500 | 0.2883417003 |
| 2012 | 115.12 | 5 | 22 | urban_low | 19484100 | 0.2521635819 |
| 2012 | 115.12 | 5 | 23 | urban_med | 9469800 | 0.1225583264 |
| 2012 | 115.12 | 5 | 24 | urban_high | 6163200 | 0.0797642482 |
| 2012 | 115.12 | 5 | 41 | dec_forest | 13931100 | 0.1802965534 |
| 2012 | 115.12 | 5 | 42 | eg_forest | 252000 | 0.0032613887 |
| 2012 | 115.12 | 5 | 52 | scrub | 192600 | 0.0024926328 |
| 2012 | 115.12 | 5 | 71 | grass_herb | 644400 | 0.0083398367 |
| 2012 | 115.12 | 5 | 81 | pasture_hay | 3556800 | 0.0460321713 |
| 2012 | 115.12 | 5 | 82 | crop | 774900 | 0.0100287701 |
| 2012 | 115.12 | 5 | 90 | woody_wetland | 86400 | 0.0011181904 |
| 2012 | 115.12 | 5 | 95 | em_herb_wetland | 4500 | 5.82E-05 |
| 2013 | 3.13 | 0.5 | 21 | urban_open | 179100 | 0.2360616845 |
| 2013 | 3.13 | 0.5 | 22 | urban_low | 475200 | 0.6263345196 |
| 2013 | 3.13 | 0.5 | 23 | urban_med | 78300 | 0.103202847 |
| 2013 | 3.13 | 0.5 | 24 | urban_high | 26100 | 0.034400949 |
| 2013 | 7.13 | 0.5 | 21 | urban_open | 41400 | 0.0545670225 |
| 2013 | 7.13 | 0.5 | 22 | urban_low | 1800 | 0.0023724792 |
| 2013 | 7.13 | 0.5 | 41 | dec_forest | 675000 | 0.8896797153 |
| 2013 | 7.13 | 0.5 | 42 | eg_forest | 35100 | 0.0462633452 |
| 2013 | 7.13 | 0.5 | 52 | scrub | 5400 | 0.0071174377 |
| 2013 | 11.13 | 0.5 | 11 | open_water | 5400 | 0.0070754717 |
| 2013 | 11.13 | 0.5 | 21 | urban_open | 25200 | 0.0330188679 |
| 2013 | 11.13 | 0.5 | 22 | urban_low | 330300 | 0.4327830189 |
| 2013 | 11.13 | 0.5 | 23 | urban_med | 10800 | 0.0141509434 |
| 2013 | 11.13 | 0.5 | 41 | dec_forest | 127800 | 0.1674528302 |
| 2013 | 11.13 | 0.5 | 82 | crop | 263700 | 0.3455188679 |
| 2013 | 14.13 | 0.5 | 11 | open_water | 83700 | 0.1108462455 |
| 2013 | 14.13 | 0.5 | 21 | urban_open | 153000 | 0.2026221692 |
| 2013 | 14.13 | 0.5 | 22 | urban_low | 25200 | 0.0333730632 |
| 2013 | 14.13 | 0.5 | 41 | dec_forest | 347400 | 0.4600715137 |
| 2013 | 14.13 | 0.5 | 71 | grass_herb | 15300 | 0.0202622169 |
| 2013 | 14.13 | 0.5 | 81 | pasture_hay | 16200 | 0.021454112 |
| 2013 | 14.13 | 0.5 | 82 | crop | 111600 | 0.147794994 |
| 2013 | 14.13 | 0.5 | 90 | woody_wetland | 2700 | 0.0035756853 |
| 2013 | 16.13 | 0.5 | 21 | urban_open | 9000 | 0.0118906064 |
| 2013 | 16.13 | 0.5 | 22 | urban_low | 11700 | 0.0154577883 |
| 2013 | 16.13 | 0.5 | 41 | dec_forest | 597600 | 0.7895362663 |
| 2013 | 16.13 | 0.5 | 42 | eg_forest | 9000 | 0.0118906064 |
| 2013 | 16.13 | 0.5 | 71 | grass_herb | 9900 | 0.0130796671 |
| 2013 | 16.13 | 0.5 | 81 | pasture_hay | 68400 | 0.0903686088 |
| 2013 | 16.13 | 0.5 | 82 | crop | 25200 | 0.033293698 |
| 2013 | 16.13 | 0.5 | 90 | woody_wetland | 26100 | 0.0344827586 |
| 2013 | 22.13 | 0.5 | 21 | urban_open | 197100 | 0.2588652482 |
| 2013 | 22.13 | 0.5 | 22 | urban_low | 486000 | 0.6382978723 |
| 2013 | 22.13 | 0.5 | 23 | urban_med | 28800 | 0.0378250591 |
| 2013 | 22.13 | 0.5 | 24 | urban_high | 31500 | 0.0413711584 |
| 2013 | 22.13 | 0.5 | 41 | dec_forest | 18000 | 0.0236406619 |
| 2013 | 23.13 | 0.5 | 21 | urban_open | 36000 | 0.0472813239 |
| 2013 | 23.13 | 0.5 | 22 | urban_low | 3600 | 0.0047281324 |
| 2013 | 23.13 | 0.5 | 41 | dec_forest | 393300 | 0.5165484634 |
| 2013 | 23.13 | 0.5 | 42 | eg_forest | 22500 | 0.0295508274 |
| 2013 | 23.13 | 0.5 | 71 | grass_herb | 10800 | 0.0141843972 |
| 2013 | 23.13 | 0.5 | 81 | pasture_hay | 187200 | 0.2458628842 |
| 2013 | 23.13 | 0.5 | 82 | crop | 108000 | 0.1418439716 |
| 2013 | 26.13 | 0.5 | 21 | urban_open | 61200 | 0.0805687204 |
| 2013 | 26.13 | 0.5 | 41 | dec_forest | 53100 | 0.0699052133 |
| 2013 | 26.13 | 0.5 | 82 | crop | 645300 | 0.8495260664 |
| 2013 | 28.13 | 0.5 | 21 | urban_open | 180900 | 0.2384341637 |
| 2013 | 28.13 | 0.5 | 22 | urban_low | 126000 | 0.1660735469 |
| 2013 | 28.13 | 0.5 | 41 | dec_forest | 330300 | 0.4353499407 |
| 2013 | 28.13 | 0.5 | 71 | grass_herb | 18900 | 0.024911032 |
| 2013 | 28.13 | 0.5 | 81 | pasture_hay | 102600 | 0.1352313167 |
| 2013 | 36.13 | 0.5 | 21 | urban_open | 257400 | 0.3392645314 |
| 2013 | 36.13 | 0.5 | 22 | urban_low | 221400 | 0.2918149466 |
| 2013 | 36.13 | 0.5 | 23 | urban_med | 43200 | 0.0569395018 |
| 2013 | 36.13 | 0.5 | 41 | dec_forest | 233100 | 0.3072360617 |
| 2013 | 36.13 | 0.5 | 71 | grass_herb | 3600 | 0.0047449585 |
| 2013 | 37.13 | 0.5 | 11 | open_water | 10800 | 0.0141176471 |
| 2013 | 37.13 | 0.5 | 21 | urban_open | 286200 | 0.3741176471 |
| 2013 | 37.13 | 0.5 | 22 | urban_low | 273600 | 0.3576470588 |
| 2013 | 37.13 | 0.5 | 23 | urban_med | 25200 | 0.0329411765 |
| 2013 | 37.13 | 0.5 | 24 | urban_high | 13500 | 0.0176470588 |
| 2013 | 37.13 | 0.5 | 41 | dec_forest | 15300 | 0.02 |
| 2013 | 37.13 | 0.5 | 81 | pasture_hay | 51300 | 0.0670588235 |
| 2013 | 37.13 | 0.5 | 82 | crop | 89100 | 0.1164705882 |
| 2013 | 38.13 | 0.5 | 11 | open_water | 1800 | 0.0023255814 |
| 2013 | 38.13 | 0.5 | 21 | urban_open | 48600 | 0.0627906977 |
| 2013 | 38.13 | 0.5 | 41 | dec_forest | 355500 | 0.4593023256 |
| 2013 | 38.13 | 0.5 | 42 | eg_forest | 4500 | 0.0058139535 |
| 2013 | 38.13 | 0.5 | 81 | pasture_hay | 202500 | 0.261627907 |
| 2013 | 38.13 | 0.5 | 82 | crop | 161100 | 0.2081395349 |
| 2013 | 39.13 | 0.5 | 21 | urban_open | 12600 | 0.0165680473 |
| 2013 | 39.13 | 0.5 | 22 | urban_low | 4500 | 0.0059171598 |
| 2013 | 39.13 | 0.5 | 41 | dec_forest | 89100 | 0.1171597633 |
| 2013 | 39.13 | 0.5 | 52 | scrub | 24300 | 0.0319526627 |
| 2013 | 39.13 | 0.5 | 81 | pasture_hay | 100800 | 0.1325443787 |
| 2013 | 39.13 | 0.5 | 82 | crop | 522000 | 0.6863905325 |
| 2013 | 39.13 | 0.5 | 90 | woody_wetland | 7200 | 0.0094674556 |
| 2013 | 40.13 | 0.5 | 21 | urban_open | 260100 | 0.341607565 |
| 2013 | 40.13 | 0.5 | 22 | urban_low | 301500 | 0.3959810875 |
| 2013 | 40.13 | 0.5 | 23 | urban_med | 33300 | 0.0437352246 |
| 2013 | 40.13 | 0.5 | 24 | urban_high | 7200 | 0.0094562648 |
| 2013 | 40.13 | 0.5 | 41 | dec_forest | 21600 | 0.0283687943 |
| 2013 | 40.13 | 0.5 | 81 | pasture_hay | 10800 | 0.0141843972 |
| 2013 | 40.13 | 0.5 | 82 | crop | 126900 | 0.1666666667 |
| 2013 | 42.13 | 0.5 | 21 | urban_open | 27900 | 0.0365566038 |
| 2013 | 42.13 | 0.5 | 41 | dec_forest | 579600 | 0.7594339623 |
| 2013 | 42.13 | 0.5 | 42 | eg_forest | 13500 | 0.0176886792 |
| 2013 | 42.13 | 0.5 | 52 | scrub | 2700 | 0.0035377358 |
| 2013 | 42.13 | 0.5 | 81 | pasture_hay | 139500 | 0.1827830189 |
| 2013 | 43.13 | 0.5 | 11 | open_water | 8100 | 0.0106382979 |
| 2013 | 43.13 | 0.5 | 21 | urban_open | 94500 | 0.1241134752 |
| 2013 | 43.13 | 0.5 | 22 | urban_low | 21600 | 0.0283687943 |
| 2013 | 43.13 | 0.5 | 23 | urban_med | 4500 | 0.0059101655 |
| 2013 | 43.13 | 0.5 | 41 | dec_forest | 546300 | 0.7174940898 |
| 2013 | 43.13 | 0.5 | 71 | grass_herb | 4500 | 0.0059101655 |
| 2013 | 43.13 | 0.5 | 81 | pasture_hay | 72900 | 0.0957446809 |
| 2013 | 43.13 | 0.5 | 82 | crop | 9000 | 0.011820331 |
| 2013 | 46.13 | 0.5 | 11 | open_water | 179100 | 0.2322053676 |
| 2013 | 46.13 | 0.5 | 21 | urban_open | 255600 | 0.3313885648 |
| 2013 | 46.13 | 0.5 | 22 | urban_low | 61200 | 0.0793465578 |
| 2013 | 46.13 | 0.5 | 41 | dec_forest | 213300 | 0.276546091 |
| 2013 | 46.13 | 0.5 | 81 | pasture_hay | 48600 | 0.0630105018 |
| 2013 | 46.13 | 0.5 | 82 | crop | 8100 | 0.0105017503 |
| 2013 | 46.13 | 0.5 | 90 | woody_wetland | 5400 | 0.0070011669 |
| 2013 | 47.13 | 0.5 | 21 | urban_open | 84600 | 0.1105882353 |
| 2013 | 47.13 | 0.5 | 22 | urban_low | 9000 | 0.0117647059 |
| 2013 | 47.13 | 0.5 | 41 | dec_forest | 284400 | 0.3717647059 |
| 2013 | 47.13 | 0.5 | 71 | grass_herb | 14400 | 0.0188235294 |
| 2013 | 47.13 | 0.5 | 81 | pasture_hay | 71100 | 0.0929411765 |
| 2013 | 47.13 | 0.5 | 82 | crop | 301500 | 0.3941176471 |
| 2013 | 3.13 | 1 | 21 | urban_open | 519300 | 0.16831972 |
| 2013 | 3.13 | 1 | 22 | urban_low | 1737900 | 0.563302217 |
| 2013 | 3.13 | 1 | 23 | urban_med | 479700 | 0.1554842474 |
| 2013 | 3.13 | 1 | 24 | urban_high | 236700 | 0.0767211202 |
| 2013 | 3.13 | 1 | 41 | dec_forest | 89100 | 0.0288798133 |
| 2013 | 3.13 | 1 | 71 | grass_herb | 5400 | 0.0017502917 |
| 2013 | 3.13 | 1 | 90 | woody_wetland | 17100 | 0.0055425904 |
| 2013 | 7.13 | 1 | 11 | open_water | 53100 | 0.0172413793 |
| 2013 | 7.13 | 1 | 21 | urban_open | 240300 | 0.078024547 |
| 2013 | 7.13 | 1 | 22 | urban_low | 33300 | 0.0108123904 |
| 2013 | 7.13 | 1 | 41 | dec_forest | 2129400 | 0.691408533 |
| 2013 | 7.13 | 1 | 42 | eg_forest | 190800 | 0.0619520748 |
| 2013 | 7.13 | 1 | 52 | scrub | 21600 | 0.0070134424 |
| 2013 | 7.13 | 1 | 71 | grass_herb | 32400 | 0.0105201636 |
| 2013 | 7.13 | 1 | 81 | pasture_hay | 275400 | 0.089421391 |
| 2013 | 7.13 | 1 | 82 | crop | 103500 | 0.0336060783 |
| 2013 | 11.13 | 1 | 11 | open_water | 18900 | 0.0061188811 |
| 2013 | 11.13 | 1 | 21 | urban_open | 166500 | 0.0539044289 |
| 2013 | 11.13 | 1 | 22 | urban_low | 980100 | 0.3173076923 |
| 2013 | 11.13 | 1 | 23 | urban_med | 76500 | 0.0247668998 |
| 2013 | 11.13 | 1 | 41 | dec_forest | 382500 | 0.1238344988 |
| 2013 | 11.13 | 1 | 71 | grass_herb | 9000 | 0.0029137529 |
| 2013 | 11.13 | 1 | 81 | pasture_hay | 285300 | 0.0923659674 |
| 2013 | 11.13 | 1 | 82 | crop | 1170000 | 0.3787878788 |
| 2013 | 14.13 | 1 | 11 | open_water | 207900 | 0.0675043834 |
| 2013 | 14.13 | 1 | 21 | urban_open | 349200 | 0.113383986 |
| 2013 | 14.13 | 1 | 22 | urban_low | 78300 | 0.0254237288 |
| 2013 | 14.13 | 1 | 41 | dec_forest | 1689300 | 0.5485096435 |
| 2013 | 14.13 | 1 | 71 | grass_herb | 45900 | 0.0149035652 |
| 2013 | 14.13 | 1 | 81 | pasture_hay | 430200 | 0.1396843951 |
| 2013 | 14.13 | 1 | 82 | crop | 272700 | 0.0885447107 |
| 2013 | 14.13 | 1 | 90 | woody_wetland | 6300 | 0.0020455874 |
| 2013 | 16.13 | 1 | 11 | open_water | 23400 | 0.0075912409 |
| 2013 | 16.13 | 1 | 21 | urban_open | 252900 | 0.0820437956 |
| 2013 | 16.13 | 1 | 22 | urban_low | 82800 | 0.0268613139 |
| 2013 | 16.13 | 1 | 23 | urban_med | 23400 | 0.0075912409 |
| 2013 | 16.13 | 1 | 41 | dec_forest | 1863000 | 0.604379562 |
| 2013 | 16.13 | 1 | 42 | eg_forest | 46800 | 0.0151824818 |
| 2013 | 16.13 | 1 | 71 | grass_herb | 24300 | 0.0078832117 |
| 2013 | 16.13 | 1 | 81 | pasture_hay | 449100 | 0.1456934307 |
| 2013 | 16.13 | 1 | 82 | crop | 183600 | 0.0595620438 |
| 2013 | 16.13 | 1 | 90 | woody_wetland | 133200 | 0.0432116788 |
| 2013 | 22.13 | 1 | 21 | urban_open | 760500 | 0.247148289 |
| 2013 | 22.13 | 1 | 22 | urban_low | 1295100 | 0.4208832992 |
| 2013 | 22.13 | 1 | 23 | urban_med | 322200 | 0.1047089792 |
| 2013 | 22.13 | 1 | 24 | urban_high | 312300 | 0.1014916642 |
| 2013 | 22.13 | 1 | 41 | dec_forest | 354600 | 0.1152383738 |
| 2013 | 22.13 | 1 | 71 | grass_herb | 22500 | 0.0073120796 |
| 2013 | 22.13 | 1 | 90 | woody_wetland | 9900 | 0.003217315 |
| 2013 | 23.13 | 1 | 21 | urban_open | 235800 | 0.0765410459 |
| 2013 | 23.13 | 1 | 22 | urban_low | 34200 | 0.0111013731 |
| 2013 | 23.13 | 1 | 23 | urban_med | 14400 | 0.0046742623 |
| 2013 | 23.13 | 1 | 24 | urban_high | 10800 | 0.0035056968 |
| 2013 | 23.13 | 1 | 41 | dec_forest | 1201500 | 0.3900087642 |
| 2013 | 23.13 | 1 | 42 | eg_forest | 48600 | 0.0157756354 |
| 2013 | 23.13 | 1 | 71 | grass_herb | 83700 | 0.0271691499 |
| 2013 | 23.13 | 1 | 81 | pasture_hay | 741600 | 0.2407245107 |
| 2013 | 23.13 | 1 | 82 | crop | 676800 | 0.2196903301 |
| 2013 | 23.13 | 1 | 90 | woody_wetland | 33300 | 0.0108092317 |
| 2013 | 26.13 | 1 | 21 | urban_open | 155700 | 0.0505109489 |
| 2013 | 26.13 | 1 | 22 | urban_low | 18000 | 0.0058394161 |
| 2013 | 26.13 | 1 | 41 | dec_forest | 425700 | 0.1381021898 |
| 2013 | 26.13 | 1 | 43 | mix_forest | 12600 | 0.0040875912 |
| 2013 | 26.13 | 1 | 71 | grass_herb | 4500 | 0.001459854 |
| 2013 | 26.13 | 1 | 82 | crop | 2466000 | 0.8 |
| 2013 | 28.13 | 1 | 11 | open_water | 323100 | 0.1049094097 |
| 2013 | 28.13 | 1 | 21 | urban_open | 937800 | 0.3045002922 |
| 2013 | 28.13 | 1 | 22 | urban_low | 763200 | 0.2478082992 |
| 2013 | 28.13 | 1 | 23 | urban_med | 18900 | 0.0061367621 |
| 2013 | 28.13 | 1 | 24 | urban_high | 900 | 0.0002922268 |
| 2013 | 28.13 | 1 | 41 | dec_forest | 676800 | 0.2197545295 |
| 2013 | 28.13 | 1 | 42 | eg_forest | 9900 | 0.0032144944 |
| 2013 | 28.13 | 1 | 71 | grass_herb | 136800 | 0.0444184687 |
| 2013 | 28.13 | 1 | 81 | pasture_hay | 178200 | 0.0578609001 |
| 2013 | 28.13 | 1 | 82 | crop | 34200 | 0.0111046172 |
| 2013 | 36.13 | 1 | 11 | open_water | 88200 | 0.0285797609 |
| 2013 | 36.13 | 1 | 21 | urban_open | 808200 | 0.2618839312 |
| 2013 | 36.13 | 1 | 22 | urban_low | 678600 | 0.2198891805 |
| 2013 | 36.13 | 1 | 23 | urban_med | 512100 | 0.1659375911 |
| 2013 | 36.13 | 1 | 24 | urban_high | 207900 | 0.0673665792 |
| 2013 | 36.13 | 1 | 41 | dec_forest | 730800 | 0.2368037329 |
| 2013 | 36.13 | 1 | 71 | grass_herb | 21600 | 0.0069991251 |
| 2013 | 36.13 | 1 | 81 | pasture_hay | 38700 | 0.0125400992 |
| 2013 | 37.13 | 1 | 11 | open_water | 127800 | 0.0414477525 |
| 2013 | 37.13 | 1 | 21 | urban_open | 618300 | 0.200525394 |
| 2013 | 37.13 | 1 | 22 | urban_low | 738900 | 0.2396380619 |
| 2013 | 37.13 | 1 | 23 | urban_med | 160200 | 0.0519556334 |
| 2013 | 37.13 | 1 | 24 | urban_high | 40500 | 0.0131348511 |
| 2013 | 37.13 | 1 | 41 | dec_forest | 629100 | 0.204028021 |
| 2013 | 37.13 | 1 | 71 | grass_herb | 29700 | 0.0096322242 |
| 2013 | 37.13 | 1 | 81 | pasture_hay | 196200 | 0.0636310566 |
| 2013 | 37.13 | 1 | 82 | crop | 504900 | 0.1637478109 |
| 2013 | 37.13 | 1 | 90 | woody_wetland | 33300 | 0.0107997665 |
| 2013 | 37.13 | 1 | 95 | em_herb_wetland | 4500 | 0.0014594279 |
| 2013 | 38.13 | 1 | 11 | open_water | 20700 | 0.0067310506 |
| 2013 | 38.13 | 1 | 21 | urban_open | 162900 | 0.0529704419 |
| 2013 | 38.13 | 1 | 22 | urban_low | 9000 | 0.0029265438 |
| 2013 | 38.13 | 1 | 41 | dec_forest | 1608300 | 0.5229733685 |
| 2013 | 38.13 | 1 | 42 | eg_forest | 11700 | 0.0038045069 |
| 2013 | 38.13 | 1 | 43 | mix_forest | 5400 | 0.0017559263 |
| 2013 | 38.13 | 1 | 71 | grass_herb | 9000 | 0.0029265438 |
| 2013 | 38.13 | 1 | 81 | pasture_hay | 743400 | 0.2417325139 |
| 2013 | 38.13 | 1 | 82 | crop | 497700 | 0.1618378695 |
| 2013 | 38.13 | 1 | 90 | woody_wetland | 7200 | 0.002341235 |
| 2013 | 39.13 | 1 | 21 | urban_open | 121500 | 0.0394506137 |
| 2013 | 39.13 | 1 | 22 | urban_low | 4500 | 0.0014611338 |
| 2013 | 39.13 | 1 | 41 | dec_forest | 327600 | 0.1063705435 |
| 2013 | 39.13 | 1 | 42 | eg_forest | 900 | 0.0002922268 |
| 2013 | 39.13 | 1 | 52 | scrub | 49500 | 0.0160724722 |
| 2013 | 39.13 | 1 | 81 | pasture_hay | 338400 | 0.1098772648 |
| 2013 | 39.13 | 1 | 82 | crop | 2210400 | 0.7177089421 |
| 2013 | 39.13 | 1 | 90 | woody_wetland | 27000 | 0.008766803 |
| 2013 | 40.13 | 1 | 21 | urban_open | 907200 | 0.2947368421 |
| 2013 | 40.13 | 1 | 22 | urban_low | 1070100 | 0.3476608187 |
| 2013 | 40.13 | 1 | 23 | urban_med | 90000 | 0.0292397661 |
| 2013 | 40.13 | 1 | 24 | urban_high | 53100 | 0.017251462 |
| 2013 | 40.13 | 1 | 41 | dec_forest | 396900 | 0.1289473684 |
| 2013 | 40.13 | 1 | 71 | grass_herb | 14400 | 0.0046783626 |
| 2013 | 40.13 | 1 | 81 | pasture_hay | 97200 | 0.0315789474 |
| 2013 | 40.13 | 1 | 82 | crop | 449100 | 0.1459064327 |
| 2013 | 42.13 | 1 | 21 | urban_open | 114300 | 0.0371562317 |
| 2013 | 42.13 | 1 | 41 | dec_forest | 2390400 | 0.7770626097 |
| 2013 | 42.13 | 1 | 42 | eg_forest | 23400 | 0.0076067876 |
| 2013 | 42.13 | 1 | 52 | scrub | 9000 | 0.0029256875 |
| 2013 | 42.13 | 1 | 71 | grass_herb | 900 | 0.0002925688 |
| 2013 | 42.13 | 1 | 81 | pasture_hay | 538200 | 0.1749561147 |
| 2013 | 43.13 | 1 | 11 | open_water | 683100 | 0.2223198594 |
| 2013 | 43.13 | 1 | 21 | urban_open | 368100 | 0.1198008202 |
| 2013 | 43.13 | 1 | 22 | urban_low | 102600 | 0.0333919156 |
| 2013 | 43.13 | 1 | 23 | urban_med | 17100 | 0.0055653193 |
| 2013 | 43.13 | 1 | 31 | barren | 7200 | 0.0023432923 |
| 2013 | 43.13 | 1 | 41 | dec_forest | 1494000 | 0.4862331576 |
| 2013 | 43.13 | 1 | 42 | eg_forest | 43200 | 0.014059754 |
| 2013 | 43.13 | 1 | 71 | grass_herb | 9000 | 0.0029291154 |
| 2013 | 43.13 | 1 | 81 | pasture_hay | 186300 | 0.0606326889 |
| 2013 | 43.13 | 1 | 82 | crop | 162000 | 0.0527240773 |
| 2013 | 46.13 | 1 | 11 | open_water | 746100 | 0.2432511737 |
| 2013 | 46.13 | 1 | 21 | urban_open | 504900 | 0.1646126761 |
| 2013 | 46.13 | 1 | 22 | urban_low | 144000 | 0.0469483568 |
| 2013 | 46.13 | 1 | 23 | urban_med | 9000 | 0.0029342723 |
| 2013 | 46.13 | 1 | 24 | urban_high | 20700 | 0.0067488263 |
| 2013 | 46.13 | 1 | 41 | dec_forest | 971100 | 0.3166079812 |
| 2013 | 46.13 | 1 | 42 | eg_forest | 16200 | 0.0052816901 |
| 2013 | 46.13 | 1 | 71 | grass_herb | 81900 | 0.0267018779 |
| 2013 | 46.13 | 1 | 81 | pasture_hay | 255600 | 0.0833333333 |
| 2013 | 46.13 | 1 | 82 | crop | 235800 | 0.0768779343 |
| 2013 | 46.13 | 1 | 90 | woody_wetland | 81000 | 0.0264084507 |
| 2013 | 46.13 | 1 | 95 | em_herb_wetland | 900 | 0.0002934272 |
| 2013 | 47.13 | 1 | 21 | urban_open | 180900 | 0.0588579795 |
| 2013 | 47.13 | 1 | 22 | urban_low | 37800 | 0.0122986823 |
| 2013 | 47.13 | 1 | 41 | dec_forest | 1215900 | 0.3956076135 |
| 2013 | 47.13 | 1 | 42 | eg_forest | 77400 | 0.0251830161 |
| 2013 | 47.13 | 1 | 71 | grass_herb | 79200 | 0.0257686676 |
| 2013 | 47.13 | 1 | 81 | pasture_hay | 578700 | 0.1882869693 |
| 2013 | 47.13 | 1 | 82 | crop | 903600 | 0.2939970717 |
| 2013 | 3.13 | 2 | 11 | open_water | 900 | 7.29E-05 |
| 2013 | 3.13 | 2 | 21 | urban_open | 3063600 | 0.2481592185 |
| 2013 | 3.13 | 2 | 22 | urban_low | 5489100 | 0.4446307502 |
| 2013 | 3.13 | 2 | 23 | urban_med | 1267200 | 0.1026463512 |
| 2013 | 3.13 | 2 | 24 | urban_high | 367200 | 0.0297441131 |
| 2013 | 3.13 | 2 | 41 | dec_forest | 1872900 | 0.1517095575 |
| 2013 | 3.13 | 2 | 42 | eg_forest | 23400 | 0.0018954582 |
| 2013 | 3.13 | 2 | 71 | grass_herb | 115200 | 0.0093314865 |
| 2013 | 3.13 | 2 | 81 | pasture_hay | 33300 | 0.0026973828 |
| 2013 | 3.13 | 2 | 90 | woody_wetland | 112500 | 0.0091127798 |
| 2013 | 7.13 | 2 | 11 | open_water | 161100 | 0.0130475982 |
| 2013 | 7.13 | 2 | 21 | urban_open | 1039500 | 0.0841898098 |
| 2013 | 7.13 | 2 | 22 | urban_low | 112500 | 0.0091114513 |
| 2013 | 7.13 | 2 | 23 | urban_med | 28800 | 0.0023325315 |
| 2013 | 7.13 | 2 | 41 | dec_forest | 8250300 | 0.6681973905 |
| 2013 | 7.13 | 2 | 42 | eg_forest | 322200 | 0.0260951964 |
| 2013 | 7.13 | 2 | 43 | mix_forest | 9900 | 0.0008018077 |
| 2013 | 7.13 | 2 | 52 | scrub | 89100 | 0.0072162694 |
| 2013 | 7.13 | 2 | 71 | grass_herb | 129600 | 0.0104963919 |
| 2013 | 7.13 | 2 | 81 | pasture_hay | 1360800 | 0.1102121146 |
| 2013 | 7.13 | 2 | 82 | crop | 843300 | 0.0682994387 |
| 2013 | 11.13 | 2 | 11 | open_water | 62100 | 0.0050276887 |
| 2013 | 11.13 | 2 | 21 | urban_open | 899100 | 0.0727921889 |
| 2013 | 11.13 | 2 | 22 | urban_low | 1936800 | 0.156805596 |
| 2013 | 11.13 | 2 | 23 | urban_med | 214200 | 0.0173418828 |
| 2013 | 11.13 | 2 | 41 | dec_forest | 1693800 | 0.1371320315 |
| 2013 | 11.13 | 2 | 42 | eg_forest | 9900 | 0.0008015156 |
| 2013 | 11.13 | 2 | 43 | mix_forest | 5400 | 0.0004371903 |
| 2013 | 11.13 | 2 | 71 | grass_herb | 90900 | 0.0073593704 |
| 2013 | 11.13 | 2 | 81 | pasture_hay | 1808100 | 0.1463858933 |
| 2013 | 11.13 | 2 | 82 | crop | 5631300 | 0.4559166424 |
| 2013 | 14.13 | 2 | 11 | open_water | 550800 | 0.0446226759 |
| 2013 | 14.13 | 2 | 21 | urban_open | 1137600 | 0.0921618666 |
| 2013 | 14.13 | 2 | 22 | urban_low | 123300 | 0.0099890631 |
| 2013 | 14.13 | 2 | 41 | dec_forest | 7740000 | 0.6270506744 |
| 2013 | 14.13 | 2 | 42 | eg_forest | 6300 | 0.0005103901 |
| 2013 | 14.13 | 2 | 52 | scrub | 23400 | 0.0018957346 |
| 2013 | 14.13 | 2 | 71 | grass_herb | 178200 | 0.0144367481 |
| 2013 | 14.13 | 2 | 81 | pasture_hay | 2203200 | 0.1784907036 |
| 2013 | 14.13 | 2 | 82 | crop | 374400 | 0.0303317536 |
| 2013 | 14.13 | 2 | 90 | woody_wetland | 6300 | 0.0005103901 |
| 2013 | 16.13 | 2 | 11 | open_water | 62100 | 0.005036864 |
| 2013 | 16.13 | 2 | 21 | urban_open | 1660500 | 0.1346813636 |
| 2013 | 16.13 | 2 | 22 | urban_low | 837900 | 0.067961165 |
| 2013 | 16.13 | 2 | 23 | urban_med | 167400 | 0.0135776334 |
| 2013 | 16.13 | 2 | 24 | urban_high | 6300 | 0.0005109862 |
| 2013 | 16.13 | 2 | 41 | dec_forest | 6165900 | 0.500109497 |
| 2013 | 16.13 | 2 | 42 | eg_forest | 188100 | 0.0152565881 |
| 2013 | 16.13 | 2 | 52 | scrub | 4500 | 0.0003649901 |
| 2013 | 16.13 | 2 | 71 | grass_herb | 196200 | 0.0159135703 |
| 2013 | 16.13 | 2 | 81 | pasture_hay | 1637100 | 0.1327834148 |
| 2013 | 16.13 | 2 | 82 | crop | 844200 | 0.0684721513 |
| 2013 | 16.13 | 2 | 90 | woody_wetland | 558900 | 0.045331776 |
| 2013 | 22.13 | 2 | 11 | open_water | 22500 | 0.0018216263 |
| 2013 | 22.13 | 2 | 21 | urban_open | 3466800 | 0.2806761877 |
| 2013 | 22.13 | 2 | 22 | urban_low | 5091300 | 0.41219761 |
| 2013 | 22.13 | 2 | 23 | urban_med | 1150200 | 0.0931215389 |
| 2013 | 22.13 | 2 | 24 | urban_high | 782100 | 0.0633197319 |
| 2013 | 22.13 | 2 | 31 | barren | 20700 | 0.0016758962 |
| 2013 | 22.13 | 2 | 41 | dec_forest | 1589400 | 0.1286796852 |
| 2013 | 22.13 | 2 | 42 | eg_forest | 9900 | 0.0008015156 |
| 2013 | 22.13 | 2 | 71 | grass_herb | 142200 | 0.0115126785 |
| 2013 | 22.13 | 2 | 81 | pasture_hay | 43200 | 0.0034975226 |
| 2013 | 22.13 | 2 | 90 | woody_wetland | 33300 | 0.002696007 |
| 2013 | 23.13 | 2 | 11 | open_water | 27000 | 0.0021865889 |
| 2013 | 23.13 | 2 | 21 | urban_open | 1034100 | 0.0837463557 |
| 2013 | 23.13 | 2 | 22 | urban_low | 292500 | 0.0236880466 |
| 2013 | 23.13 | 2 | 23 | urban_med | 61200 | 0.0049562682 |
| 2013 | 23.13 | 2 | 24 | urban_high | 25200 | 0.0020408163 |
| 2013 | 23.13 | 2 | 41 | dec_forest | 4450500 | 0.3604227405 |
| 2013 | 23.13 | 2 | 42 | eg_forest | 118800 | 0.0096209913 |
| 2013 | 23.13 | 2 | 71 | grass_herb | 257400 | 0.020845481 |
| 2013 | 23.13 | 2 | 81 | pasture_hay | 2934000 | 0.2376093294 |
| 2013 | 23.13 | 2 | 82 | crop | 2962800 | 0.239941691 |
| 2013 | 23.13 | 2 | 90 | woody_wetland | 184500 | 0.014941691 |
| 2013 | 26.13 | 2 | 11 | open_water | 54000 | 0.0043731778 |
| 2013 | 26.13 | 2 | 21 | urban_open | 621900 | 0.0503644315 |
| 2013 | 26.13 | 2 | 22 | urban_low | 126900 | 0.0102769679 |
| 2013 | 26.13 | 2 | 41 | dec_forest | 1790100 | 0.1449708455 |
| 2013 | 26.13 | 2 | 43 | mix_forest | 57600 | 0.004664723 |
| 2013 | 26.13 | 2 | 71 | grass_herb | 32400 | 0.0026239067 |
| 2013 | 26.13 | 2 | 81 | pasture_hay | 900 | 7.29E-05 |
| 2013 | 26.13 | 2 | 82 | crop | 9658800 | 0.7822157434 |
| 2013 | 26.13 | 2 | 90 | woody_wetland | 5400 | 0.0004373178 |
| 2013 | 28.13 | 2 | 11 | open_water | 2594700 | 0.2102537923 |
| 2013 | 28.13 | 2 | 21 | urban_open | 3296700 | 0.267138273 |
| 2013 | 28.13 | 2 | 22 | urban_low | 2403000 | 0.1947199533 |
| 2013 | 28.13 | 2 | 23 | urban_med | 95400 | 0.0077304551 |
| 2013 | 28.13 | 2 | 24 | urban_high | 43200 | 0.0035005834 |
| 2013 | 28.13 | 2 | 41 | dec_forest | 2629800 | 0.2130980163 |
| 2013 | 28.13 | 2 | 42 | eg_forest | 56700 | 0.0045945158 |
| 2013 | 28.13 | 2 | 71 | grass_herb | 306000 | 0.0247957993 |
| 2013 | 28.13 | 2 | 81 | pasture_hay | 590400 | 0.0478413069 |
| 2013 | 28.13 | 2 | 82 | crop | 239400 | 0.0193990665 |
| 2013 | 28.13 | 2 | 90 | woody_wetland | 81000 | 0.0065635939 |
| 2013 | 28.13 | 2 | 95 | em_herb_wetland | 4500 | 0.0003646441 |
| 2013 | 36.13 | 2 | 11 | open_water | 619200 | 0.0501238525 |
| 2013 | 36.13 | 2 | 21 | urban_open | 2798100 | 0.2265044441 |
| 2013 | 36.13 | 2 | 22 | urban_low | 2395800 | 0.1939385109 |
| 2013 | 36.13 | 2 | 23 | urban_med | 1702800 | 0.1378405945 |
| 2013 | 36.13 | 2 | 24 | urban_high | 1260000 | 0.1019962116 |
| 2013 | 36.13 | 2 | 41 | dec_forest | 3241800 | 0.2624216815 |
| 2013 | 36.13 | 2 | 42 | eg_forest | 18000 | 0.0014570887 |
| 2013 | 36.13 | 2 | 52 | scrub | 11700 | 0.0009471077 |
| 2013 | 36.13 | 2 | 71 | grass_herb | 208800 | 0.0169022293 |
| 2013 | 36.13 | 2 | 81 | pasture_hay | 92700 | 0.007504007 |
| 2013 | 36.13 | 2 | 90 | woody_wetland | 4500 | 0.0003642722 |
| 2013 | 37.13 | 2 | 11 | open_water | 319500 | 0.0258595571 |
| 2013 | 37.13 | 2 | 21 | urban_open | 1542600 | 0.1248543124 |
| 2013 | 37.13 | 2 | 22 | urban_low | 1479600 | 0.1197552448 |
| 2013 | 37.13 | 2 | 23 | urban_med | 405900 | 0.0328525641 |
| 2013 | 37.13 | 2 | 24 | urban_high | 87300 | 0.0070658508 |
| 2013 | 37.13 | 2 | 31 | barren | 16200 | 0.0013111888 |
| 2013 | 37.13 | 2 | 41 | dec_forest | 4280400 | 0.3464452214 |
| 2013 | 37.13 | 2 | 42 | eg_forest | 45000 | 0.0036421911 |
| 2013 | 37.13 | 2 | 71 | grass_herb | 212400 | 0.0171911422 |
| 2013 | 37.13 | 2 | 81 | pasture_hay | 1207800 | 0.0977564103 |
| 2013 | 37.13 | 2 | 82 | crop | 2550600 | 0.2064393939 |
| 2013 | 37.13 | 2 | 90 | woody_wetland | 198000 | 0.016025641 |
| 2013 | 37.13 | 2 | 95 | em_herb_wetland | 9900 | 0.0008012821 |
| 2013 | 38.13 | 2 | 11 | open_water | 74700 | 0.0060473588 |
| 2013 | 38.13 | 2 | 21 | urban_open | 705600 | 0.0571220401 |
| 2013 | 38.13 | 2 | 22 | urban_low | 9000 | 0.0007285974 |
| 2013 | 38.13 | 2 | 41 | dec_forest | 8862300 | 0.7174499089 |
| 2013 | 38.13 | 2 | 42 | eg_forest | 32400 | 0.0026229508 |
| 2013 | 38.13 | 2 | 43 | mix_forest | 5400 | 0.0004371585 |
| 2013 | 38.13 | 2 | 71 | grass_herb | 76500 | 0.0061930783 |
| 2013 | 38.13 | 2 | 81 | pasture_hay | 1442700 | 0.1167941712 |
| 2013 | 38.13 | 2 | 82 | crop | 1136700 | 0.0920218579 |
| 2013 | 38.13 | 2 | 90 | woody_wetland | 7200 | 0.000582878 |
| 2013 | 39.13 | 2 | 11 | open_water | 6300 | 0.0005107252 |
| 2013 | 39.13 | 2 | 21 | urban_open | 529200 | 0.0429009193 |
| 2013 | 39.13 | 2 | 22 | urban_low | 46800 | 0.0037939589 |
| 2013 | 39.13 | 2 | 41 | dec_forest | 1996200 | 0.1618269371 |
| 2013 | 39.13 | 2 | 42 | eg_forest | 16200 | 0.0013132934 |
| 2013 | 39.13 | 2 | 52 | scrub | 93600 | 0.0075879177 |
| 2013 | 39.13 | 2 | 71 | grass_herb | 21600 | 0.0017510579 |
| 2013 | 39.13 | 2 | 81 | pasture_hay | 1176300 | 0.0953596965 |
| 2013 | 39.13 | 2 | 82 | crop | 8386200 | 0.6798482416 |
| 2013 | 39.13 | 2 | 90 | woody_wetland | 63000 | 0.0051072523 |
| 2013 | 40.13 | 2 | 11 | open_water | 42300 | 0.0034296556 |
| 2013 | 40.13 | 2 | 21 | urban_open | 2547000 | 0.2065090485 |
| 2013 | 40.13 | 2 | 22 | urban_low | 2776500 | 0.2251167542 |
| 2013 | 40.13 | 2 | 23 | urban_med | 367200 | 0.0297723292 |
| 2013 | 40.13 | 2 | 24 | urban_high | 159300 | 0.012915937 |
| 2013 | 40.13 | 2 | 41 | dec_forest | 2324700 | 0.1884851138 |
| 2013 | 40.13 | 2 | 71 | grass_herb | 47700 | 0.0038674839 |
| 2013 | 40.13 | 2 | 81 | pasture_hay | 1183500 | 0.0959573847 |
| 2013 | 40.13 | 2 | 82 | crop | 2736900 | 0.2219060128 |
| 2013 | 40.13 | 2 | 90 | woody_wetland | 148500 | 0.0120402802 |
| 2013 | 42.13 | 2 | 21 | urban_open | 586800 | 0.0474907131 |
| 2013 | 42.13 | 2 | 22 | urban_low | 22500 | 0.0018209629 |
| 2013 | 42.13 | 2 | 23 | urban_med | 9000 | 0.0007283852 |
| 2013 | 42.13 | 2 | 31 | barren | 6300 | 0.0005098696 |
| 2013 | 42.13 | 2 | 41 | dec_forest | 8836200 | 0.71512856 |
| 2013 | 42.13 | 2 | 42 | eg_forest | 117000 | 0.0094690072 |
| 2013 | 42.13 | 2 | 52 | scrub | 90000 | 0.0072838517 |
| 2013 | 42.13 | 2 | 71 | grass_herb | 117900 | 0.0095418457 |
| 2013 | 42.13 | 2 | 81 | pasture_hay | 2570400 | 0.2080268046 |
| 2013 | 43.13 | 2 | 11 | open_water | 2989800 | 0.2419519301 |
| 2013 | 43.13 | 2 | 21 | urban_open | 1350000 | 0.1092498179 |
| 2013 | 43.13 | 2 | 22 | urban_low | 834300 | 0.0675163875 |
| 2013 | 43.13 | 2 | 23 | urban_med | 245700 | 0.0198834669 |
| 2013 | 43.13 | 2 | 24 | urban_high | 36000 | 0.0029133285 |
| 2013 | 43.13 | 2 | 31 | barren | 7200 | 0.0005826657 |
| 2013 | 43.13 | 2 | 41 | dec_forest | 4392000 | 0.3554260743 |
| 2013 | 43.13 | 2 | 42 | eg_forest | 162000 | 0.0131099782 |
| 2013 | 43.13 | 2 | 52 | scrub | 12600 | 0.001019665 |
| 2013 | 43.13 | 2 | 71 | grass_herb | 60300 | 0.0048798252 |
| 2013 | 43.13 | 2 | 81 | pasture_hay | 1088100 | 0.0880553532 |
| 2013 | 43.13 | 2 | 82 | crop | 1162800 | 0.0941005098 |
| 2013 | 43.13 | 2 | 90 | woody_wetland | 5400 | 0.0004369993 |
| 2013 | 43.13 | 2 | 95 | em_herb_wetland | 10800 | 0.0008739985 |
| 2013 | 46.13 | 2 | 11 | open_water | 1767600 | 0.1429923553 |
| 2013 | 46.13 | 2 | 21 | urban_open | 1867500 | 0.1510738988 |
| 2013 | 46.13 | 2 | 22 | urban_low | 533700 | 0.043174372 |
| 2013 | 46.13 | 2 | 23 | urban_med | 48600 | 0.0039315617 |
| 2013 | 46.13 | 2 | 24 | urban_high | 33300 | 0.0026938478 |
| 2013 | 46.13 | 2 | 41 | dec_forest | 3199500 | 0.2588278122 |
| 2013 | 46.13 | 2 | 42 | eg_forest | 88200 | 0.0071350564 |
| 2013 | 46.13 | 2 | 43 | mix_forest | 7200 | 0.0005824536 |
| 2013 | 46.13 | 2 | 71 | grass_herb | 305100 | 0.0246814707 |
| 2013 | 46.13 | 2 | 81 | pasture_hay | 2460600 | 0.1990535129 |
| 2013 | 46.13 | 2 | 82 | crop | 1755000 | 0.1419730615 |
| 2013 | 46.13 | 2 | 90 | woody_wetland | 275400 | 0.0222788497 |
| 2013 | 46.13 | 2 | 95 | em_herb_wetland | 19800 | 0.0016017474 |
| 2013 | 47.13 | 2 | 11 | open_water | 48600 | 0.0039358601 |
| 2013 | 47.13 | 2 | 21 | urban_open | 797400 | 0.0645772595 |
| 2013 | 47.13 | 2 | 22 | urban_low | 139500 | 0.0112973761 |
| 2013 | 47.13 | 2 | 23 | urban_med | 14400 | 0.0011661808 |
| 2013 | 47.13 | 2 | 41 | dec_forest | 3734100 | 0.3024052478 |
| 2013 | 47.13 | 2 | 42 | eg_forest | 224100 | 0.018148688 |
| 2013 | 47.13 | 2 | 43 | mix_forest | 4500 | 0.0003644315 |
| 2013 | 47.13 | 2 | 71 | grass_herb | 238500 | 0.0193148688 |
| 2013 | 47.13 | 2 | 81 | pasture_hay | 3039300 | 0.2461370262 |
| 2013 | 47.13 | 2 | 82 | crop | 4088700 | 0.331122449 |
| 2013 | 47.13 | 2 | 90 | woody_wetland | 18900 | 0.0015306122 |
| 2013 | 3.13 | 3 | 11 | open_water | 54900 | 0.0019734714 |
| 2013 | 3.13 | 3 | 21 | urban_open | 7444800 | 0.2676156584 |
| 2013 | 3.13 | 3 | 22 | urban_low | 10701000 | 0.3846651569 |
| 2013 | 3.13 | 3 | 23 | urban_med | 2662200 | 0.0956971854 |
| 2013 | 3.13 | 3 | 24 | urban_high | 614700 | 0.0220964089 |
| 2013 | 3.13 | 3 | 41 | dec_forest | 5704200 | 0.2050469104 |
| 2013 | 3.13 | 3 | 42 | eg_forest | 29700 | 0.0010676157 |
| 2013 | 3.13 | 3 | 71 | grass_herb | 264600 | 0.009511485 |
| 2013 | 3.13 | 3 | 81 | pasture_hay | 100800 | 0.0036234228 |
| 2013 | 3.13 | 3 | 90 | woody_wetland | 229500 | 0.0082497574 |
| 2013 | 3.13 | 3 | 95 | em_herb_wetland | 12600 | 0.0004529279 |
| 2013 | 7.13 | 3 | 11 | open_water | 243000 | 0.0087353198 |
| 2013 | 7.13 | 3 | 21 | urban_open | 2862000 | 0.1028826555 |
| 2013 | 7.13 | 3 | 22 | urban_low | 1568700 | 0.0563913423 |
| 2013 | 7.13 | 3 | 23 | urban_med | 387000 | 0.0139118056 |
| 2013 | 7.13 | 3 | 24 | urban_high | 21600 | 0.0007764729 |
| 2013 | 7.13 | 3 | 31 | barren | 8100 | 0.0002911773 |
| 2013 | 7.13 | 3 | 41 | dec_forest | 17619300 | 0.6333753923 |
| 2013 | 7.13 | 3 | 42 | eg_forest | 366300 | 0.0131676858 |
| 2013 | 7.13 | 3 | 43 | mix_forest | 45900 | 0.0016500049 |
| 2013 | 7.13 | 3 | 52 | scrub | 176400 | 0.0063411951 |
| 2013 | 7.13 | 3 | 71 | grass_herb | 634500 | 0.0228088906 |
| 2013 | 7.13 | 3 | 81 | pasture_hay | 2488500 | 0.0894561455 |
| 2013 | 7.13 | 3 | 82 | crop | 1396800 | 0.0502119124 |
| 2013 | 11.13 | 3 | 11 | open_water | 330300 | 0.0118793293 |
| 2013 | 11.13 | 3 | 21 | urban_open | 2386800 | 0.0858419111 |
| 2013 | 11.13 | 3 | 22 | urban_low | 3663000 | 0.1317407911 |
| 2013 | 11.13 | 3 | 23 | urban_med | 660600 | 0.0237586586 |
| 2013 | 11.13 | 3 | 24 | urban_high | 84600 | 0.003042662 |
| 2013 | 11.13 | 3 | 31 | barren | 6300 | 0.0002265812 |
| 2013 | 11.13 | 3 | 41 | dec_forest | 3657600 | 0.1315465786 |
| 2013 | 11.13 | 3 | 42 | eg_forest | 9900 | 0.0003560562 |
| 2013 | 11.13 | 3 | 43 | mix_forest | 41400 | 0.0014889623 |
| 2013 | 11.13 | 3 | 71 | grass_herb | 231300 | 0.0083187674 |
| 2013 | 11.13 | 3 | 81 | pasture_hay | 3263400 | 0.1173690684 |
| 2013 | 11.13 | 3 | 82 | crop | 13469400 | 0.4844306338 |
| 2013 | 14.13 | 3 | 11 | open_water | 813600 | 0.0292660818 |
| 2013 | 14.13 | 3 | 21 | urban_open | 2225700 | 0.0800608631 |
| 2013 | 14.13 | 3 | 22 | urban_low | 237600 | 0.0085467318 |
| 2013 | 14.13 | 3 | 23 | urban_med | 5400 | 0.0001942439 |
| 2013 | 14.13 | 3 | 41 | dec_forest | 19192500 | 0.6903752145 |
| 2013 | 14.13 | 3 | 42 | eg_forest | 10800 | 0.0003884878 |
| 2013 | 14.13 | 3 | 52 | scrub | 106200 | 0.0038201301 |
| 2013 | 14.13 | 3 | 71 | grass_herb | 548100 | 0.0197157564 |
| 2013 | 14.13 | 3 | 81 | pasture_hay | 3627000 | 0.1304671566 |
| 2013 | 14.13 | 3 | 82 | crop | 1026900 | 0.036938716 |
| 2013 | 14.13 | 3 | 90 | woody_wetland | 6300 | 0.0002266179 |
| 2013 | 16.13 | 3 | 11 | open_water | 232200 | 0.008355734 |
| 2013 | 16.13 | 3 | 21 | urban_open | 5203800 | 0.1872591249 |
| 2013 | 16.13 | 3 | 22 | urban_low | 2847600 | 0.102471095 |
| 2013 | 16.13 | 3 | 23 | urban_med | 433800 | 0.0156103248 |
| 2013 | 16.13 | 3 | 24 | urban_high | 57600 | 0.0020727402 |
| 2013 | 16.13 | 3 | 31 | barren | 16200 | 0.0005829582 |
| 2013 | 16.13 | 3 | 41 | dec_forest | 10433700 | 0.3754574602 |
| 2013 | 16.13 | 3 | 42 | eg_forest | 387900 | 0.01395861 |
| 2013 | 16.13 | 3 | 43 | mix_forest | 4500 | 0.0001619328 |
| 2013 | 16.13 | 3 | 52 | scrub | 47700 | 0.001716488 |
| 2013 | 16.13 | 3 | 71 | grass_herb | 360900 | 0.012987013 |
| 2013 | 16.13 | 3 | 81 | pasture_hay | 3501900 | 0.1260161285 |
| 2013 | 16.13 | 3 | 82 | crop | 3264300 | 0.1174660751 |
| 2013 | 16.13 | 3 | 90 | woody_wetland | 997200 | 0.0358843152 |
| 2013 | 22.13 | 3 | 11 | open_water | 127800 | 0.0045974034 |
| 2013 | 22.13 | 3 | 21 | urban_open | 7756200 | 0.2790170622 |
| 2013 | 22.13 | 3 | 22 | urban_low | 8806500 | 0.3167999482 |
| 2013 | 22.13 | 3 | 23 | urban_med | 2511000 | 0.0903292647 |
| 2013 | 22.13 | 3 | 24 | urban_high | 1131300 | 0.0406967333 |
| 2013 | 22.13 | 3 | 31 | barren | 20700 | 0.0007446499 |
| 2013 | 22.13 | 3 | 41 | dec_forest | 5982300 | 0.2152038074 |
| 2013 | 22.13 | 3 | 42 | eg_forest | 179100 | 0.00644284 |
| 2013 | 22.13 | 3 | 71 | grass_herb | 366300 | 0.0131770648 |
| 2013 | 22.13 | 3 | 81 | pasture_hay | 567000 | 0.0203969307 |
| 2013 | 22.13 | 3 | 90 | woody_wetland | 345600 | 0.0124324149 |
| 2013 | 22.13 | 3 | 95 | em_herb_wetland | 4500 | 0.0001618804 |
| 2013 | 23.13 | 3 | 11 | open_water | 43200 | 0.0015545552 |
| 2013 | 23.13 | 3 | 21 | urban_open | 2433600 | 0.0875732746 |
| 2013 | 23.13 | 3 | 22 | urban_low | 543600 | 0.0195614859 |
| 2013 | 23.13 | 3 | 23 | urban_med | 67500 | 0.0024289925 |
| 2013 | 23.13 | 3 | 24 | urban_high | 25200 | 0.0009068238 |
| 2013 | 23.13 | 3 | 41 | dec_forest | 8650800 | 0.3112996729 |
| 2013 | 23.13 | 3 | 42 | eg_forest | 170100 | 0.006121061 |
| 2013 | 23.13 | 3 | 71 | grass_herb | 416700 | 0.0149949801 |
| 2013 | 23.13 | 3 | 81 | pasture_hay | 7421400 | 0.2670596237 |
| 2013 | 23.13 | 3 | 82 | crop | 7766100 | 0.2794636785 |
| 2013 | 23.13 | 3 | 90 | woody_wetland | 251100 | 0.0090358519 |
| 2013 | 26.13 | 3 | 11 | open_water | 66600 | 0.0023937375 |
| 2013 | 26.13 | 3 | 21 | urban_open | 1285200 | 0.0461926635 |
| 2013 | 26.13 | 3 | 22 | urban_low | 263700 | 0.0094779065 |
| 2013 | 26.13 | 3 | 31 | barren | 4500 | 0.000161739 |
| 2013 | 26.13 | 3 | 41 | dec_forest | 3608100 | 0.1296823446 |
| 2013 | 26.13 | 3 | 42 | eg_forest | 13500 | 0.0004852171 |
| 2013 | 26.13 | 3 | 43 | mix_forest | 128700 | 0.0046257359 |
| 2013 | 26.13 | 3 | 71 | grass_herb | 36900 | 0.0013262599 |
| 2013 | 26.13 | 3 | 81 | pasture_hay | 246600 | 0.0088632982 |
| 2013 | 26.13 | 3 | 82 | crop | 22153500 | 0.7962411852 |
| 2013 | 26.13 | 3 | 90 | woody_wetland | 15300 | 0.0005499127 |
| 2013 | 28.13 | 3 | 11 | open_water | 4625100 | 0.1663322113 |
| 2013 | 28.13 | 3 | 21 | urban_open | 7451100 | 0.2679634904 |
| 2013 | 28.13 | 3 | 22 | urban_low | 4468500 | 0.1607004143 |
| 2013 | 28.13 | 3 | 23 | urban_med | 323100 | 0.0116196271 |
| 2013 | 28.13 | 3 | 24 | urban_high | 91800 | 0.0033013982 |
| 2013 | 28.13 | 3 | 41 | dec_forest | 7056900 | 0.253786898 |
| 2013 | 28.13 | 3 | 42 | eg_forest | 401400 | 0.0144355256 |
| 2013 | 28.13 | 3 | 71 | grass_herb | 909900 | 0.0327226825 |
| 2013 | 28.13 | 3 | 81 | pasture_hay | 1498500 | 0.0538904713 |
| 2013 | 28.13 | 3 | 82 | crop | 779400 | 0.0280295184 |
| 2013 | 28.13 | 3 | 90 | woody_wetland | 196200 | 0.0070559296 |
| 2013 | 28.13 | 3 | 95 | em_herb_wetland | 4500 | 0.0001618332 |
| 2013 | 36.13 | 3 | 11 | open_water | 1003500 | 0.0360911504 |
| 2013 | 36.13 | 3 | 21 | urban_open | 5718600 | 0.2056710041 |
| 2013 | 36.13 | 3 | 22 | urban_low | 5965200 | 0.2145400401 |
| 2013 | 36.13 | 3 | 23 | urban_med | 2873700 | 0.103353402 |
| 2013 | 36.13 | 3 | 24 | urban_high | 1667700 | 0.059979284 |
| 2013 | 36.13 | 3 | 41 | dec_forest | 7758900 | 0.2790509484 |
| 2013 | 36.13 | 3 | 42 | eg_forest | 97200 | 0.0034958244 |
| 2013 | 36.13 | 3 | 52 | scrub | 42300 | 0.001521331 |
| 2013 | 36.13 | 3 | 71 | grass_herb | 418500 | 0.0150514663 |
| 2013 | 36.13 | 3 | 81 | pasture_hay | 2130300 | 0.0766168188 |
| 2013 | 36.13 | 3 | 82 | crop | 124200 | 0.0044668868 |
| 2013 | 36.13 | 3 | 90 | woody_wetland | 4500 | 0.0001618437 |
| 2013 | 37.13 | 3 | 11 | open_water | 552600 | 0.0198956612 |
| 2013 | 37.13 | 3 | 21 | urban_open | 2780100 | 0.1000939697 |
| 2013 | 37.13 | 3 | 22 | urban_low | 2325600 | 0.0837302745 |
| 2013 | 37.13 | 3 | 23 | urban_med | 639900 | 0.0230387868 |
| 2013 | 37.13 | 3 | 24 | urban_high | 178200 | 0.0064158647 |
| 2013 | 37.13 | 3 | 31 | barren | 260100 | 0.0093645702 |
| 2013 | 37.13 | 3 | 41 | dec_forest | 11066400 | 0.3984316775 |
| 2013 | 37.13 | 3 | 42 | eg_forest | 145800 | 0.0052493438 |
| 2013 | 37.13 | 3 | 71 | grass_herb | 497700 | 0.0179190564 |
| 2013 | 37.13 | 3 | 81 | pasture_hay | 2833200 | 0.1020057678 |
| 2013 | 37.13 | 3 | 82 | crop | 6020100 | 0.2167460549 |
| 2013 | 37.13 | 3 | 90 | woody_wetland | 458100 | 0.0164933087 |
| 2013 | 37.13 | 3 | 95 | em_herb_wetland | 17100 | 0.0006156638 |
| 2013 | 38.13 | 3 | 11 | open_water | 110700 | 0.0039823868 |
| 2013 | 38.13 | 3 | 21 | urban_open | 1481400 | 0.053292754 |
| 2013 | 38.13 | 3 | 22 | urban_low | 9000 | 0.0003237713 |
| 2013 | 38.13 | 3 | 31 | barren | 43200 | 0.0015541022 |
| 2013 | 38.13 | 3 | 41 | dec_forest | 20724300 | 0.7455481448 |
| 2013 | 38.13 | 3 | 42 | eg_forest | 121500 | 0.0043709124 |
| 2013 | 38.13 | 3 | 43 | mix_forest | 5400 | 0.0001942628 |
| 2013 | 38.13 | 3 | 52 | scrub | 4500 | 0.0001618856 |
| 2013 | 38.13 | 3 | 71 | grass_herb | 250200 | 0.0090008418 |
| 2013 | 38.13 | 3 | 81 | pasture_hay | 2819700 | 0.1014375445 |
| 2013 | 38.13 | 3 | 82 | crop | 2220300 | 0.0798743767 |
| 2013 | 38.13 | 3 | 90 | woody_wetland | 7200 | 0.000259017 |
| 2013 | 39.13 | 3 | 11 | open_water | 253800 | 0.0091341949 |
| 2013 | 39.13 | 3 | 21 | urban_open | 1346400 | 0.0484565802 |
| 2013 | 39.13 | 3 | 22 | urban_low | 339300 | 0.0122113173 |
| 2013 | 39.13 | 3 | 23 | urban_med | 59400 | 0.0021377903 |
| 2013 | 39.13 | 3 | 41 | dec_forest | 3781800 | 0.1361059826 |
| 2013 | 39.13 | 3 | 42 | eg_forest | 16200 | 0.0005830337 |
| 2013 | 39.13 | 3 | 43 | mix_forest | 5400 | 0.0001943446 |
| 2013 | 39.13 | 3 | 52 | scrub | 158400 | 0.0057007741 |
| 2013 | 39.13 | 3 | 71 | grass_herb | 91800 | 0.0033038577 |
| 2013 | 39.13 | 3 | 81 | pasture_hay | 2824200 | 0.1016422116 |
| 2013 | 39.13 | 3 | 82 | crop | 18829800 | 0.6776795258 |
| 2013 | 39.13 | 3 | 90 | woody_wetland | 79200 | 0.0028503871 |
| 2013 | 40.13 | 3 | 11 | open_water | 49500 | 0.0017807421 |
| 2013 | 40.13 | 3 | 21 | urban_open | 4811400 | 0.1730881305 |
| 2013 | 40.13 | 3 | 22 | urban_low | 5767200 | 0.2074726413 |
| 2013 | 40.13 | 3 | 23 | urban_med | 1617300 | 0.0581817004 |
| 2013 | 40.13 | 3 | 24 | urban_high | 501300 | 0.0180340607 |
| 2013 | 40.13 | 3 | 41 | dec_forest | 4651200 | 0.1673250016 |
| 2013 | 40.13 | 3 | 42 | eg_forest | 43200 | 0.0015541022 |
| 2013 | 40.13 | 3 | 71 | grass_herb | 156600 | 0.0056336204 |
| 2013 | 40.13 | 3 | 81 | pasture_hay | 3528900 | 0.126950722 |
| 2013 | 40.13 | 3 | 82 | crop | 6425100 | 0.2311403225 |
| 2013 | 40.13 | 3 | 90 | woody_wetland | 245700 | 0.0088389562 |
| 2013 | 42.13 | 3 | 21 | urban_open | 1227600 | 0.0441953148 |
| 2013 | 42.13 | 3 | 22 | urban_low | 40500 | 0.0014580566 |
| 2013 | 42.13 | 3 | 23 | urban_med | 9000 | 0.0003240126 |
| 2013 | 42.13 | 3 | 31 | barren | 6300 | 0.0002268088 |
| 2013 | 42.13 | 3 | 41 | dec_forest | 20916000 | 0.7530052166 |
| 2013 | 42.13 | 3 | 42 | eg_forest | 287100 | 0.010336001 |
| 2013 | 42.13 | 3 | 52 | scrub | 273600 | 0.0098499822 |
| 2013 | 42.13 | 3 | 71 | grass_herb | 250200 | 0.0090075495 |
| 2013 | 42.13 | 3 | 81 | pasture_hay | 4766400 | 0.171597058 |
| 2013 | 43.13 | 3 | 11 | open_water | 4343400 | 0.1562722622 |
| 2013 | 43.13 | 3 | 21 | urban_open | 3882600 | 0.1396930251 |
| 2013 | 43.13 | 3 | 22 | urban_low | 3737700 | 0.1344796321 |
| 2013 | 43.13 | 3 | 23 | urban_med | 911700 | 0.0328022796 |
| 2013 | 43.13 | 3 | 24 | urban_high | 107100 | 0.0038533774 |
| 2013 | 43.13 | 3 | 31 | barren | 7200 | 0.0002590506 |
| 2013 | 43.13 | 3 | 41 | dec_forest | 7915500 | 0.284793731 |
| 2013 | 43.13 | 3 | 42 | eg_forest | 208800 | 0.0075124668 |
| 2013 | 43.13 | 3 | 52 | scrub | 254700 | 0.0091639143 |
| 2013 | 43.13 | 3 | 71 | grass_herb | 150300 | 0.0054076808 |
| 2013 | 43.13 | 3 | 81 | pasture_hay | 2144700 | 0.0771646914 |
| 2013 | 43.13 | 3 | 82 | crop | 4113900 | 0.1480150249 |
| 2013 | 43.13 | 3 | 90 | woody_wetland | 5400 | 0.0001942879 |
| 2013 | 43.13 | 3 | 95 | em_herb_wetland | 10800 | 0.0003885759 |
| 2013 | 46.13 | 3 | 11 | open_water | 1815300 | 0.0653279352 |
| 2013 | 46.13 | 3 | 21 | urban_open | 5908500 | 0.2126315789 |
| 2013 | 46.13 | 3 | 22 | urban_low | 2838600 | 0.1021538462 |
| 2013 | 46.13 | 3 | 23 | urban_med | 237600 | 0.0085506073 |
| 2013 | 46.13 | 3 | 24 | urban_high | 99900 | 0.0035951417 |
| 2013 | 46.13 | 3 | 41 | dec_forest | 6513300 | 0.2343967611 |
| 2013 | 46.13 | 3 | 42 | eg_forest | 144900 | 0.0052145749 |
| 2013 | 46.13 | 3 | 43 | mix_forest | 11700 | 0.0004210526 |
| 2013 | 46.13 | 3 | 52 | scrub | 30600 | 0.0011012146 |
| 2013 | 46.13 | 3 | 71 | grass_herb | 663300 | 0.0238704453 |
| 2013 | 46.13 | 3 | 81 | pasture_hay | 5086800 | 0.1830607287 |
| 2013 | 46.13 | 3 | 82 | crop | 3723300 | 0.1339919028 |
| 2013 | 46.13 | 3 | 90 | woody_wetland | 684000 | 0.0246153846 |
| 2013 | 46.13 | 3 | 95 | em_herb_wetland | 29700 | 0.0010688259 |
| 2013 | 47.13 | 3 | 11 | open_water | 171900 | 0.0061848326 |
| 2013 | 47.13 | 3 | 21 | urban_open | 2307600 | 0.0830257108 |
| 2013 | 47.13 | 3 | 22 | urban_low | 461700 | 0.0166116184 |
| 2013 | 47.13 | 3 | 23 | urban_med | 72000 | 0.0025905058 |
| 2013 | 47.13 | 3 | 24 | urban_high | 16200 | 0.0005828638 |
| 2013 | 47.13 | 3 | 41 | dec_forest | 7835400 | 0.2819117933 |
| 2013 | 47.13 | 3 | 42 | eg_forest | 335700 | 0.0120782333 |
| 2013 | 47.13 | 3 | 43 | mix_forest | 4500 | 0.0001619066 |
| 2013 | 47.13 | 3 | 52 | scrub | 5400 | 0.0001942879 |
| 2013 | 47.13 | 3 | 71 | grass_herb | 606600 | 0.0218250113 |
| 2013 | 47.13 | 3 | 81 | pasture_hay | 7308900 | 0.2629687196 |
| 2013 | 47.13 | 3 | 82 | crop | 8629200 | 0.3104721197 |
| 2013 | 47.13 | 3 | 90 | woody_wetland | 31500 | 0.0011333463 |
| 2013 | 47.13 | 3 | 95 | em_herb_wetland | 7200 | 0.0002590506 |
| 2013 | 3.13 | 4 | 11 | open_water | 276300 | 0.0055923929 |
| 2013 | 3.13 | 4 | 21 | urban_open | 12255300 | 0.2480508598 |
| 2013 | 3.13 | 4 | 22 | urban_low | 17106300 | 0.34623652 |
| 2013 | 3.13 | 4 | 23 | urban_med | 5593500 | 0.1132140775 |
| 2013 | 3.13 | 4 | 24 | urban_high | 1316700 | 0.0266503935 |
| 2013 | 3.13 | 4 | 31 | barren | 82800 | 0.0016758962 |
| 2013 | 3.13 | 4 | 41 | dec_forest | 10531800 | 0.2131667152 |
| 2013 | 3.13 | 4 | 42 | eg_forest | 71100 | 0.0014390848 |
| 2013 | 3.13 | 4 | 43 | mix_forest | 2700 | 5.46E-05 |
| 2013 | 3.13 | 4 | 71 | grass_herb | 930600 | 0.0188356164 |
| 2013 | 3.13 | 4 | 81 | pasture_hay | 480600 | 0.0097274847 |
| 2013 | 3.13 | 4 | 82 | crop | 52200 | 0.0010565433 |
| 2013 | 3.13 | 4 | 90 | woody_wetland | 598500 | 0.0121138152 |
| 2013 | 3.13 | 4 | 95 | em_herb_wetland | 108000 | 0.0021859516 |
| 2013 | 7.13 | 4 | 11 | open_water | 491400 | 0.0099464422 |
| 2013 | 7.13 | 4 | 21 | urban_open | 5332500 | 0.1079352935 |
| 2013 | 7.13 | 4 | 22 | urban_low | 2822400 | 0.0571282836 |
| 2013 | 7.13 | 4 | 23 | urban_med | 576000 | 0.0116588334 |
| 2013 | 7.13 | 4 | 24 | urban_high | 34200 | 0.0006922432 |
| 2013 | 7.13 | 4 | 31 | barren | 21600 | 0.0004372063 |
| 2013 | 7.13 | 4 | 41 | dec_forest | 32393700 | 0.6556818596 |
| 2013 | 7.13 | 4 | 42 | eg_forest | 473400 | 0.0095821037 |
| 2013 | 7.13 | 4 | 43 | mix_forest | 92700 | 0.0018763435 |
| 2013 | 7.13 | 4 | 52 | scrub | 411300 | 0.0083251357 |
| 2013 | 7.13 | 4 | 71 | grass_herb | 906300 | 0.0183444457 |
| 2013 | 7.13 | 4 | 81 | pasture_hay | 4341600 | 0.0878784567 |
| 2013 | 7.13 | 4 | 82 | crop | 1507500 | 0.030513353 |
| 2013 | 11.13 | 4 | 11 | open_water | 1074600 | 0.0217347775 |
| 2013 | 11.13 | 4 | 21 | urban_open | 5132700 | 0.1038135979 |
| 2013 | 11.13 | 4 | 22 | urban_low | 5730300 | 0.1159006098 |
| 2013 | 11.13 | 4 | 23 | urban_med | 1465200 | 0.0296350232 |
| 2013 | 11.13 | 4 | 24 | urban_high | 512100 | 0.0103576955 |
| 2013 | 11.13 | 4 | 31 | barren | 275400 | 0.0055702194 |
| 2013 | 11.13 | 4 | 41 | dec_forest | 5576400 | 0.1127878402 |
| 2013 | 11.13 | 4 | 42 | eg_forest | 24300 | 0.0004914899 |
| 2013 | 11.13 | 4 | 43 | mix_forest | 94500 | 0.0019113498 |
| 2013 | 11.13 | 4 | 71 | grass_herb | 318600 | 0.0064439792 |
| 2013 | 11.13 | 4 | 81 | pasture_hay | 4215600 | 0.0852644034 |
| 2013 | 11.13 | 4 | 82 | crop | 25002900 | 0.5057067443 |
| 2013 | 11.13 | 4 | 90 | woody_wetland | 9000 | 0.0001820333 |
| 2013 | 11.13 | 4 | 95 | em_herb_wetland | 9900 | 0.0002002366 |
| 2013 | 14.13 | 4 | 11 | open_water | 1184400 | 0.0239603816 |
| 2013 | 14.13 | 4 | 21 | urban_open | 3701700 | 0.074885296 |
| 2013 | 14.13 | 4 | 22 | urban_low | 314100 | 0.0063542349 |
| 2013 | 14.13 | 4 | 23 | urban_med | 5400 | 0.0001092419 |
| 2013 | 14.13 | 4 | 41 | dec_forest | 35433000 | 0.7168086811 |
| 2013 | 14.13 | 4 | 42 | eg_forest | 16200 | 0.0003277256 |
| 2013 | 14.13 | 4 | 52 | scrub | 176400 | 0.0035685675 |
| 2013 | 14.13 | 4 | 71 | grass_herb | 1013400 | 0.020501056 |
| 2013 | 14.13 | 4 | 81 | pasture_hay | 5886900 | 0.119091836 |
| 2013 | 14.13 | 4 | 82 | crop | 1693800 | 0.0342655306 |
| 2013 | 14.13 | 4 | 90 | woody_wetland | 6300 | 0.0001274488 |
| 2013 | 16.13 | 4 | 11 | open_water | 390600 | 0.0079013964 |
| 2013 | 16.13 | 4 | 21 | urban_open | 10038600 | 0.2030695286 |
| 2013 | 16.13 | 4 | 22 | urban_low | 6895800 | 0.1394942378 |
| 2013 | 16.13 | 4 | 23 | urban_med | 1099800 | 0.0222477106 |
| 2013 | 16.13 | 4 | 24 | urban_high | 89100 | 0.0018023923 |
| 2013 | 16.13 | 4 | 31 | barren | 123300 | 0.0024942196 |
| 2013 | 16.13 | 4 | 41 | dec_forest | 16880400 | 0.3414714075 |
| 2013 | 16.13 | 4 | 42 | eg_forest | 539100 | 0.0109053835 |
| 2013 | 16.13 | 4 | 43 | mix_forest | 10800 | 0.0002184718 |
| 2013 | 16.13 | 4 | 52 | scrub | 75600 | 0.0015293025 |
| 2013 | 16.13 | 4 | 71 | grass_herb | 526500 | 0.0106504998 |
| 2013 | 16.13 | 4 | 81 | pasture_hay | 6012000 | 0.121615963 |
| 2013 | 16.13 | 4 | 82 | crop | 4844700 | 0.0980028037 |
| 2013 | 16.13 | 4 | 90 | woody_wetland | 1908000 | 0.0385966829 |
| 2013 | 22.13 | 4 | 11 | open_water | 230400 | 0.004661071 |
| 2013 | 22.13 | 4 | 21 | urban_open | 12666600 | 0.2562496586 |
| 2013 | 22.13 | 4 | 22 | urban_low | 13716000 | 0.2774793802 |
| 2013 | 22.13 | 4 | 23 | urban_med | 4270500 | 0.0863936784 |
| 2013 | 22.13 | 4 | 24 | urban_high | 2180700 | 0.0441163083 |
| 2013 | 22.13 | 4 | 31 | barren | 20700 | 0.0004187681 |
| 2013 | 22.13 | 4 | 41 | dec_forest | 12824100 | 0.2594359376 |
| 2013 | 22.13 | 4 | 42 | eg_forest | 334800 | 0.0067731187 |
| 2013 | 22.13 | 4 | 43 | mix_forest | 5400 | 0.0001092439 |
| 2013 | 22.13 | 4 | 52 | scrub | 7200 | 0.0001456585 |
| 2013 | 22.13 | 4 | 71 | grass_herb | 873000 | 0.0176610892 |
| 2013 | 22.13 | 4 | 81 | pasture_hay | 1252800 | 0.0253445733 |
| 2013 | 22.13 | 4 | 82 | crop | 52200 | 0.0010560239 |
| 2013 | 22.13 | 4 | 90 | woody_wetland | 925200 | 0.018717113 |
| 2013 | 22.13 | 4 | 95 | em_herb_wetland | 71100 | 0.0014383774 |
| 2013 | 23.13 | 4 | 11 | open_water | 126000 | 0.0025487911 |
| 2013 | 23.13 | 4 | 21 | urban_open | 4040100 | 0.0817251675 |
| 2013 | 23.13 | 4 | 22 | urban_low | 846900 | 0.0171315176 |
| 2013 | 23.13 | 4 | 23 | urban_med | 123300 | 0.0024941742 |
| 2013 | 23.13 | 4 | 24 | urban_high | 30600 | 0.0006189921 |
| 2013 | 23.13 | 4 | 31 | barren | 51300 | 0.0010377221 |
| 2013 | 23.13 | 4 | 41 | dec_forest | 14535900 | 0.2940394699 |
| 2013 | 23.13 | 4 | 42 | eg_forest | 230400 | 0.0046606467 |
| 2013 | 23.13 | 4 | 71 | grass_herb | 642600 | 0.0129988348 |
| 2013 | 23.13 | 4 | 81 | pasture_hay | 14593500 | 0.2952046315 |
| 2013 | 23.13 | 4 | 82 | crop | 13952700 | 0.282242208 |
| 2013 | 23.13 | 4 | 90 | woody_wetland | 261900 | 0.0052978445 |
| 2013 | 26.13 | 4 | 11 | open_water | 117000 | 0.0023682437 |
| 2013 | 26.13 | 4 | 21 | urban_open | 2515500 | 0.050917239 |
| 2013 | 26.13 | 4 | 22 | urban_low | 525600 | 0.0106388793 |
| 2013 | 26.13 | 4 | 23 | urban_med | 9900 | 0.0002003898 |
| 2013 | 26.13 | 4 | 31 | barren | 4500 | 9.11E-05 |
| 2013 | 26.13 | 4 | 41 | dec_forest | 5850000 | 0.1184121837 |
| 2013 | 26.13 | 4 | 42 | eg_forest | 13500 | 0.0002732589 |
| 2013 | 26.13 | 4 | 43 | mix_forest | 144900 | 0.0029329787 |
| 2013 | 26.13 | 4 | 52 | scrub | 28800 | 0.0005829523 |
| 2013 | 26.13 | 4 | 71 | grass_herb | 94500 | 0.0019128122 |
| 2013 | 26.13 | 4 | 81 | pasture_hay | 842400 | 0.0170513545 |
| 2013 | 26.13 | 4 | 82 | crop | 39195900 | 0.7933798481 |
| 2013 | 26.13 | 4 | 90 | woody_wetland | 52200 | 0.001056601 |
| 2013 | 26.13 | 4 | 95 | em_herb_wetland | 9000 | 0.0001821726 |
| 2013 | 28.13 | 4 | 11 | open_water | 5662800 | 0.1145687285 |
| 2013 | 28.13 | 4 | 21 | urban_open | 12407400 | 0.2510242357 |
| 2013 | 28.13 | 4 | 22 | urban_low | 6825600 | 0.1380942843 |
| 2013 | 28.13 | 4 | 23 | urban_med | 650700 | 0.0131648428 |
| 2013 | 28.13 | 4 | 24 | urban_high | 160200 | 0.0032411369 |
| 2013 | 28.13 | 4 | 31 | barren | 13500 | 0.0002731295 |
| 2013 | 28.13 | 4 | 41 | dec_forest | 14812200 | 0.2996777072 |
| 2013 | 28.13 | 4 | 42 | eg_forest | 682200 | 0.013802145 |
| 2013 | 28.13 | 4 | 43 | mix_forest | 4500 | 9.10E-05 |
| 2013 | 28.13 | 4 | 52 | scrub | 45000 | 0.0009104317 |
| 2013 | 28.13 | 4 | 71 | grass_herb | 1666800 | 0.0337223912 |
| 2013 | 28.13 | 4 | 81 | pasture_hay | 4076100 | 0.0824669058 |
| 2013 | 28.13 | 4 | 82 | crop | 2045700 | 0.0413882263 |
| 2013 | 28.13 | 4 | 90 | woody_wetland | 348300 | 0.0070467416 |
| 2013 | 28.13 | 4 | 95 | em_herb_wetland | 26100 | 0.0005280504 |
| 2013 | 36.13 | 4 | 11 | open_water | 1380600 | 0.0279274687 |
| 2013 | 36.13 | 4 | 21 | urban_open | 9756900 | 0.1973674629 |
| 2013 | 36.13 | 4 | 22 | urban_low | 10009800 | 0.2024832508 |
| 2013 | 36.13 | 4 | 23 | urban_med | 4336200 | 0.0877148267 |
| 2013 | 36.13 | 4 | 24 | urban_high | 2080800 | 0.0420914652 |
| 2013 | 36.13 | 4 | 31 | barren | 55800 | 0.0011287504 |
| 2013 | 36.13 | 4 | 41 | dec_forest | 13744800 | 0.2780367026 |
| 2013 | 36.13 | 4 | 42 | eg_forest | 217800 | 0.0044057676 |
| 2013 | 36.13 | 4 | 52 | scrub | 107100 | 0.0021664725 |
| 2013 | 36.13 | 4 | 71 | grass_herb | 680400 | 0.0137634722 |
| 2013 | 36.13 | 4 | 81 | pasture_hay | 6608700 | 0.1336840955 |
| 2013 | 36.13 | 4 | 82 | crop | 438300 | 0.0088661521 |
| 2013 | 36.13 | 4 | 90 | woody_wetland | 18000 | 0.000364113 |
| 2013 | 37.13 | 4 | 11 | open_water | 726300 | 0.0146820704 |
| 2013 | 37.13 | 4 | 21 | urban_open | 4517100 | 0.0913126535 |
| 2013 | 37.13 | 4 | 22 | urban_low | 2985300 | 0.0603474939 |
| 2013 | 37.13 | 4 | 23 | urban_med | 871200 | 0.0176112071 |
| 2013 | 37.13 | 4 | 24 | urban_high | 253800 | 0.0051305376 |
| 2013 | 37.13 | 4 | 31 | barren | 430200 | 0.0086964432 |
| 2013 | 37.13 | 4 | 41 | dec_forest | 21337200 | 0.4313290276 |
| 2013 | 37.13 | 4 | 42 | eg_forest | 268200 | 0.0054216319 |
| 2013 | 37.13 | 4 | 52 | scrub | 9000 | 0.000181934 |
| 2013 | 37.13 | 4 | 71 | grass_herb | 959400 | 0.0193941599 |
| 2013 | 37.13 | 4 | 81 | pasture_hay | 7136100 | 0.1442554353 |
| 2013 | 37.13 | 4 | 82 | crop | 9273600 | 0.1874647503 |
| 2013 | 37.13 | 4 | 90 | woody_wetland | 675900 | 0.0136632402 |
| 2013 | 37.13 | 4 | 95 | em_herb_wetland | 25200 | 0.0005094151 |
| 2013 | 38.13 | 4 | 11 | open_water | 262800 | 0.0053146955 |
| 2013 | 38.13 | 4 | 21 | urban_open | 2802600 | 0.0566779513 |
| 2013 | 38.13 | 4 | 22 | urban_low | 9000 | 0.0001820101 |
| 2013 | 38.13 | 4 | 31 | barren | 58500 | 0.0011830658 |
| 2013 | 38.13 | 4 | 41 | dec_forest | 36357300 | 0.7352662808 |
| 2013 | 38.13 | 4 | 42 | eg_forest | 413100 | 0.0083542645 |
| 2013 | 38.13 | 4 | 43 | mix_forest | 5400 | 0.0001092061 |
| 2013 | 38.13 | 4 | 52 | scrub | 9900 | 0.0002002111 |
| 2013 | 38.13 | 4 | 71 | grass_herb | 616500 | 0.0124676932 |
| 2013 | 38.13 | 4 | 81 | pasture_hay | 4991400 | 0.1009428124 |
| 2013 | 38.13 | 4 | 82 | crop | 3877200 | 0.0784099596 |
| 2013 | 38.13 | 4 | 90 | woody_wetland | 30600 | 0.0006188344 |
| 2013 | 38.13 | 4 | 95 | em_herb_wetland | 13500 | 0.0002730152 |
| 2013 | 39.13 | 4 | 11 | open_water | 2738700 | 0.0553725776 |
| 2013 | 39.13 | 4 | 21 | urban_open | 3074400 | 0.062159949 |
| 2013 | 39.13 | 4 | 22 | urban_low | 1126800 | 0.0227822764 |
| 2013 | 39.13 | 4 | 23 | urban_med | 268200 | 0.0054226185 |
| 2013 | 39.13 | 4 | 24 | urban_high | 48600 | 0.0009826221 |
| 2013 | 39.13 | 4 | 41 | dec_forest | 6107400 | 0.1234828496 |
| 2013 | 39.13 | 4 | 42 | eg_forest | 62100 | 0.0012555727 |
| 2013 | 39.13 | 4 | 43 | mix_forest | 5400 | 0.0001091802 |
| 2013 | 39.13 | 4 | 52 | scrub | 235800 | 0.0047675371 |
| 2013 | 39.13 | 4 | 71 | grass_herb | 102600 | 0.0020744245 |
| 2013 | 39.13 | 4 | 81 | pasture_hay | 5220000 | 0.1055408971 |
| 2013 | 39.13 | 4 | 82 | crop | 30315600 | 0.6129378582 |
| 2013 | 39.13 | 4 | 90 | woody_wetland | 152100 | 0.0030752434 |
| 2013 | 39.13 | 4 | 95 | em_herb_wetland | 1800 | 3.64E-05 |
| 2013 | 40.13 | 4 | 11 | open_water | 90000 | 0.0018196706 |
| 2013 | 40.13 | 4 | 21 | urban_open | 8064000 | 0.1630424893 |
| 2013 | 40.13 | 4 | 22 | urban_low | 9779400 | 0.1977254117 |
| 2013 | 40.13 | 4 | 23 | urban_med | 2675700 | 0.0540988081 |
| 2013 | 40.13 | 4 | 24 | urban_high | 895500 | 0.0181057229 |
| 2013 | 40.13 | 4 | 41 | dec_forest | 7386300 | 0.1493403694 |
| 2013 | 40.13 | 4 | 42 | eg_forest | 134100 | 0.0027113093 |
| 2013 | 40.13 | 4 | 43 | mix_forest | 4500 | 9.10E-05 |
| 2013 | 40.13 | 4 | 52 | scrub | 25200 | 0.0005095078 |
| 2013 | 40.13 | 4 | 71 | grass_herb | 255600 | 0.0051678646 |
| 2013 | 40.13 | 4 | 81 | pasture_hay | 7900200 | 0.1597306887 |
| 2013 | 40.13 | 4 | 82 | crop | 11909700 | 0.2407970157 |
| 2013 | 40.13 | 4 | 90 | woody_wetland | 339300 | 0.0068601583 |
| 2013 | 42.13 | 4 | 21 | urban_open | 2277900 | 0.0460785028 |
| 2013 | 42.13 | 4 | 22 | urban_low | 56700 | 0.001146956 |
| 2013 | 42.13 | 4 | 23 | urban_med | 9000 | 0.0001820565 |
| 2013 | 42.13 | 4 | 31 | barren | 6300 | 0.0001274396 |
| 2013 | 42.13 | 4 | 41 | dec_forest | 35766900 | 0.7235107777 |
| 2013 | 42.13 | 4 | 42 | eg_forest | 1242900 | 0.0251420041 |
| 2013 | 42.13 | 4 | 52 | scrub | 433800 | 0.0087751238 |
| 2013 | 42.13 | 4 | 71 | grass_herb | 361800 | 0.0073186717 |
| 2013 | 42.13 | 4 | 81 | pasture_hay | 9184500 | 0.1857886688 |
| 2013 | 42.13 | 4 | 82 | crop | 95400 | 0.001929799 |
| 2013 | 43.13 | 4 | 11 | open_water | 5061600 | 0.1024519984 |
| 2013 | 43.13 | 4 | 21 | urban_open | 7769700 | 0.1572667322 |
| 2013 | 43.13 | 4 | 22 | urban_low | 8081100 | 0.163569789 |
| 2013 | 43.13 | 4 | 23 | urban_med | 1657800 | 0.0335555798 |
| 2013 | 43.13 | 4 | 24 | urban_high | 180900 | 0.0036616024 |
| 2013 | 43.13 | 4 | 31 | barren | 7200 | 0.0001457354 |
| 2013 | 43.13 | 4 | 41 | dec_forest | 12324600 | 0.2494626006 |
| 2013 | 43.13 | 4 | 42 | eg_forest | 243000 | 0.0049185703 |
| 2013 | 43.13 | 4 | 52 | scrub | 416700 | 0.0084344373 |
| 2013 | 43.13 | 4 | 71 | grass_herb | 275400 | 0.0055743797 |
| 2013 | 43.13 | 4 | 81 | pasture_hay | 4380300 | 0.0886617845 |
| 2013 | 43.13 | 4 | 82 | crop | 8959500 | 0.18134951 |
| 2013 | 43.13 | 4 | 90 | woody_wetland | 36000 | 0.0007286771 |
| 2013 | 43.13 | 4 | 95 | em_herb_wetland | 10800 | 0.0002186031 |
| 2013 | 46.13 | 4 | 11 | open_water | 2169900 | 0.0438842373 |
| 2013 | 46.13 | 4 | 21 | urban_open | 11804400 | 0.2387331635 |
| 2013 | 46.13 | 4 | 22 | urban_low | 6009300 | 0.121532581 |
| 2013 | 46.13 | 4 | 23 | urban_med | 667800 | 0.0135056425 |
| 2013 | 46.13 | 4 | 24 | urban_high | 335700 | 0.0067892246 |
| 2013 | 46.13 | 4 | 31 | barren | 91800 | 0.0018565708 |
| 2013 | 46.13 | 4 | 41 | dec_forest | 10980000 | 0.2220604296 |
| 2013 | 46.13 | 4 | 42 | eg_forest | 300600 | 0.0060793593 |
| 2013 | 46.13 | 4 | 43 | mix_forest | 23400 | 0.0004732435 |
| 2013 | 46.13 | 4 | 52 | scrub | 30600 | 0.0006188569 |
| 2013 | 46.13 | 4 | 71 | grass_herb | 1121400 | 0.0226792865 |
| 2013 | 46.13 | 4 | 81 | pasture_hay | 8619300 | 0.1743174372 |
| 2013 | 46.13 | 4 | 82 | crop | 6131700 | 0.1240080087 |
| 2013 | 46.13 | 4 | 90 | woody_wetland | 1121400 | 0.0226792865 |
| 2013 | 46.13 | 4 | 95 | em_herb_wetland | 38700 | 0.000782672 |
| 2013 | 47.13 | 4 | 11 | open_water | 204300 | 0.0041315545 |
| 2013 | 47.13 | 4 | 21 | urban_open | 3871800 | 0.0782993284 |
| 2013 | 47.13 | 4 | 22 | urban_low | 772200 | 0.015616184 |
| 2013 | 47.13 | 4 | 23 | urban_med | 114300 | 0.0023114864 |
| 2013 | 47.13 | 4 | 24 | urban_high | 22500 | 0.000455017 |
| 2013 | 47.13 | 4 | 41 | dec_forest | 11614500 | 0.2348797845 |
| 2013 | 47.13 | 4 | 42 | eg_forest | 412200 | 0.0083359118 |
| 2013 | 47.13 | 4 | 43 | mix_forest | 9900 | 0.0002002075 |
| 2013 | 47.13 | 4 | 52 | scrub | 5400 | 0.0001092041 |
| 2013 | 47.13 | 4 | 71 | grass_herb | 904500 | 0.0182916841 |
| 2013 | 47.13 | 4 | 81 | pasture_hay | 12872700 | 0.2603243361 |
| 2013 | 47.13 | 4 | 82 | crop | 18587700 | 0.3758986586 |
| 2013 | 47.13 | 4 | 90 | woody_wetland | 49500 | 0.0010010374 |
| 2013 | 47.13 | 4 | 95 | em_herb_wetland | 7200 | 0.0001456054 |
| 2013 | 3.13 | 5 | 11 | open_water | 519300 | 0.0067204771 |
| 2013 | 3.13 | 5 | 21 | urban_open | 18153000 | 0.2349255157 |
| 2013 | 3.13 | 5 | 22 | urban_low | 26034300 | 0.3369206937 |
| 2013 | 3.13 | 5 | 23 | urban_med | 9421200 | 0.1219236638 |
| 2013 | 3.13 | 5 | 24 | urban_high | 2675700 | 0.0346273455 |
| 2013 | 3.13 | 5 | 31 | barren | 104400 | 0.0013510838 |
| 2013 | 3.13 | 5 | 41 | dec_forest | 16154100 | 0.2090569202 |
| 2013 | 3.13 | 5 | 42 | eg_forest | 241200 | 0.0031214694 |
| 2013 | 3.13 | 5 | 43 | mix_forest | 12600 | 0.0001630618 |
| 2013 | 3.13 | 5 | 52 | scrub | 27900 | 0.0003610655 |
| 2013 | 3.13 | 5 | 71 | grass_herb | 1588500 | 0.0205574385 |
| 2013 | 3.13 | 5 | 81 | pasture_hay | 1215900 | 0.0157354671 |
| 2013 | 3.13 | 5 | 82 | crop | 52200 | 0.0006755419 |
| 2013 | 3.13 | 5 | 90 | woody_wetland | 929700 | 0.012031634 |
| 2013 | 3.13 | 5 | 95 | em_herb_wetland | 141300 | 0.001828622 |
| 2013 | 7.13 | 5 | 11 | open_water | 728100 | 0.0094223154 |
| 2013 | 7.13 | 5 | 21 | urban_open | 7038000 | 0.0910784999 |
| 2013 | 7.13 | 5 | 22 | urban_low | 3118500 | 0.0403563941 |
| 2013 | 7.13 | 5 | 23 | urban_med | 611100 | 0.0079082227 |
| 2013 | 7.13 | 5 | 24 | urban_high | 45000 | 0.0005823433 |
| 2013 | 7.13 | 5 | 31 | barren | 47700 | 0.000617284 |
| 2013 | 7.13 | 5 | 41 | dec_forest | 54332100 | 0.7031097135 |
| 2013 | 7.13 | 5 | 42 | eg_forest | 717300 | 0.009282553 |
| 2013 | 7.13 | 5 | 43 | mix_forest | 92700 | 0.0011996273 |
| 2013 | 7.13 | 5 | 52 | scrub | 684000 | 0.0088516189 |
| 2013 | 7.13 | 5 | 71 | grass_herb | 1211400 | 0.015676683 |
| 2013 | 7.13 | 5 | 81 | pasture_hay | 6876900 | 0.0889937107 |
| 2013 | 7.13 | 5 | 82 | crop | 1771200 | 0.0229210342 |
| 2013 | 11.13 | 5 | 11 | open_water | 2927700 | 0.0379080093 |
| 2013 | 11.13 | 5 | 21 | urban_open | 8089200 | 0.1047393752 |
| 2013 | 11.13 | 5 | 22 | urban_low | 8982000 | 0.1162993952 |
| 2013 | 11.13 | 5 | 23 | urban_med | 2475900 | 0.0320580798 |
| 2013 | 11.13 | 5 | 24 | urban_high | 899100 | 0.0116415928 |
| 2013 | 11.13 | 5 | 31 | barren | 1116000 | 0.0144500251 |
| 2013 | 11.13 | 5 | 41 | dec_forest | 7108200 | 0.092037337 |
| 2013 | 11.13 | 5 | 42 | eg_forest | 45000 | 0.0005826623 |
| 2013 | 11.13 | 5 | 43 | mix_forest | 142200 | 0.0018412129 |
| 2013 | 11.13 | 5 | 71 | grass_herb | 406800 | 0.0052672672 |
| 2013 | 11.13 | 5 | 81 | pasture_hay | 5572800 | 0.0721568993 |
| 2013 | 11.13 | 5 | 82 | crop | 39361500 | 0.5096547143 |
| 2013 | 11.13 | 5 | 90 | woody_wetland | 89100 | 0.0011536714 |
| 2013 | 11.13 | 5 | 95 | em_herb_wetland | 16200 | 0.0002097584 |
| 2013 | 14.13 | 5 | 11 | open_water | 1420200 | 0.0183903224 |
| 2013 | 14.13 | 5 | 21 | urban_open | 5096700 | 0.0659977158 |
| 2013 | 14.13 | 5 | 22 | urban_low | 406800 | 0.0052676969 |
| 2013 | 14.13 | 5 | 23 | urban_med | 5400 | 6.99E-05 |
| 2013 | 14.13 | 5 | 41 | dec_forest | 56566800 | 0.7324895695 |
| 2013 | 14.13 | 5 | 42 | eg_forest | 63000 | 0.0008157938 |
| 2013 | 14.13 | 5 | 52 | scrub | 307800 | 0.0039857353 |
| 2013 | 14.13 | 5 | 71 | grass_herb | 1726200 | 0.0223527492 |
| 2013 | 14.13 | 5 | 81 | pasture_hay | 9836100 | 0.1273687155 |
| 2013 | 14.13 | 5 | 82 | crop | 1790100 | 0.0231801972 |
| 2013 | 14.13 | 5 | 90 | woody_wetland | 6300 | 8.16E-05 |
| 2013 | 16.13 | 5 | 11 | open_water | 600300 | 0.0077699082 |
| 2013 | 16.13 | 5 | 21 | urban_open | 15284700 | 0.1978356088 |
| 2013 | 16.13 | 5 | 22 | urban_low | 11670300 | 0.151053073 |
| 2013 | 16.13 | 5 | 23 | urban_med | 2139300 | 0.0276897628 |
| 2013 | 16.13 | 5 | 24 | urban_high | 265500 | 0.0034364661 |
| 2013 | 16.13 | 5 | 31 | barren | 252000 | 0.0032617306 |
| 2013 | 16.13 | 5 | 41 | dec_forest | 23984100 | 0.3104352081 |
| 2013 | 16.13 | 5 | 42 | eg_forest | 790200 | 0.0102278552 |
| 2013 | 16.13 | 5 | 43 | mix_forest | 10800 | 0.0001397885 |
| 2013 | 16.13 | 5 | 52 | scrub | 115200 | 0.0014910768 |
| 2013 | 16.13 | 5 | 71 | grass_herb | 702900 | 0.0090978985 |
| 2013 | 16.13 | 5 | 81 | pasture_hay | 9292500 | 0.1202763152 |
| 2013 | 16.13 | 5 | 82 | crop | 9016200 | 0.1167000606 |
| 2013 | 16.13 | 5 | 90 | woody_wetland | 3135600 | 0.0405852477 |
| 2013 | 22.13 | 5 | 11 | open_water | 559800 | 0.0072478123 |
| 2013 | 22.13 | 5 | 21 | urban_open | 20942100 | 0.2711404234 |
| 2013 | 22.13 | 5 | 22 | urban_low | 20495700 | 0.2653608175 |
| 2013 | 22.13 | 5 | 23 | urban_med | 6358500 | 0.082324427 |
| 2013 | 22.13 | 5 | 24 | urban_high | 2608200 | 0.0337687458 |
| 2013 | 22.13 | 5 | 31 | barren | 125100 | 0.0016196879 |
| 2013 | 22.13 | 5 | 41 | dec_forest | 20161800 | 0.2610377655 |
| 2013 | 22.13 | 5 | 42 | eg_forest | 523800 | 0.006781715 |
| 2013 | 22.13 | 5 | 43 | mix_forest | 5400 | 6.99E-05 |
| 2013 | 22.13 | 5 | 52 | scrub | 26100 | 0.0003379205 |
| 2013 | 22.13 | 5 | 71 | grass_herb | 1600200 | 0.0207180228 |
| 2013 | 22.13 | 5 | 81 | pasture_hay | 2141100 | 0.027721134 |
| 2013 | 22.13 | 5 | 82 | crop | 52200 | 0.000675841 |
| 2013 | 22.13 | 5 | 90 | woody_wetland | 1558800 | 0.020182011 |
| 2013 | 22.13 | 5 | 95 | em_herb_wetland | 78300 | 0.0010137615 |
| 2013 | 23.13 | 5 | 11 | open_water | 241200 | 0.0031231063 |
| 2013 | 23.13 | 5 | 21 | urban_open | 6179400 | 0.0800121195 |
| 2013 | 23.13 | 5 | 22 | urban_low | 1473300 | 0.019076586 |
| 2013 | 23.13 | 5 | 23 | urban_med | 251100 | 0.0032512935 |
| 2013 | 23.13 | 5 | 24 | urban_high | 129600 | 0.001678087 |
| 2013 | 23.13 | 5 | 31 | barren | 534600 | 0.0069221088 |
| 2013 | 23.13 | 5 | 41 | dec_forest | 23720400 | 0.307136531 |
| 2013 | 23.13 | 5 | 42 | eg_forest | 504900 | 0.0065375472 |
| 2013 | 23.13 | 5 | 71 | grass_herb | 1027800 | 0.013308162 |
| 2013 | 23.13 | 5 | 81 | pasture_hay | 21325500 | 0.276126882 |
| 2013 | 23.13 | 5 | 82 | crop | 21531600 | 0.2787955065 |
| 2013 | 23.13 | 5 | 90 | woody_wetland | 311400 | 0.0040320701 |
| 2013 | 26.13 | 5 | 11 | open_water | 144900 | 0.0018751893 |
| 2013 | 26.13 | 5 | 21 | urban_open | 4479300 | 0.0579678073 |
| 2013 | 26.13 | 5 | 22 | urban_low | 850500 | 0.0110065457 |
| 2013 | 26.13 | 5 | 23 | urban_med | 22500 | 0.0002911785 |
| 2013 | 26.13 | 5 | 31 | barren | 4500 | 5.82E-05 |
| 2013 | 26.13 | 5 | 41 | dec_forest | 8576100 | 0.1109855808 |
| 2013 | 26.13 | 5 | 42 | eg_forest | 13500 | 0.0001747071 |
| 2013 | 26.13 | 5 | 43 | mix_forest | 151200 | 0.0019567192 |
| 2013 | 26.13 | 5 | 52 | scrub | 86400 | 0.0011181253 |
| 2013 | 26.13 | 5 | 71 | grass_herb | 127800 | 0.0016538936 |
| 2013 | 26.13 | 5 | 81 | pasture_hay | 1881000 | 0.024342519 |
| 2013 | 26.13 | 5 | 82 | crop | 60723000 | 0.785832421 |
| 2013 | 26.13 | 5 | 90 | woody_wetland | 202500 | 0.0026206061 |
| 2013 | 26.13 | 5 | 95 | em_herb_wetland | 9000 | 0.0001164714 |
| 2013 | 28.13 | 5 | 11 | open_water | 7002900 | 0.0906707374 |
| 2013 | 28.13 | 5 | 21 | urban_open | 18667800 | 0.2417031789 |
| 2013 | 28.13 | 5 | 22 | urban_low | 9992700 | 0.1293814673 |
| 2013 | 28.13 | 5 | 23 | urban_med | 1077300 | 0.0139484478 |
| 2013 | 28.13 | 5 | 24 | urban_high | 243000 | 0.0031462664 |
| 2013 | 28.13 | 5 | 31 | barren | 806400 | 0.0104409434 |
| 2013 | 28.13 | 5 | 41 | dec_forest | 23301000 | 0.3016919922 |
| 2013 | 28.13 | 5 | 42 | eg_forest | 932400 | 0.0120723408 |
| 2013 | 28.13 | 5 | 43 | mix_forest | 4500 | 5.83E-05 |
| 2013 | 28.13 | 5 | 52 | scrub | 45000 | 0.0005826419 |
| 2013 | 28.13 | 5 | 71 | grass_herb | 2827800 | 0.036613219 |
| 2013 | 28.13 | 5 | 81 | pasture_hay | 8574300 | 0.1110165936 |
| 2013 | 28.13 | 5 | 82 | crop | 3116700 | 0.0403537802 |
| 2013 | 28.13 | 5 | 90 | woody_wetland | 608400 | 0.0078773189 |
| 2013 | 28.13 | 5 | 95 | em_herb_wetland | 34200 | 0.0004428079 |
| 2013 | 36.13 | 5 | 11 | open_water | 1767600 | 0.0228856417 |
| 2013 | 36.13 | 5 | 21 | urban_open | 15039900 | 0.1947260481 |
| 2013 | 36.13 | 5 | 22 | urban_low | 12427200 | 0.160898646 |
| 2013 | 36.13 | 5 | 23 | urban_med | 5018400 | 0.0649747139 |
| 2013 | 36.13 | 5 | 24 | urban_high | 2411100 | 0.0312172272 |
| 2013 | 36.13 | 5 | 31 | barren | 64800 | 0.0008389848 |
| 2013 | 36.13 | 5 | 41 | dec_forest | 25137000 | 0.325456198 |
| 2013 | 36.13 | 5 | 42 | eg_forest | 362700 | 0.0046959845 |
| 2013 | 36.13 | 5 | 52 | scrub | 118800 | 0.0015381389 |
| 2013 | 36.13 | 5 | 71 | grass_herb | 864000 | 0.0111864644 |
| 2013 | 36.13 | 5 | 81 | pasture_hay | 11205000 | 0.1450744599 |
| 2013 | 36.13 | 5 | 82 | crop | 2801700 | 0.0362744413 |
| 2013 | 36.13 | 5 | 90 | woody_wetland | 18000 | 0.0002330513 |
| 2013 | 37.13 | 5 | 11 | open_water | 909900 | 0.0117804708 |
| 2013 | 37.13 | 5 | 21 | urban_open | 6278400 | 0.0812864134 |
| 2013 | 37.13 | 5 | 22 | urban_low | 3586500 | 0.0464343976 |
| 2013 | 37.13 | 5 | 23 | urban_med | 1082700 | 0.0140177115 |
| 2013 | 37.13 | 5 | 24 | urban_high | 337500 | 0.0043696108 |
| 2013 | 37.13 | 5 | 31 | barren | 842400 | 0.0109065486 |
| 2013 | 37.13 | 5 | 41 | dec_forest | 34931700 | 0.4522605453 |
| 2013 | 37.13 | 5 | 42 | eg_forest | 466200 | 0.0060358891 |
| 2013 | 37.13 | 5 | 52 | scrub | 26100 | 0.0003379166 |
| 2013 | 37.13 | 5 | 71 | grass_herb | 1617300 | 0.020939175 |
| 2013 | 37.13 | 5 | 81 | pasture_hay | 12231000 | 0.1583546959 |
| 2013 | 37.13 | 5 | 82 | crop | 13900500 | 0.179969704 |
| 2013 | 37.13 | 5 | 90 | woody_wetland | 990000 | 0.0128175251 |
| 2013 | 37.13 | 5 | 95 | em_herb_wetland | 37800 | 0.0004893964 |
| 2013 | 38.13 | 5 | 11 | open_water | 1420200 | 0.0183903224 |
| 2013 | 38.13 | 5 | 21 | urban_open | 5096700 | 0.0659977158 |
| 2013 | 38.13 | 5 | 22 | urban_low | 406800 | 0.0052676969 |
| 2013 | 38.13 | 5 | 23 | urban_med | 5400 | 6.99E-05 |
| 2013 | 38.13 | 5 | 41 | dec_forest | 56566800 | 0.7324895695 |
| 2013 | 38.13 | 5 | 42 | eg_forest | 63000 | 0.0008157938 |
| 2013 | 38.13 | 5 | 52 | scrub | 307800 | 0.0039857353 |
| 2013 | 38.13 | 5 | 71 | grass_herb | 1726200 | 0.0223527492 |
| 2013 | 38.13 | 5 | 81 | pasture_hay | 9836100 | 0.1273687155 |
| 2013 | 38.13 | 5 | 82 | crop | 1790100 | 0.0231801972 |
| 2013 | 38.13 | 5 | 90 | woody_wetland | 6300 | 8.16E-05 |
| 2013 | 39.13 | 5 | 11 | open_water | 5682600 | 0.0736094757 |
| 2013 | 39.13 | 5 | 21 | urban_open | 5374800 | 0.0696223929 |
| 2013 | 39.13 | 5 | 22 | urban_low | 2049300 | 0.0265455775 |
| 2013 | 39.13 | 5 | 23 | urban_med | 393300 | 0.0050946058 |
| 2013 | 39.13 | 5 | 24 | urban_high | 56700 | 0.0007344626 |
| 2013 | 39.13 | 5 | 41 | dec_forest | 8276400 | 0.107208226 |
| 2013 | 39.13 | 5 | 42 | eg_forest | 83700 | 0.0010842067 |
| 2013 | 39.13 | 5 | 43 | mix_forest | 5400 | 6.99E-05 |
| 2013 | 39.13 | 5 | 52 | scrub | 339300 | 0.0043951176 |
| 2013 | 39.13 | 5 | 71 | grass_herb | 170100 | 0.0022033879 |
| 2013 | 39.13 | 5 | 81 | pasture_hay | 7747200 | 0.1003532415 |
| 2013 | 39.13 | 5 | 82 | crop | 46748700 | 0.6055585996 |
| 2013 | 39.13 | 5 | 90 | woody_wetland | 254700 | 0.0032992527 |
| 2013 | 39.13 | 5 | 95 | em_herb_wetland | 17100 | 0.0002215046 |
| 2013 | 40.13 | 5 | 11 | open_water | 164700 | 0.0021325937 |
| 2013 | 40.13 | 5 | 21 | urban_open | 12496500 | 0.1618090921 |
| 2013 | 40.13 | 5 | 22 | urban_low | 12384000 | 0.1603524024 |
| 2013 | 40.13 | 5 | 23 | urban_med | 2965500 | 0.0383983405 |
| 2013 | 40.13 | 5 | 24 | urban_high | 1029600 | 0.0133316242 |
| 2013 | 40.13 | 5 | 31 | barren | 53100 | 0.0006875575 |
| 2013 | 40.13 | 5 | 41 | dec_forest | 12870000 | 0.1666453019 |
| 2013 | 40.13 | 5 | 42 | eg_forest | 178200 | 0.0023073965 |
| 2013 | 40.13 | 5 | 43 | mix_forest | 4500 | 5.83E-05 |
| 2013 | 40.13 | 5 | 52 | scrub | 25200 | 0.0003262985 |
| 2013 | 40.13 | 5 | 71 | grass_herb | 807300 | 0.0104532053 |
| 2013 | 40.13 | 5 | 81 | pasture_hay | 15417900 | 0.1996364103 |
| 2013 | 40.13 | 5 | 82 | crop | 18347400 | 0.2375686101 |
| 2013 | 40.13 | 5 | 90 | woody_wetland | 486000 | 0.0062928995 |
| 2013 | 42.13 | 5 | 21 | urban_open | 3606300 | 0.046666822 |
| 2013 | 42.13 | 5 | 22 | urban_low | 63900 | 0.000826889 |
| 2013 | 42.13 | 5 | 23 | urban_med | 9000 | 0.0001164632 |
| 2013 | 42.13 | 5 | 31 | barren | 6300 | 8.15E-05 |
| 2013 | 42.13 | 5 | 41 | dec_forest | 55089900 | 0.7128831641 |
| 2013 | 42.13 | 5 | 42 | eg_forest | 2478600 | 0.0320739775 |
| 2013 | 42.13 | 5 | 52 | scrub | 748800 | 0.0096897419 |
| 2013 | 42.13 | 5 | 71 | grass_herb | 505800 | 0.0065452343 |
| 2013 | 42.13 | 5 | 81 | pasture_hay | 14345100 | 0.1856307649 |
| 2013 | 42.13 | 5 | 82 | crop | 423900 | 0.0054854188 |
| 2013 | 43.13 | 5 | 11 | open_water | 5998500 | 0.0776254644 |
| 2013 | 43.13 | 5 | 21 | urban_open | 13524300 | 0.1750154319 |
| 2013 | 43.13 | 5 | 22 | urban_low | 13675500 | 0.1769720828 |
| 2013 | 43.13 | 5 | 23 | urban_med | 3768300 | 0.0487648641 |
| 2013 | 43.13 | 5 | 24 | urban_high | 761400 | 0.0098531347 |
| 2013 | 43.13 | 5 | 31 | barren | 7200 | 9.32E-05 |
| 2013 | 43.13 | 5 | 41 | dec_forest | 17391600 | 0.2250614365 |
| 2013 | 43.13 | 5 | 42 | eg_forest | 261000 | 0.0033775521 |
| 2013 | 43.13 | 5 | 52 | scrub | 496800 | 0.0064289957 |
| 2013 | 43.13 | 5 | 71 | grass_herb | 544500 | 0.0070462725 |
| 2013 | 43.13 | 5 | 81 | pasture_hay | 6520500 | 0.0843805686 |
| 2013 | 43.13 | 5 | 82 | crop | 14265000 | 0.1846006918 |
| 2013 | 43.13 | 5 | 90 | woody_wetland | 44100 | 0.0005706898 |
| 2013 | 43.13 | 5 | 95 | em_herb_wetland | 16200 | 0.0002096412 |
| 2013 | 46.13 | 5 | 11 | open_water | 2844900 | 0.0368470747 |
| 2013 | 46.13 | 5 | 21 | urban_open | 18867600 | 0.2443726905 |
| 2013 | 46.13 | 5 | 22 | urban_low | 10486800 | 0.1358247753 |
| 2013 | 46.13 | 5 | 23 | urban_med | 1385100 | 0.0179397811 |
| 2013 | 46.13 | 5 | 24 | urban_high | 781200 | 0.0101180832 |
| 2013 | 46.13 | 5 | 31 | barren | 160200 | 0.0020749065 |
| 2013 | 46.13 | 5 | 41 | dec_forest | 16821900 | 0.2178768345 |
| 2013 | 46.13 | 5 | 42 | eg_forest | 409500 | 0.0053038339 |
| 2013 | 46.13 | 5 | 43 | mix_forest | 30600 | 0.0003963304 |
| 2013 | 46.13 | 5 | 52 | scrub | 30600 | 0.0003963304 |
| 2013 | 46.13 | 5 | 71 | grass_herb | 1823400 | 0.0236166319 |
| 2013 | 46.13 | 5 | 81 | pasture_hay | 12607200 | 0.1632881439 |
| 2013 | 46.13 | 5 | 82 | crop | 9360000 | 0.1212304895 |
| 2013 | 46.13 | 5 | 90 | woody_wetland | 1554300 | 0.0201312553 |
| 2013 | 46.13 | 5 | 95 | em_herb_wetland | 45000 | 0.0005828389 |
| 2013 | 47.13 | 5 | 11 | open_water | 241200 | 0.0031243442 |
| 2013 | 47.13 | 5 | 21 | urban_open | 5299200 | 0.0686423092 |
| 2013 | 47.13 | 5 | 22 | urban_low | 1074600 | 0.0139196531 |
| 2013 | 47.13 | 5 | 23 | urban_med | 118800 | 0.0015388561 |
| 2013 | 47.13 | 5 | 24 | urban_high | 22500 | 0.00029145 |
| 2013 | 47.13 | 5 | 31 | barren | 75600 | 0.0009792721 |
| 2013 | 47.13 | 5 | 41 | dec_forest | 16677000 | 0.2160227564 |
| 2013 | 47.13 | 5 | 42 | eg_forest | 500400 | 0.0064818485 |
| 2013 | 47.13 | 5 | 43 | mix_forest | 18900 | 0.000244818 |
| 2013 | 47.13 | 5 | 52 | scrub | 10800 | 0.000139896 |
| 2013 | 47.13 | 5 | 71 | grass_herb | 1139400 | 0.0147590291 |
| 2013 | 47.13 | 5 | 81 | pasture_hay | 19606500 | 0.2539695493 |
| 2013 | 47.13 | 5 | 82 | crop | 32241600 | 0.4176362237 |
| 2013 | 47.13 | 5 | 90 | woody_wetland | 162900 | 0.0021100982 |
| 2013 | 47.13 | 5 | 95 | em_herb_wetland | 10800 | 0.000139896 |
