# Supplementary material for: Honey bee success predicted by landscape composition in Ohio, USA
Source: PeerJ. 2015 Mar 19;3:e838. doi: 10.7717/peerj.838 (PMC4369331; doi:10.7717/peerj.838)
Supplement: Table S3 — This spreadsheet shows Simpson’s Diversity Index (DIVSI) calculated for each study site. [file peerj-03-838-s003.doc]

| year | id | scale | variable | value |
| --- | --- | --- | --- | --- |
| 2012 | 9.12 | 0.5 | DIV_SI | 0.7918743325 |
| 2012 | 11.12 | 0.5 | DIV_SI | 0.5740755162 |
| 2012 | 12.12 | 0.5 | DIV_SI | 0.5028283323 |
| 2012 | 17.12 | 0.5 | DIV_SI | 0.5139582425 |
| 2012 | 20.12 | 0.5 | DIV_SI | 0.5304265671 |
| 2012 | 21.12 | 0.5 | DIV_SI | 0.5893875924 |
| 2012 | 26.12 | 0.5 | DIV_SI | 0.703283622 |
| 2012 | 27.12 | 0.5 | DIV_SI | 0.5327120909 |
| 2012 | 28.12 | 0.5 | DIV_SI | 0.7663439697 |
| 2012 | 30.12 | 0.5 | DIV_SI | 0.6218461969 |
| 2012 | 33.12 | 0.5 | DIV_SI | 0.5569954649 |
| 2012 | 36.12 | 0.5 | DIV_SI | 0.6524622837 |
| 2012 | 40.12 | 0.5 | DIV_SI | 0.552860194 |
| 2012 | 41.12 | 0.5 | DIV_SI | 0.6464474938 |
| 2012 | 42.12 | 0.5 | DIV_SI | 0.2761870803 |
| 2012 | 43.12 | 0.5 | DIV_SI | 0.6706439423 |
| 2012 | 44.12 | 0.5 | DIV_SI | 0.2885524759 |
| 2012 | 45.12 | 0.5 | DIV_SI | 0.6060173851 |
| 2012 | 47.12 | 0.5 | DIV_SI | 0.2493475066 |
| 2012 | 50.12 | 0.5 | DIV_SI | 0.5715726623 |
| 2012 | 52.12 | 0.5 | DIV_SI | 0.714236353 |
| 2012 | 53.12 | 0.5 | DIV_SI | 0.5722148674 |
| 2012 | 54.12 | 0.5 | DIV_SI | 0.569514948 |
| 2012 | 101.12 | 0.5 | DIV_SI | 0.734491812 |
| 2012 | 102.12 | 0.5 | DIV_SI | 0.5299121315 |
| 2012 | 104.12 | 0.5 | DIV_SI | 0.0748861528 |
| 2012 | 107.12 | 0.5 | DIV_SI | 0.6758994527 |
| 2012 | 108.12 | 0.5 | DIV_SI | 0.6204652879 |
| 2012 | 111.12 | 0.5 | DIV_SI | 0.6407123634 |
| 2012 | 112.12 | 0.5 | DIV_SI | 0.5327120909 |
| 2012 | 113.12 | 0.5 | DIV_SI | 0.5846636926 |
| 2012 | 115.12 | 0.5 | DIV_SI | 0.7304235476 |
| 2012 | 9.12 | 1 | DIV_SI | 0.7746463424 |
| 2012 | 11.12 | 1 | DIV_SI | 0.6044083755 |
| 2012 | 12.12 | 1 | DIV_SI | 0.6519388732 |
| 2012 | 17.12 | 1 | DIV_SI | 0.7076400448 |
| 2012 | 20.12 | 1 | DIV_SI | 0.4353987959 |
| 2012 | 21.12 | 1 | DIV_SI | 0.652592685 |
| 2012 | 26.12 | 1 | DIV_SI | 0.7165940415 |
| 2012 | 27.12 | 1 | DIV_SI | 0.3212601204 |
| 2012 | 28.12 | 1 | DIV_SI | 0.725083859 |
| 2012 | 30.12 | 1 | DIV_SI | 0.746859951 |
| 2012 | 33.12 | 1 | DIV_SI | 0.6562150937 |
| 2012 | 36.12 | 1 | DIV_SI | 0.6712731061 |
| 2012 | 40.12 | 1 | DIV_SI | 0.6206436666 |
| 2012 | 41.12 | 1 | DIV_SI | 0.6433715392 |
| 2012 | 42.12 | 1 | DIV_SI | 0.2165498786 |
| 2012 | 43.12 | 1 | DIV_SI | 0.607816329 |
| 2012 | 44.12 | 1 | DIV_SI | 0.5737178875 |
| 2012 | 45.12 | 1 | DIV_SI | 0.6842248861 |
| 2012 | 47.12 | 1 | DIV_SI | 0.6224100145 |
| 2012 | 50.12 | 1 | DIV_SI | 0.6687247947 |
| 2012 | 52.12 | 1 | DIV_SI | 0.6760502493 |
| 2012 | 53.12 | 1 | DIV_SI | 0.6415595665 |
| 2012 | 54.12 | 1 | DIV_SI | 0.6075607879 |
| 2012 | 101.12 | 1 | DIV_SI | 0.7548931454 |
| 2012 | 102.12 | 1 | DIV_SI | 0.5897798899 |
| 2012 | 104.12 | 1 | DIV_SI | 0.0878772094 |
| 2012 | 107.12 | 1 | DIV_SI | 0.5075994725 |
| 2012 | 108.12 | 1 | DIV_SI | 0.6540977022 |
| 2012 | 111.12 | 1 | DIV_SI | 0.6914412501 |
| 2012 | 112.12 | 1 | DIV_SI | 0.6294016029 |
| 2012 | 113.12 | 1 | DIV_SI | 0.5793857214 |
| 2012 | 115.12 | 1 | DIV_SI | 0.7506796136 |
| 2012 | 9.12 | 2 | DIV_SI | 0.786362061 |
| 2012 | 11.12 | 2 | DIV_SI | 0.5807453836 |
| 2012 | 12.12 | 2 | DIV_SI | 0.7138226204 |
| 2012 | 17.12 | 2 | DIV_SI | 0.7513714111 |
| 2012 | 20.12 | 2 | DIV_SI | 0.4584228878 |
| 2012 | 21.12 | 2 | DIV_SI | 0.7044966612 |
| 2012 | 26.12 | 2 | DIV_SI | 0.6676261749 |
| 2012 | 27.12 | 2 | DIV_SI | 0.4165420828 |
| 2012 | 28.12 | 2 | DIV_SI | 0.7827581751 |
| 2012 | 30.12 | 2 | DIV_SI | 0.7985626323 |
| 2012 | 33.12 | 2 | DIV_SI | 0.7685151202 |
| 2012 | 36.12 | 2 | DIV_SI | 0.7604730066 |
| 2012 | 40.12 | 2 | DIV_SI | 0.6158202181 |
| 2012 | 41.12 | 2 | DIV_SI | 0.7093920851 |
| 2012 | 42.12 | 2 | DIV_SI | 0.2745282683 |
| 2012 | 43.12 | 2 | DIV_SI | 0.4406080987 |
| 2012 | 44.12 | 2 | DIV_SI | 0.6537554849 |
| 2012 | 45.12 | 2 | DIV_SI | 0.7526663535 |
| 2012 | 47.12 | 2 | DIV_SI | 0.7138155217 |
| 2012 | 50.12 | 2 | DIV_SI | 0.6808415046 |
| 2012 | 52.12 | 2 | DIV_SI | 0.6485835321 |
| 2012 | 53.12 | 2 | DIV_SI | 0.7384084493 |
| 2012 | 54.12 | 2 | DIV_SI | 0.6264292726 |
| 2012 | 101.12 | 2 | DIV_SI | 0.789368807 |
| 2012 | 102.12 | 2 | DIV_SI | 0.6933397483 |
| 2012 | 104.12 | 2 | DIV_SI | 0.0893765158 |
| 2012 | 107.12 | 2 | DIV_SI | 0.3987447481 |
| 2012 | 108.12 | 2 | DIV_SI | 0.7020132911 |
| 2012 | 111.12 | 2 | DIV_SI | 0.682423395 |
| 2012 | 112.12 | 2 | DIV_SI | 0.7201939986 |
| 2012 | 113.12 | 2 | DIV_SI | 0.5570695917 |
| 2012 | 115.12 | 2 | DIV_SI | 0.7570794163 |
| 2012 | 9.12 | 3 | DIV_SI | 0.7895411427 |
| 2012 | 11.12 | 3 | DIV_SI | 0.6401266193 |
| 2012 | 12.12 | 3 | DIV_SI | 0.7474105143 |
| 2012 | 17.12 | 3 | DIV_SI | 0.781243399 |
| 2012 | 20.12 | 3 | DIV_SI | 0.438972842 |
| 2012 | 21.12 | 3 | DIV_SI | 0.7382347386 |
| 2012 | 26.12 | 3 | DIV_SI | 0.6493199373 |
| 2012 | 27.12 | 3 | DIV_SI | 0.505084543 |
| 2012 | 28.12 | 3 | DIV_SI | 0.7671483066 |
| 2012 | 30.12 | 3 | DIV_SI | 0.8059488193 |
| 2012 | 33.12 | 3 | DIV_SI | 0.7806459352 |
| 2012 | 36.12 | 3 | DIV_SI | 0.7751893632 |
| 2012 | 40.12 | 3 | DIV_SI | 0.581814037 |
| 2012 | 41.12 | 3 | DIV_SI | 0.7098090658 |
| 2012 | 42.12 | 3 | DIV_SI | 0.3011472231 |
| 2012 | 43.12 | 3 | DIV_SI | 0.4604265987 |
| 2012 | 44.12 | 3 | DIV_SI | 0.642362593 |
| 2012 | 45.12 | 3 | DIV_SI | 0.6256287425 |
| 2012 | 47.12 | 3 | DIV_SI | 0.7663821278 |
| 2012 | 50.12 | 3 | DIV_SI | 0.6102835505 |
| 2012 | 52.12 | 3 | DIV_SI | 0.6439094301 |
| 2012 | 53.12 | 3 | DIV_SI | 0.7463806022 |
| 2012 | 54.12 | 3 | DIV_SI | 0.6603384644 |
| 2012 | 101.12 | 3 | DIV_SI | 0.7921170503 |
| 2012 | 102.12 | 3 | DIV_SI | 0.6919775387 |
| 2012 | 104.12 | 3 | DIV_SI | 0.0980774825 |
| 2012 | 107.12 | 3 | DIV_SI | 0.35169267 |
| 2012 | 108.12 | 3 | DIV_SI | 0.7126841245 |
| 2012 | 111.12 | 3 | DIV_SI | 0.7079681066 |
| 2012 | 112.12 | 3 | DIV_SI | 0.7667758935 |
| 2012 | 113.12 | 3 | DIV_SI | 0.6349014367 |
| 2012 | 115.12 | 3 | DIV_SI | 0.7709906981 |
| 2012 | 9.12 | 4 | DIV_SI | 0.8145853518 |
| 2012 | 11.12 | 4 | DIV_SI | 0.6983720941 |
| 2012 | 12.12 | 4 | DIV_SI | 0.7495597361 |
| 2012 | 17.12 | 4 | DIV_SI | 0.7945332087 |
| 2012 | 20.12 | 4 | DIV_SI | 0.3773043391 |
| 2012 | 21.12 | 4 | DIV_SI | 0.757810015 |
| 2012 | 26.12 | 4 | DIV_SI | 0.627715023 |
| 2012 | 27.12 | 4 | DIV_SI | 0.5415294655 |
| 2012 | 28.12 | 4 | DIV_SI | 0.7633812769 |
| 2012 | 30.12 | 4 | DIV_SI | 0.809242638 |
| 2012 | 33.12 | 4 | DIV_SI | 0.7986179385 |
| 2012 | 36.12 | 4 | DIV_SI | 0.7787679605 |
| 2012 | 40.12 | 4 | DIV_SI | 0.5742746622 |
| 2012 | 41.12 | 4 | DIV_SI | 0.712329795 |
| 2012 | 42.12 | 4 | DIV_SI | 0.3183007954 |
| 2012 | 43.12 | 4 | DIV_SI | 0.4884588375 |
| 2012 | 44.12 | 4 | DIV_SI | 0.6381609088 |
| 2012 | 45.12 | 4 | DIV_SI | 0.5246614288 |
| 2012 | 47.12 | 4 | DIV_SI | 0.790864046 |
| 2012 | 50.12 | 4 | DIV_SI | 0.5804578997 |
| 2012 | 52.12 | 4 | DIV_SI | 0.6251183624 |
| 2012 | 53.12 | 4 | DIV_SI | 0.7590140337 |
| 2012 | 54.12 | 4 | DIV_SI | 0.6738463138 |
| 2012 | 101.12 | 4 | DIV_SI | 0.7981552004 |
| 2012 | 102.12 | 4 | DIV_SI | 0.710276032 |
| 2012 | 104.12 | 4 | DIV_SI | 0.1464070916 |
| 2012 | 107.12 | 4 | DIV_SI | 0.3719156956 |
| 2012 | 108.12 | 4 | DIV_SI | 0.7179582785 |
| 2012 | 111.12 | 4 | DIV_SI | 0.73063623 |
| 2012 | 112.12 | 4 | DIV_SI | 0.81397132 |
| 2012 | 113.12 | 4 | DIV_SI | 0.6866593997 |
| 2012 | 115.12 | 4 | DIV_SI | 0.7871887297 |
| 2012 | 9.12 | 5 | DIV_SI | 0.8177222727 |
| 2012 | 11.12 | 5 | DIV_SI | 0.7016790483 |
| 2012 | 12.12 | 5 | DIV_SI | 0.744678997 |
| 2012 | 17.12 | 5 | DIV_SI | 0.7992459465 |
| 2012 | 20.12 | 5 | DIV_SI | 0.3746592834 |
| 2012 | 21.12 | 5 | DIV_SI | 0.7671219488 |
| 2012 | 26.12 | 5 | DIV_SI | 0.6262431511 |
| 2012 | 27.12 | 5 | DIV_SI | 0.5657290715 |
| 2012 | 28.12 | 5 | DIV_SI | 0.7790821675 |
| 2012 | 30.12 | 5 | DIV_SI | 0.8153762369 |
| 2012 | 33.12 | 5 | DIV_SI | 0.8109250328 |
| 2012 | 36.12 | 5 | DIV_SI | 0.7707824114 |
| 2012 | 40.12 | 5 | DIV_SI | 0.5954278895 |
| 2012 | 41.12 | 5 | DIV_SI | 0.7256223169 |
| 2012 | 42.12 | 5 | DIV_SI | 0.3610154879 |
| 2012 | 43.12 | 5 | DIV_SI | 0.4987707919 |
| 2012 | 44.12 | 5 | DIV_SI | 0.6466200702 |
| 2012 | 45.12 | 5 | DIV_SI | 0.4670476357 |
| 2012 | 47.12 | 5 | DIV_SI | 0.803239828 |
| 2012 | 50.12 | 5 | DIV_SI | 0.5514840602 |
| 2012 | 52.12 | 5 | DIV_SI | 0.6226338645 |
| 2012 | 53.12 | 5 | DIV_SI | 0.7763022922 |
| 2012 | 54.12 | 5 | DIV_SI | 0.6818497415 |
| 2012 | 101.12 | 5 | DIV_SI | 0.7942797323 |
| 2012 | 102.12 | 5 | DIV_SI | 0.7264703329 |
| 2012 | 104.12 | 5 | DIV_SI | 0.1488187008 |
| 2012 | 107.12 | 5 | DIV_SI | 0.3918350348 |
| 2012 | 108.12 | 5 | DIV_SI | 0.7198614396 |
| 2012 | 111.12 | 5 | DIV_SI | 0.7588350684 |
| 2012 | 112.12 | 5 | DIV_SI | 0.808345237 |
| 2012 | 113.12 | 5 | DIV_SI | 0.6937027584 |
| 2012 | 115.12 | 5 | DIV_SI | 0.7970449325 |
| 2013 | 3.13 | 0.5 | DIV_SI | 0.5401456978 |
| 2013 | 7.13 | 0.5 | DIV_SI | 0.2032958605 |
| 2013 | 11.13 | 0.5 | DIV_SI | 0.663934563 |
| 2013 | 14.13 | 0.5 | DIV_SI | 0.7111508252 |
| 2013 | 16.13 | 0.5 | DIV_SI | 0.3654756737 |
| 2013 | 22.13 | 0.5 | DIV_SI | 0.5218634207 |
| 2013 | 23.13 | 0.5 | DIV_SI | 0.6492770876 |
| 2013 | 26.13 | 0.5 | DIV_SI | 0.266927405 |
| 2013 | 28.13 | 0.5 | DIV_SI | 0.7071310872 |
| 2013 | 36.13 | 0.5 | DIV_SI | 0.7020849955 |
| 2013 | 37.13 | 0.5 | DIV_SI | 0.712066436 |
| 2013 | 38.13 | 0.5 | DIV_SI | 0.6732882639 |
| 2013 | 39.13 | 0.5 | DIV_SI | 0.496153496 |
| 2013 | 40.13 | 0.5 | DIV_SI | 0.6957172957 |
| 2013 | 42.13 | 0.5 | DIV_SI | 0.3881886347 |
| 2013 | 43.13 | 0.5 | DIV_SI | 0.4595034902 |
| 2013 | 46.13 | 0.5 | DIV_SI | 0.7493590433 |
| 2013 | 47.13 | 0.5 | DIV_SI | 0.6851017301 |
| 2013 | 3.13 | 1 | DIV_SI | 0.6234297752 |
| 2013 | 7.13 | 1 | DIV_SI | 0.5023287621 |
| 2013 | 11.13 | 1 | DIV_SI | 0.7284040988 |
| 2013 | 14.13 | 1 | DIV_SI | 0.6534998383 |
| 2013 | 16.13 | 1 | DIV_SI | 0.6002232618 |
| 2013 | 22.13 | 1 | DIV_SI | 0.727166743 |
| 2013 | 23.13 | 1 | DIV_SI | 0.7345612489 |
| 2013 | 26.13 | 1 | DIV_SI | 0.3383234909 |
| 2013 | 28.13 | 1 | DIV_SI | 0.7810803065 |
| 2013 | 36.13 | 1 | DIV_SI | 0.7938929623 |
| 2013 | 37.13 | 1 | DIV_SI | 0.8250721024 |
| 2013 | 38.13 | 1 | DIV_SI | 0.6389814086 |
| 2013 | 39.13 | 1 | DIV_SI | 0.4596124161 |
| 2013 | 40.13 | 1 | DIV_SI | 0.7521743442 |
| 2013 | 42.13 | 1 | DIV_SI | 0.3641169645 |
| 2013 | 43.13 | 1 | DIV_SI | 0.6919850685 |
| 2013 | 46.13 | 1 | DIV_SI | 0.7969395747 |
| 2013 | 47.13 | 1 | DIV_SI | 0.7166946273 |
| 2013 | 3.13 | 2 | DIV_SI | 0.7061027294 |
| 2013 | 7.13 | 2 | DIV_SI | 0.5285102497 |
| 2013 | 11.13 | 2 | DIV_SI | 0.7216382812 |
| 2013 | 14.13 | 2 | DIV_SI | 0.5632311964 |
| 2013 | 16.13 | 2 | DIV_SI | 0.702061739 |
| 2013 | 22.13 | 2 | DIV_SI | 0.7219157248 |
| 2013 | 23.13 | 2 | DIV_SI | 0.7477068016 |
| 2013 | 26.13 | 2 | DIV_SI | 0.3644318269 |
| 2013 | 28.13 | 2 | DIV_SI | 0.7976875777 |
| 2013 | 36.13 | 2 | DIV_SI | 0.8099576463 |
| 2013 | 37.13 | 2 | DIV_SI | 0.7955063342 |
| 2013 | 38.13 | 2 | DIV_SI | 0.4598109336 |
| 2013 | 39.13 | 2 | DIV_SI | 0.5005813437 |
| 2013 | 40.13 | 2 | DIV_SI | 0.8114748222 |
| 2013 | 42.13 | 2 | DIV_SI | 0.4428227536 |
| 2013 | 43.13 | 2 | DIV_SI | 0.7814270748 |
| 2013 | 46.13 | 2 | DIV_SI | 0.8269132995 |
| 2013 | 47.13 | 2 | DIV_SI | 0.7333059355 |
| 2013 | 3.13 | 3 | DIV_SI | 0.7285472429 |
| 2013 | 7.13 | 3 | DIV_SI | 0.5735400515 |
| 2013 | 11.13 | 3 | DIV_SI | 0.7087360436 |
| 2013 | 14.13 | 3 | DIV_SI | 0.4972530791 |
| 2013 | 16.13 | 3 | DIV_SI | 0.7818147484 |
| 2013 | 22.13 | 3 | DIV_SI | 0.7648515312 |
| 2013 | 23.13 | 3 | DIV_SI | 0.7452668909 |
| 2013 | 26.13 | 3 | DIV_SI | 0.3468508632 |
| 2013 | 28.13 | 3 | DIV_SI | 0.8051320281 |
| 2013 | 36.13 | 3 | DIV_SI | 0.812089368 |
| 2013 | 37.13 | 3 | DIV_SI | 0.7651620697 |
| 2013 | 38.13 | 3 | DIV_SI | 0.4245297241 |
| 2013 | 39.13 | 3 | DIV_SI | 0.5092574058 |
| 2013 | 40.13 | 3 | DIV_SI | 0.8256298228 |
| 2013 | 42.13 | 3 | DIV_SI | 0.4012970942 |
| 2013 | 43.13 | 3 | DIV_SI | 0.8277490095 |
| 2013 | 46.13 | 3 | DIV_SI | 0.8323862848 |
| 2013 | 47.13 | 3 | DIV_SI | 0.7471421087 |
| 2013 | 3.13 | 4 | DIV_SI | 0.7589851229 |
| 2013 | 7.13 | 4 | DIV_SI | 0.5457772484 |
| 2013 | 11.13 | 4 | DIV_SI | 0.6985249216 |
| 2013 | 14.13 | 4 | DIV_SI | 0.4641728754 |
| 2013 | 16.13 | 4 | DIV_SI | 0.7960150966 |
| 2013 | 22.13 | 4 | DIV_SI | 0.7792485988 |
| 2013 | 23.13 | 4 | DIV_SI | 0.7395289241 |
| 2013 | 26.13 | 4 | DIV_SI | 0.3535109894 |
| 2013 | 28.13 | 4 | DIV_SI | 0.8049079401 |
| 2013 | 36.13 | 4 | DIV_SI | 0.8143316937 |
| 2013 | 37.13 | 4 | DIV_SI | 0.7448026134 |
| 2013 | 38.13 | 4 | DIV_SI | 0.4395781076 |
| 2013 | 39.13 | 4 | DIV_SI | 0.5904028233 |
| 2013 | 40.13 | 4 | DIV_SI | 0.8251829695 |
| 2013 | 42.13 | 4 | DIV_SI | 0.4391237215 |
| 2013 | 43.13 | 4 | DIV_SI | 0.8337691511 |
| 2013 | 46.13 | 4 | DIV_SI | 0.8299362716 |
| 2013 | 47.13 | 4 | DIV_SI | 0.7289605071 |
| 2013 | 3.13 | 5 | DIV_SI | 0.7706495551 |
| 2013 | 7.13 | 5 | DIV_SI | 0.4867037901 |
| 2013 | 11.13 | 5 | DIV_SI | 0.6992367804 |
| 2013 | 14.13 | 5 | DIV_SI | 0.4414610597 |
| 2013 | 16.13 | 5 | DIV_SI | 0.8109023979 |
| 2013 | 22.13 | 5 | DIV_SI | 0.7783003921 |
| 2013 | 23.13 | 5 | DIV_SI | 0.744621146 |
| 2013 | 26.13 | 5 | DIV_SI | 0.3660573075 |
| 2013 | 28.13 | 5 | DIV_SI | 0.809785325 |
| 2013 | 36.13 | 5 | DIV_SI | 0.8020389264 |
| 2013 | 37.13 | 5 | DIV_SI | 0.7281186151 |
| 2013 | 38.13 | 5 | DIV_SI | 0.4355736639 |
| 2013 | 39.13 | 5 | DIV_SI | 0.6007013245 |
| 2013 | 40.13 | 5 | DIV_SI | 0.8222292379 |
| 2013 | 42.13 | 5 | DIV_SI | 0.4539647562 |
| 2013 | 43.13 | 5 | DIV_SI | 0.8375967574 |
| 2013 | 46.13 | 5 | DIV_SI | 0.8302254333 |
| 2013 | 47.13 | 5 | DIV_SI | 0.7092305296 |
