# Supplementary material for: Honey bee success predicted by landscape composition in Ohio, USA
Source: PeerJ. 2015 Mar 19;3:e838. doi: 10.7717/peerj.838 (PMC4369331; doi:10.7717/peerj.838)
Supplement: Supplemental Information S1 — Full spring survey questionnaire filled out by beekeepers in our study. [file peerj-03-838-s004.doc]

**Spring Survey**

To be completed after installing a package of bees

This is the first part of a two-part survey examining the success of honey bee colonies in urban, suburban and rural environments. If you complete this survey you will be contacted again in August and asked to complete a survey on the hive’s condition at that time. Your participation in this research survey is completely voluntary and you may withdraw at any time without penalty. Any information you provide through this survey will be considered confidential and will only be presented in a manner in which individual beekeepers are not identifiable. If you have any questions or concerns about the survey please contact Doug Sponsler ([sponsler.18@osu.edu](mailto:sponsler.18@osu.edu), 215-475-7203) or Reed Johnson ([johnson.5005@osu.edu](mailto:johnson.5005@osu.edu), 330-202-3523). For questions about your rights as a participant in this study or to discuss other study-related concerns or complaints with someone who is not part of the research team, you may contact Ms. Sandra Meadows in the Office of Responsible Research Practices at [1-800-678-6251](tel:1-800-678-6251). **We thank you for your participation!**


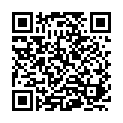
This survey can also be completed online at: [**http://surveys.cfaes.ohio-state.edu/cfaes/index.php?sid=72315&lang=en**](http://surveys.cfaes.ohio-state.edu/cfaes/index.php?sid=72315&lang=en) **or** [**http://bit.ly/z9KPBM**](http://bit.ly/z9KPBM) **or** through the QR code to the left.

**I. Eligibility (Please circle Yes or No)**

Are you 18 years of age or older? Yes No

Are you starting a colony with a package of bees this spring? Yes No

Is the apiary where your new colony will be placed located in the State of Ohio?

Yes No

Is your apiary in compliance with any local laws related to beekeeping? Yes No

**If you answered “No” to any of the above questions you are not eligible to participate in this survey.**

Is this apiary registered with the Ohio Department of Agriculture? Yes No

**If your apiary is not registered, please fill out the attached registration form and send it in to the Ohio Department of Agriculture within 10 days.**

**II. Location**

Where is your apiary located? Please provide one of the following: street address, latitude and longitude, or description of your apiary location so that it can be located on a map.

**III. Colony Information**

When did you install the package of bees?

**Date (mm/dd/yyyy):** _________

What race of queen came with your package of bees? (Circle one)

**Italian**

**Carniolan**

**Russian**

**I don’t know**

**Other (please describe below)**

How many total hives are in the apiary that contains the study hive?

**Number of hives:** _____

Provide a brief description of the apiary location (e.g. in a backyard, on a rooftop, next to apple orchard).

Which direction does the study hive face? (Circle one)

**North South East West Northeast Southeast**

**Northwest Southwest**

At what time in the morning does the study hive first receive direct sunlight on a sunny day?

**Time of day (nearest hour):** ________

At what time in the afternoon or evening does the study hive last receive direct sunlight on a sunny day?

**Time of day (nearest hour):** ________

**IV. Management Practices**

How many years have you been beekeeping?

**Number of years:** _______

What is the largest number of hives you have ever managed in a single year?

**Number of hives:** _______

**V. Follow up**

Did you place a sticker or leave a mark on the hive so that you know which hive is the “study hive”? (Circle one)

**Yes** **No**

Please provide an e-mail address so that we can contact you again in mid-August to follow up on this hive’s success.

**E-mail:**

If you would prefer to answer the summer follow-up survey by mail, please provide your name and mailing address:

**Name:**

**Street address / P.O. Box:**

**City, State ZIP:**

**Please return this survey to:**

**Beekeeper Survey Project**

**Thorne Hall**

**The Ohio State University – OARDC**

**1680 Madison Ave.**

**Wooster, OH 44691**
