# Supplementary material for: Honey bee success predicted by landscape composition in Ohio, USA
Source: PeerJ. 2015 Mar 19;3:e838. doi: 10.7717/peerj.838 (PMC4369331; doi:10.7717/peerj.838)
Supplement: Supplemental Information S2 — Full fall survey questionnaire filled out by beekeepers in our study. [file peerj-03-838-s005.doc]

**Summer Survey**

**To be completed during week of August 19-25**

**Instructions**

Thanks for filling out the survey about your new package this spring.  This is the follow-up survey to measure that colony's success up to this point.

**I. Hive status**

Are you looking at the same study hive that you looked at in the spring survey? (Circle one)

Yes No

Has this hive been moved since the previous survey? (Circle one)

Yes No

Is this hive still alive? (Circle one)

Yes No

**II. Queen status**

Does the study hive appear to be healthy? (Circle one and write any comments below)

Yes No I’m not sure

Is the hive currently queenless? (Circle one and write any comments below)

Yes No I’m not sure

Can you see any eggs or open brood in the colony? (Circle one and write any comments below)

Yes No I’m not sure

Can you see any of the capped brood? (Circle one and write any comments below)

Yes No I’m not sure

Did the study hive swarm? (Circle one)

Yes No

If yes, when did it swarm? (Circle one and write any comments below)

Date (mm/dd/yyyy): _______

Was the original queen replaced with a new queen that the bees produced either through emergency queen replacement or queen supersedure? (Circle one)

Yes No

If yes, when do you think the queen was replaced? (Circle one and write any comments below)

Date (mm/dd/yyyy): ________

Did you replace the queen yourself with a new queen or queen cell? (Circle one)

Yes No

If yes, when did you replace the queen? (Fill in date and comment below if possible)

Date (mm/dd/yyyy): _________

**III. Hive report**

What style of equipment is the hive? (Circle one and write any comments below)

10-frame 8-frame Top-bar Other

What does your hive consist of? (Check appropriate boxes working from the bottom of the hive to the top)

|  | Deep(9 5/8”) | Medium(6 5/8”) | Shallow(5 11/16”) | None |
| --- | --- | --- | --- | --- |
| Bottom |  |  |  |  |
| 2nd |  |  |  |  |
| 3rd |  |  |  |  |
| 4th |  |  |  |  |
| 5th |  |  |  |  |
| 6th |  |  |  |  |

When you were setting up this equipment, before you gave it to the bees, how many frames were drawn comb and how many were just foundation? (Write numbers in table below)

|  | Frames with just foundation | Drawn frames |
| --- | --- | --- |
| Bottom |  |  |
| 2nd |  |  |
| 3rd |  |  |
| 4th |  |  |
| 5th |  |  |
| 6th |  |  |

How many gallons of sugar syrup have you fed the hive since installing the bees?

Number of gallons: ______

Have you added or removed any frames from the colony? Did you add or remove honey frames? Did you equalize colonies by moving frames of brood and bees? If you are not sure about how many frames you moved, just give your best guess (Write numbers in table below)

|  | Honey frames removed | Honey frames added | Brood frames removed | Brood frames added |
| --- | --- | --- | --- | --- |
| Bottom |  |  |  |  |
| 2nd |  |  |  |  |
| 3rd |  |  |  |  |
| 4th |  |  |  |  |
| 5th |  |  |  |  |
| 6th |  |  |  |  |

Categorize all of the frames that are currently in each box in the hive. (Write numbers in table below)

|  | Undrawn foundation | Less than 1/2 drawn out | Mostly nectar or honey | Mostly pollen | Mostly brood | Drawn, but mostly empty |
| --- | --- | --- | --- | --- | --- | --- |
| Bottom |  |  |  |  |  |  |
| 2nd |  |  |  |  |  |  |
| 3rd |  |  |  |  |  |  |
| 4th |  |  |  |  |  |  |
| 5th |  |  |  |  |  |  |
| 6th |  |  |  |  |  |  |

Estimate the number of combs in each box that are more than half covered with bees on both sides. (Write numbers in table below)

|  | Frames more than half covered with bees |
| --- | --- |
| Bottom |  |
| 2nd |  |
| 3rd |  |
| 4th |  |
| 5th |  |
| 6th |  |

Have you used any beekeeping drugs since installing the package? (Circle any that apply and write any comments on the right)

Fumagilin B

Terramycin®

Tylan®

Check Mite®

Apistan®

Powdered sugar

ApiLife Var®

Apiguard®

MiteAway Quick Strip®

HopGuard®

Other (please describe below)

**IV. Follow up**

Are you interested in being contacted to complete another survey related to beekeeping sometime in the future? (Circle one)

Yes No
